# The osteology of *Triisodon crassicuspis* (Cope, 1882): New insights into the enigmatic “archaic” placental mammal group “Triisodontidae”

**DOI:** 10.1371/journal.pone.0311187

**Published:** 2024-11-11

**Authors:** William J. Toosey, Thomas E. Williamson, Sarah L. Shelley, Stephen L. Brusatte

**Affiliations:** 1 School of GeoSciences, University of Edinburgh, Edinburgh, Scotland, United Kingdom; 2 New Mexico Museum of Natural History and Science, Albuquerque, New Mexico, United States of America; Liverpool John Moores University, UNITED KINGDOM OF GREAT BRITAIN AND NORTHERN IRELAND

## Abstract

Following the end-Cretaceous mass extinction, mammals underwent an increase in body size, taxonomic diversity and ecological specialization throughout the Paleocene, exemplifying their adaptability. One especially enigmatic group is the “Triisodontidae”, medium- to large-sized ungulate-like placentals from the Paleocene which are best known from their teeth that exhibit adaptations towards carnivory. The “triisodontids” were the first large carnivorous mammals and pre-date, and may have given rise to, Mesonychia, a group of more specialized placental carnivores. The “triisodontids” have been well-described from dental material, although very little is known about their postcrania. Here, we describe the postcrania of *Triisodon crassicuspis–*the most completely represented species of the genus to date–from a specimen (NMMNH P-72096) recovered from basal Torrejonian strata of the Nacimiento Formation in the San Juan Basin, New Mexico. Anatomical comparisons reveal that the forelimb long bones of *Tri*. *crassicuspis* are robust relative to its size, more so than other “triisodontids”. Attachment sites on the ulna are evidence of well-developed muscles involved in powerful extension and flexion of the manus. In *Tri*. *crassicuspis*, the range of pronation-supination was limited as evident from the humeroradial morphology. Qualitative functional assessment of osteological features of the forelimb of *Tri*. *crassicuspis* is suggestive of terrestrial locomotion with at least moderate digging ability. Re-analyses of the dentition confirmed that *Tri*. *crassicuspis* had specializations for carnivory, and provide a body mass estimate of ca. 32–44 kg based on dental proxies. In summary, *Tri*. *crassicuspis* was a relatively large and powerful terrestrial animal, and one of the first known placentals to fill a largely carnivorous niche.

## Introduction

The end-Cretaceous mass extinction around 66 million years ago (Ma), which is attributed to the impact of a massive asteroid in what is now the northern Yucatán Peninsula [1,2], and led to the disappearance of >75% of all animal and plant species [1,3], heralded the start of the Cenozoic Era. Non-avian dinosaurs perished during this event and its aftermath, and the diversity of many other taxa, such as insects [4], teleost fish [5], squamates [6], birds [7] and mammals [8], was drastically reduced. The landscape that remained for these taxa was ravaged and inhospitable, yet it is from this environment that mammals rose to prominence. Within only 300,000 years of the mass extinction, local mammalian diversity had recovered and regional diversity had approximately doubled since the end-Cretaceous [8]. During the Paleocene epoch (ca. 66–56 Ma), mammals—particularly placentals that were capable of giving live birth to relatively well-developed young—underwent an explosive adaptive radiation, increasing in body size, taxonomic diversity and ecological specialization [8–10]. The most diverse groups at that time were stem members of extant clades [10].

The great majority of Paleocene mammals are “archaic” (the word “archaic” meaning that they have no surviving descendants [11])—they do not clearly belong to any extant group and their phylogenetic affinities to extant species remains poorly understood. One poorly studied and particularly enigmatic Paleocene mammal group is the “Triisodontidae”—morphologically robust and medium- to large-sized ungulate-like placental mammals. Their fossils are typically poorly represented, being known primarily from teeth, along with several partial skulls and limited associated postcranial fragments for some taxa. The “triisodontids” are generally rare elements of the Puercan (ca. 66−63.5 Ma) and Torrejonian (ca. 63.5−62 Ma) faunas of the San Juan Basin, but some have also been reported from other Paleocene localities throughout western North America [12,13]. At least one taxon, *Mondegodon eutrigonus*, has been reported from Portugal [14], although whether this is a “triisodontid” remains controversial. All undisputed “triisodontids” are known only from the early Paleocene of western North America, except that *Goniacodon levisanus* has been reported from the very early part of the late Paleocene of Montana [13].

The “triisodontids” were among the first placental mammals to evolve adaptations for carnivory. Their higher-level phylogenetic relationships remain highly uncertain, although they were likely a paraphyletic stem to the Mesonychia, a familiar group of extinct carnivorous ungulates [14–16]. Some taxa like the genus *Triisodon* were among the largest mammals of the early Paleocene [17,18]. This genus is represented in North America by *Tri*. *quivirensis* and a smaller, poorly known taxon, *Tri*. *crassicuspis* [19]. The “triisodontids” attained a maximum standing taxonomic diversity of 8 species, along with a broad variety of sizes, within approximately 0.5 Ma of the K-Pg boundary, during the early to middle Puercan (ca. 66–65.3 Ma). The group declined in diversity thereafter and went extinct near the start of the Tiffanian (ca. 62–57.5 Ma), without leaving any modern descendants [20].

In order to shed light on this group, we report on a new specimen (NMMNH P-72096) of *Tri*. *crassicuspis* that includes a nearly complete upper and lower dentition, and associated partial skull and postcrania. These elements were found in association and therefore represents one individual, and were recovered from the Nacimiento Formation at the San Juan Basin, New Mexico, USA during the 2015 field season. The newly described specimen is partially covered with concretion. It was possibly fragmented and crushed prior to fossilization. It belongs to an adult individual since the permanent teeth are present and are fully erupted, and the epiphyses of the long bones are fused. We present a comprehensive description of its dentition and osteology that additionally allowed us to interpret its functional morphology and estimate its body mass. The significance of this study lies in the fact that *Tri*. *crassicuspis* is an extremely rare fossil taxon, with NMMNH P-72096 being only the third specimen ever found since local frontiersman David Baldwin collected the lectotypes of *Tri*. *crassicuspis* and *Tri*. *rusticus* from the San Juan Basin in the early 1880s, consisting only of lower jaw fragments with damaged molars. Unfortunately, the precise stratigraphic horizon as well as the location for the latter two specimens is unknown. A comprehensive and up-to-date description for those specimens is also provided (AMNH 3178 [21] and AMNH 3225 [22]).

### Institutional abbreviations

AMNH, American Museum of Natural History, New York City, New York, USA

DMNH.EPV., Denver Museum of Natural History, Denver, Colorado, USA

IVPP, Institute of Vertebrate Paleontology and Paleoanthropology, Beijing, China

MNHN, Muséum National d’Histoire Naturelle, Paris, France

MHNT, Muséum d’Histoire Naturelle de Toulouse, Toulouse, France

NMMNH, New Mexico Museum of Natural History and Science, Albuquerque, New Mexico, USA

NMS, National Museum of Scotland, Edinburgh, Scotland, UK

PSS, Paleontological and Stratigraphy Section (Geological Institute), Mongolian Academy of Sciences, Ulaan Baatar, Mongolia

UM, University of Michigan Museum of Paleontology, Ann Arbor, Michigan, USA;

USGS, U.S. Geological Survey, Denver, Colorado, USA

USNM, National Museum of Natural History, Washington, D.C., USA

YPM-PU, Princeton Collection, Yale Peabody Museum, New Haven, Connecticut, USA.

### Historical background

#### Systematic history of “Triisodontidae”

At present, the family “Triisodontidae” consists of five genera: *Carcinodon*, *Goniacodon*, *Eoconodon*, *Triisodon* and *Oxyclaenus* [14,15,23,24]. The phylogenetic position of the “Triisodontidae” is highly contentious and has yet to be completely resolved. The group was initially placed within the carnivoran-related order “Creodonta” [21,24–27]. Then in 1909, both the “Triisodontidae” and Mesonychidae were grouped with the Oxyclaenidae in the suborder Acreodi [28]. In 1915, the “Triisodontidae’’ were moved to a position within the “Arctocyonidae”, the latter family which was also part of the “Creodonta” [29]. In 1966, the “Arctocyonidae” along with the Mesonychidae were transferred from the Creodonta to the “Condylarthra” (the “archaic” ungulates) [30]. “Triisodontids” have been considered by some to be a subfamily of the more omnivorous “Arctocyonidae” called the "Triisodontinae" [31–34]. The "triisodontines" are now generally classified at family level for exhibiting incipient dental trends that became more advanced in the mesonychians [18,35,36]. “Triisodontids” were believed by Matthew [37] to have been ancestral to the Mesonychia, a clade that was previously thought to be most closely related to Cetacea [33]. The position of the Mesonychia itself remains unresolved, with Spaulding *et al*. [38] producing evidence for reconstructions whereby Mesonychia is either nested within Cetaceamorpha, or excluded from Artiodactyla entirely, citing the need for broader taxon sampling to resolve this. Even in these highly contradictory scenarios, the “Triisodontidae” (represented by *E*. *heilprinianus (= coryphaeus)* in their dataset) are consistently found as being closely associated with the Mesonychia.

Regarding the higher-level phylogenetic relationships of "triisodontids", Van Valen [34] suggested that the “Triisodontinae” and the “Arctocyoninae” evolved separately from a clade comprised of *Protungulatum* and *Oxyprimus* (termed Oxyclaeninae but that name later fell out of favor [15]. Van Valen suggested that the “Triisodontinae” was sister to the “Arctocyoninae” and considered *Oxyclaenus* to be the most basal “arctocyonine”.

In a cladistic analysis of ungulate affinities, Prothero *et al*. [39] suggested that “Triisodontidae” forms a paraphyletic grade on the line to Mesonychia, with *Oxyclaenus* being the most basal taxon. In that analysis, *Triisodon* is sister to *Paratriisodon*, the latter taxon which is now a junior synonym of *Andrewsarchus* [14,40]. Later, in a phylogenetic analysis by Williamson and Carr [15], *Oxyclaenus* was regarded as the sister genus to *Andrewsarchus*, the latter taxon which for a long time was tentatively considered a “triisodontid”. Recent phylogenetic studies using morphological data have found that *Andrewsarchus* has no mesonychian affinities and is a basal artiodactyl closely related to entelodonts [38,41].

In a recent phylogenetic analysis by Tabuce *et al*. [14], the “triisodontids” were grouped with the mesonychids in the basal placental order Acreodi (a now abandoned term that comprises mesonychians and some “arctocyonids”), and the “Arctocyonidae” formed a paraphyletic grade on the line to Acreodi. Williamson and Carr [15] and Tabuce *et al*. [14] considered *Tri*. *crassicuspis* to be closely related to *Goniacodon*. Halliday *et al*. [36] carried out a cladistic analysis of Paleocene placental mammals using parsimony methods, having also found evidence for the placement of “Triisodontidae’’ within the order Mesonychia, which is recovered as a sister group to the “Arctocyonidae”, and regarded as non-ungulates. The breadth of their cladistic analysis represents the largest ever directed towards figuring out the phylogenetic relationships of Paleocene mammals, although many of the results are problematic, including pteropodid bats being placed between mesonychians and modern ungulates. A follow-up study by Halliday *et al*. [42], which updated the phylogenetic dataset from Halliday *et al*. [36] via increased taxon sampling, and combined morphological and molecular data, was conducted to help reconcile aspects of such a phylogeny. Their results found that “triisodontids” are still placed within Mesonychia.

#### Taxonomic history of *Triisodon*

The first scientific account of a “triisodontid” was by Cope [25], who diagnosed the genus *Triisodon* based upon the dentary of a sub-adult individual, giving it the binomial *Tri*. *quivirensis*. This specimen was collected amongst other Paleocene mammal fossils by David Baldwin (who did not provide specific information on the age and locality) somewhere in the Paleocene of the San Juan Basin of northwestern New Mexico [43]. Cope subsequently named and described other “triisodontid” taxa based on poorly preserved dentary fragments with lower molars, including *Tri*. *crassicuspis* (originally named *Conoryctes crassicuspis*) [21], *Tri*. *heilprinianus* [21], *Sarcothraustes antiquus* [44], *Tri*. *rusticus* [22], *Sa*. *coryphaeus* [26] and *Tri*. *biculminatus* [26].

*Tri*. *biculminatus* was considered a subjective junior synonym of *Tri*. *heilprinianus* by Matthew [45], who stated that “the apparent differences are almost entirely due to the biological age of the individual”. Matthew and Granger [46] concluded that *Tri*. *quivirensis* was not congeneric with *Tri*. *heilprinianus* and that *Sarcothraustes* is a junior synonym of *Triisodon*. They proposed the new genus *Eoconodon* for *Sa*. *coryphaeus* and synonymized *E*. *coryphaeus* with *“Sa*.*” heilprinianus*. They also agreed with Matthew [45] in regarding *Tri*. *biculminatus* to be a synonym of *E*. *heilprinianus*. Other workers have questioned the number of *Triisodon* species, with *Tri*. *antiquus* likely being a junior synonym of *Tri*. *quivirensis* [34,47]. *E*. *heilprinianus* has since been found to be a synonym of *C*. *comma* [34], which is why the name *E*. *coryphaeus* is used.

However, Tomida [48] suggested that *Tri*. *quivirensis* and *Tri*. *antiquus* were distinct, with the former being distinguished by its slightly smaller size and more reduced paraconid on the lower molars. Taylor [49] suggested that most of the variation in dental features observed in specimens referable to *Triisodon* could be explained by individual variation. For example, he suggested that NMMNH P-51329 (an isolated molar) is similar in size to AMNH 3352, the lectotype of *Tri*. *quivirensis*, but has a large and distinct paraconid and metaconid on the third molar. He therefore suggested that the relative size of the paraconid and metaconid was an invalid feature and that *Tri*. *quivirensis* and *Tri*. *antiquus* could not be distinguished by a difference in size alone. *Tri*. *rusticus* was transferred to the genus *Goniacodon* by Scott [24]. *C*. *crassicuspis* was reassigned from *Conoryctes* to *Sarcothraustes* by Scott [24], then subsequently as *Tri*. *crassicuspis* by Matthew [31], before eventually being listed as *Go*. *crassicuspis* by Van Valen [34], the latter two studies which did not provide evidence. Matthew [31] also synonymized the species *Tri*. *rusticus* with *Tri*. *crassicuspis*, but again, no justification was provided.

**Fig 1:**
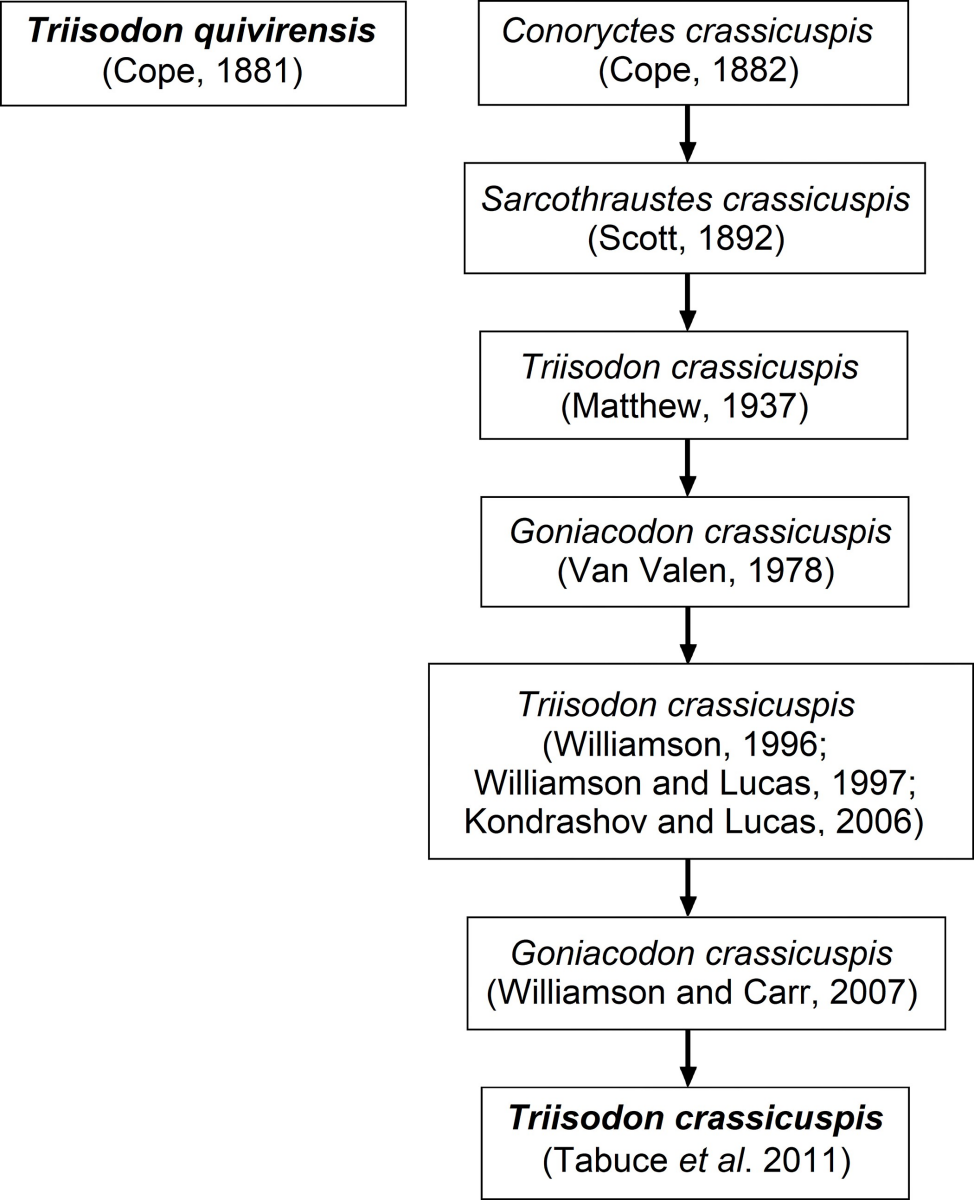
Historic changes in naming and classification of the 2 currently recognized species of *Triisodon*.

Later on, Williamson [19], Kondrashov and Lucas [17], disagreed with Van Valen [34], arguing that *Go*. *crassicuspis* belongs within the genus *Triisodon*. Williamson and Lucas [43] fleetingly described some upper dentitions from the Chico Springs locality, including a P4 and a M1, which they referred to *Tri*. *crassicuspis* and suggested that the specimen has characters that support its classification within *Triisodon*. Williamson and Carr [15] then favored using the name *Go*. *crassicuspis* according to a phylogenetic analysis, but the species was again most recently transferred to the genus *Triisodon* by Tabuce *et al*. [14], who provided no justification. This is where the genus *Triisodon* currently stands, with two species: *Tri*. *quivirensis* and *Tri*. *crassicuspis*. The history of *Triisodon* naming conventions is summarized in Fig 1.

#### Geological context

*Triisodon* has been documented from the Torrejonian (To1-3) strata of western North America, with specimens yielded from the Nacimiento Formation of the San Juan Basin in northwestern New Mexico, USA [43,48,50,51], and also the Big Bend National Park of western Texas [52]. During the Paleocene, the climate in what is now the San Juan Basin was more humid and hotter than today, with the mid-latitudes experiencing subtropical climates during the Puercan and Torrejonian that became cooler by the Tiffanian [53]. The mean annual precipitation was about 1,100 mm in what is now the San Juan Basin, and there were mean annual temperatures of roughly 12° ± 4.4° C [54].

The San Juan Basin preserves an exceptional fossil record of terrestrial vertebrate succession for the early Paleocene, including type faunas for the first two North American Land Mammal Ages (NALMAs)—the Puercan (66–64 Ma) and Torrejonian (64–61 Ma) [19,55,56]. The Nacimiento Formation consists of non-marine lacustrine and fluvial beds, and has a thickness of up to approximately 525 m [57]. The beds consist predominantly of mudstones and sandstone, plus well-developed paleosols [58]. The flora of the Nacimiento Formation largely consisted of a diverse range of angiosperms, and C3 plants were prevalent in the ecosystem [59]. This suggests moderate sunlight intensity and temperatures, along with plentiful groundwater and high levels of carbon dioxide [60].

#### Age and locality

**Fig 2:**
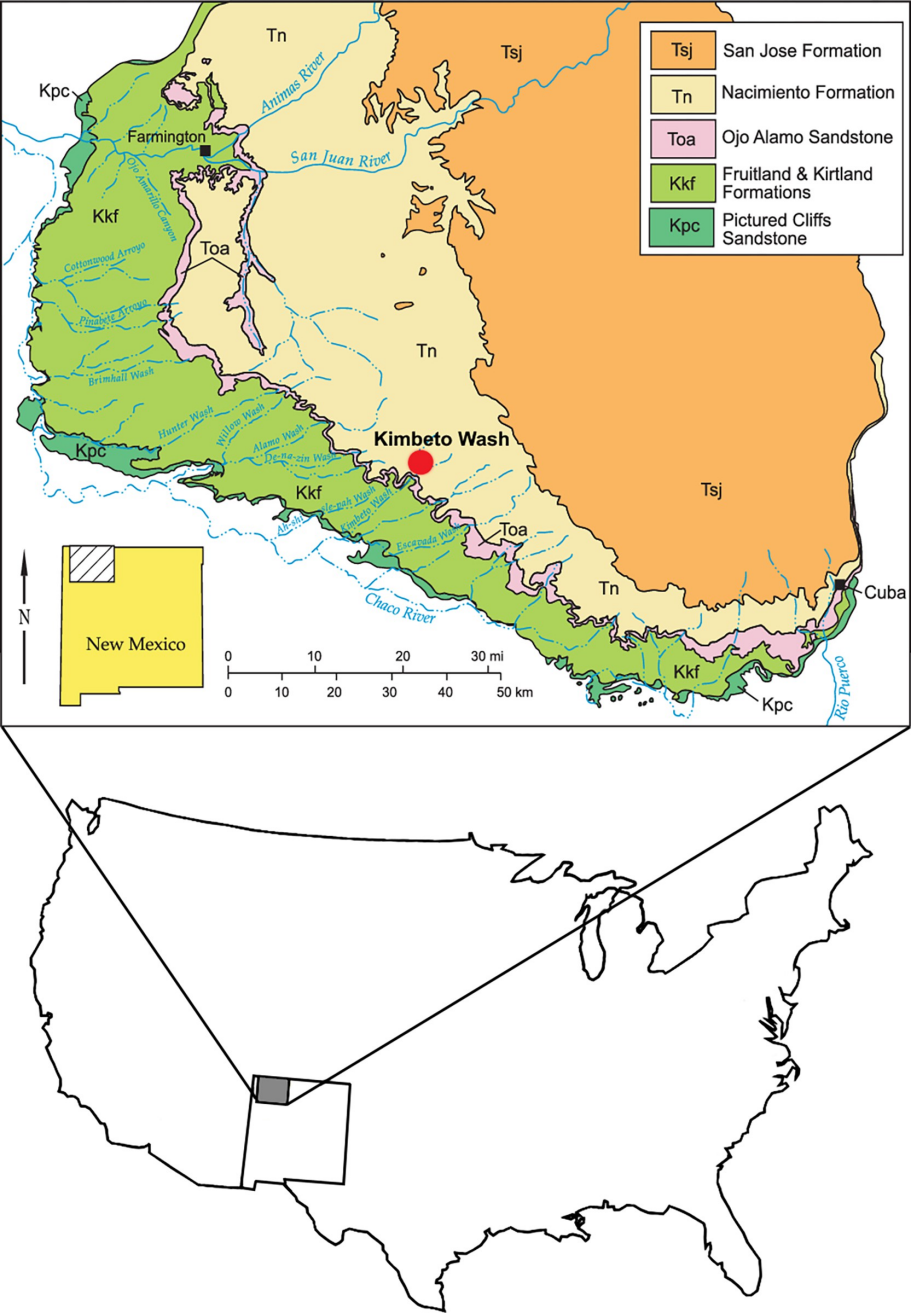
Geological map of the San Juan Basin showing the location of Kimbeto Wash relative to the outcrop belt of the Nacimiento Formation, San Juan Basin, New Mexico. After Williamson [19].

*Triisodon* is known only from the Torrejonian (c. 63.5 to 62 Ma), early Paleogene [19], from four of the six subdivisions of the Nacimiento Formation according to Flynn *et al*. [61]. These are the Arroyo Chijuillita Member, Tsosie Member, Kutz Member and Ojo Encino Member (sensu Cather *et al*. [58]). The new specimen (NMMNH P-72096) of *Tri*. *crassicuspis* was recovered from early Paleocene strata of the Tsosie Member at the NMMNH locality L-9874 in Kimbeto Wash in the San Juan Basin, New Mexico, USA (Fig 2). These strata are from the *Periptychus carinidens—Protoselene opisthacus* zone of Williamson [19] and are within a zone of normal magnetic polarity correlated with C28n [56]. This interval is relatively poorly fossiliferous in the Nacimiento Formation. *Tri*. *quivirensis*, the largest known “triisodontid”, has a first appearance in basal Torrejonian strata (Tj1) of the Nacimiento Formation and last appears in the *Pantolambda cavirictum—Mixodectes pungens* zone, prior to the end of the Torrejonian NALMA [19]. The specimen NMMNH P-72096 suggests that a second smaller species of *Triisodon*, *Tri*. *crassicuspis*, was present in the basal Torrejonian and co-occurred with the larger species. We speculate that *Tri*. *crassicuspis* may be restricted to the very earliest Torrejonian of the San Juan Basin because that is were the new specimen was found and the preservation is similar to that of the lectotypes of *Tri*. *crassicuspis* and *Tri*. *rusticus*. The earliest Torrejonian horizon is very poorly sampled which at least partly explains the poor representation of *Tri*. *crassicuspis* in existing collections. Based on their temporal distribution in the Nacimiento Formation, *Triisodon* only overlaps with *Dissacus*, a mesonychid, briefly in the Tj5 interval–during which both *Tri*. *quivirensi*s and *D*. *navajovius* are rare. During Tj6 (latest Torrejonian), both *Dissacu*s and *Ankalagon* are present (*Triisodon* is absent) and they overlap stratigraphically with *Goniacodon* and *Oxyclaenus*.

## Materials and methods

This description is mostly based on direct examination of the specimen NMMNH P-72096. Within the morphological description of this specimen, detailed osteological comparisons are drawn between other “triisodontids” and various other Paleocene mammal taxa, represented by dental, cranial and postcranial material. These include: *Goniacodon levisanus* (AMNH 7798, USNM 15503, NMMNH P-16377), *Eoconodon coryphaeus* (AMNH 3177, AMNH 16329, DMNH.EPV.130976, AMNH 764), *E*. *gaudrianus* (AMNH 4029), *Triisodon quivirensis* (NMMNH P-20918, USNM 22483 and AMNH 16559), *Oxyclaenus cuspidatus* (NMMNH P-22034), *Periptychus carinidens* [62], *Arctocyon primaevus* [63], *Ar*. *mumak* [64] and various mesonychids. These mesonychids are *Dissacus navajovius* [65], *"D*.*" praenuntius* [65], *"D*.*" europaeus* [66], *Ankalagon saurognathus* [65], *Hyaenodictis raslanloubatieri* [16], *Sinonyx jiashanensis* [65] and *Pachyaena ossifraga* [67]. All of these fossil taxa were chosen on the basis of their reconstructed close relationship to *Tri*. *crassicuspis* or because they are represented by a relatively complete array of specimens. A Cretaceous eutherian of the taxon *Maelestes gobiensis* is used as a more distantly related comparison [68].

Additionally, comparisons with four taxonomically diverse medium-sized extant mammals showing various locomotor modes were made first hand using the collections of the National Museum of Scotland. The collections provided complete skeletons of the ursid *Tremarctos ornatus* (a scansorial animal [69]), the mustelids *Gulo gulo* (a terrestrial climber [70] and *Meles meles* (a semi-fossorial animal [70]), and the orycteropodid afrotherian *Orycteropus afer* (a semi-fossorial animal [71,72]. All these extant taxa were chosen because they exhibit prevalent robust morphologies, similar to those of many Paleocene mammals such as "triisodontids", and exhibit analogous osteological features with *Tri*. *crassicuspis*, which provide useful comparisons.

All measurements were obtained to the nearest 0.1 mm, directly from specimens with a digital caliper. Terminology for tooth cusps adheres to the protocols indicated by Williamson and Carr [15], St Clair *et al*. [13] and Clemens *et al*. [12]. For discussion of the dentition, the notation is based on O’Leary *et al*. [73] and Shelley *et al*. [62], in which the premolars are numbered as P1/p1, P2/p2, P4/p4 and P5/p5, since P3/p3 has been evolutionarily lost in Placentalia [73]. The dental notation for *Tri*. *crassicuspis* is thus: c1/C1, P2/p2, P4/p4, P5/p5, M1/m1, M2/m2 and M3/m3. Osteological nomenclature for the skull follows Wible and Rougier [74], Wible and Spaulding [75], and *Miller’s Anatomy of the Dog*, *4th edition* [76]. Postcranial osteological terminology essentially follows Evans and DeLahunta [76], which incorporates English translations of the Latin from *Nomina Anatomica Veterinaria*. Unless otherwise specified, the fossil elements were photographed in standard anatomical views. Many of the descriptive portions are made from the specimen with photographs and annotated diagrams of the most complete and/or best preserved skeletal elements available. We estimated the body mass of *Tri*. *crassicuspis* via a series of regression equations using dental [77–79] and postcranial proxies [80] used on NMMNH P-72096, to provide a range of dental estimates relative to the estimate calculated on the basis of the minimum humeral circumference. We used a digital calliper for taking dental measurements in millimetres, to the nearest 0.1 mm, and we used a vinyl tape for measuring the minimum humeral circumference.

This research did not involve human or animal subjects, and conforms to the ethical guidelines of the University of Edinburgh School of GeoSciences.

Dental measurements are included within Table 1, cranial and mandibular measurements within Table 2, and postcranial measurements within Table 3.

**Table 1:** Measurements of the dentition of *Triisodon crassicuspis*.

Tooth	Measurement (mm)
C1 (NMMNH P-72096), proximodistal length	72.8
C1 (NMMNH P-72096), mesiodistal width at base	13.7
C1 (NMMNH P-72096), mesiodistal width at center	19.6
c1 (NMMNH P-72096), mesiodistal width at base	16.6
P2 (NMMNH P-72096), mesiodistal length	8.2
P2 (NMMNH P-72096), buccolingual width	5.3
P4 (NMMNH P-72096), mesiodistal length	8.5
P4 (NMMNH P-72096), buccolingual width	7.8
P5 (NMMNH P-72096), mesiodistal length	10.6
P5 (NMMNH P-72096), buccolingual width	11.9
M1 (NMMNH P-72096), mesiodistal length	11.0
M1 (NMMNH P-72096), buccolingual width	13.8
M2 (NMMNH P-72096), mesiodistal length	11.9
M2 (NMMNH P-72096), buccolingual width	16.9
M3 (NMMNH P-72096), mesiodistal length	7.6
M3 (NMMNH P-72096), buccolingual width	12.6
m1 (NMMNH P-72096), mesiodistal length	12.8
m1 (NMMNH P-72096), buccolingual width	9.2
m1 (AMNH 3225), mesiodistal length	11.7
m1 (AMNH 3225), mesiodistal length	7.3
m1 (AMNH 3225), apicobasal length of trigonid	5.9
m1 (AMNH 3225), apicobasal length of talonid	3.6
m2 (NMMNH P-72096), mesiodistal length	13.9
m2 (NMMNH P-72096), buccolingual width	10.1
m2 (AMNH 3225), mesiodistal length	16.3
m2 (AMNH 3225), buccolingual width	8.6
m3 (NMMNH P-72096), mesiodistal length	12.7
m3 (NMMNH P-72096), buccolingual width	6.0
m3 (AMNH 3225), mesiodistal length	12.3
m3 (AMNH 3225), buccolingual width	9.3
m3 (AMNH 3225), apicobasal length of trigonid	7.3
m3 (AMNH 3225), apicobasal length of talonid	3.6

**Table 2:** Measurements of the cranial and mandibular elements of *Triisodon crassicuspis* (NMMNH P-72096).

Element	Measurement (mm)
Maxilla, dorsoventral height of zygomatic arch at root	23.5
Squamosal, anteroposterior diameter of glenoid fossa	17.0
Squamosal, mediolateral diameter of glenoid fossa	33.5
Squamosal, proximodistal height of glenoid fossa	11.5
Squamosal, proximodistal height of preglenoid process	6.8
Squamosal, proximodistal height of postglenoid process	11.5
Left hemi-mandible, dorsoventral depth of left mandibular body from below p2 back to m2	40.1
Left hemi-mandible, Dorsoventral depth of retromolar gap	5.9

**Table 3:** Measurements of the postcrania of *Triisodon crassicuspis* (NMMNH P-72096).

Element	Measurement (mm)
Humerus, anteroposterior depth of proximal articular surface	22.5
Humerus, mediolateral width of proximal articular surface	43.0
Humerus, proximodistal length of head	29.3
Humerus, mediolateral width of head	28.7
Humerus, mediolateral width of humeral trochlea in posterior view	13.7
Humerus, mediolateral width of humeral trochlea in distal view	13.3
Humerus, mediolateral width of capitulum	19.1
Humerus, proximodistal length of capitulum in posterior view	17.2
Humerus, anteroposterior depth of greater tubercle	21.6
Humerus, mediolateral width of greater tubercle	15.6
Ulna, anteroposterior depth of diaphysis	19.8
Ulna, anteroposterior depth of the fossa for the *m*. *abductor pollicis longus*	11.7
Radius, mediolateral width of proximal articular surface	21.7
Radius, anteroposterior depth of proximal articular surface	13.9
Radius, height of capitular eminence	5.7
Radius, proximodistal length of bicipital tuberosity	13.2
Radius, mediolateral width of diaphysis	12.2
Femur, mediolateral diameter of head	25.2

### Specimens examined

**Fig 3:**
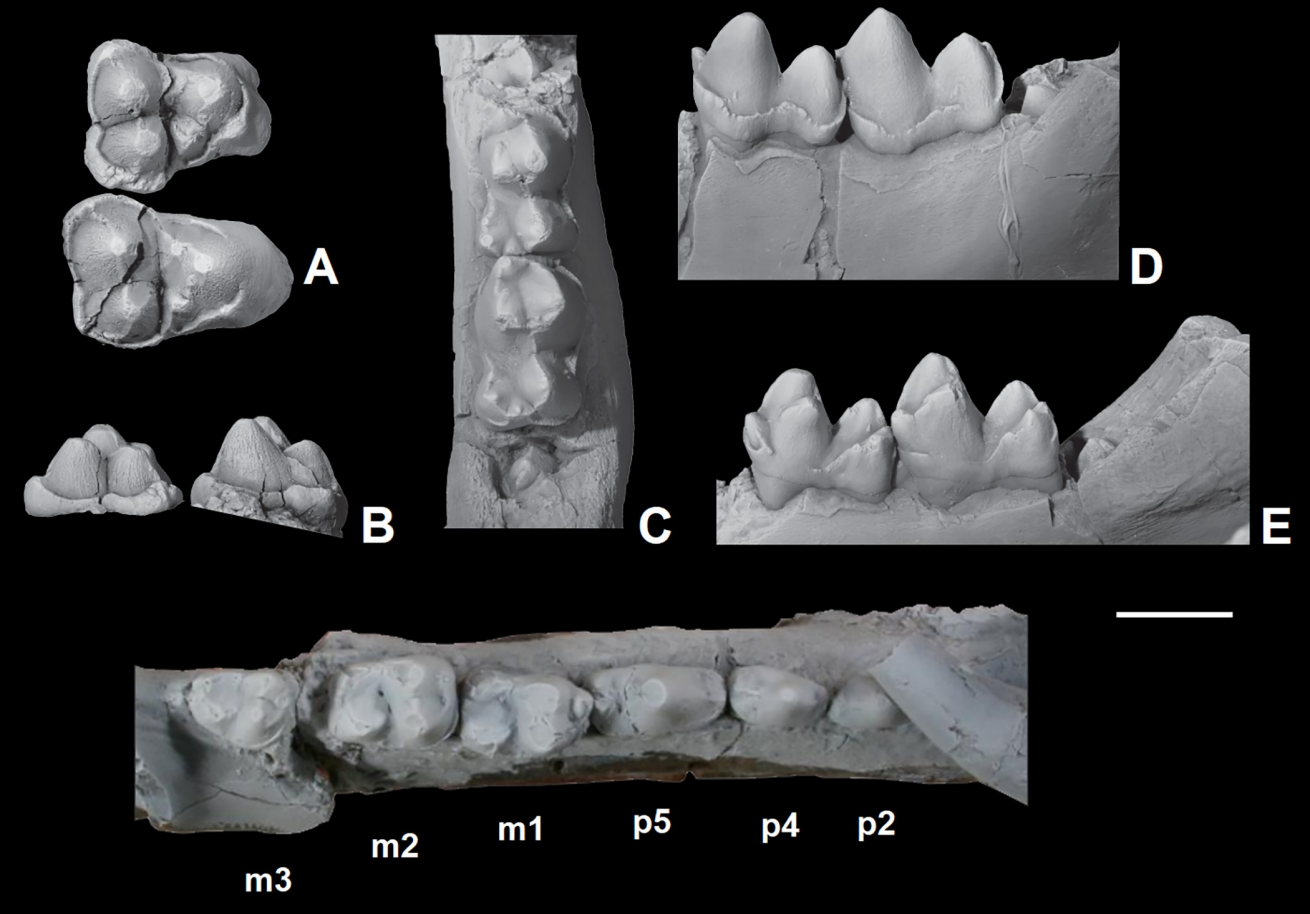
The dentition of other “triisodontids”. M1-2/m1-2 of *Triisodon quivirensis* (NMMNHP-20918). A-B, right M1-2, NMMNH P20918, in occlusal (A) and buccal (B) views. Right partial dentary with m1-2, AMNH 3352, in occlusal (C), buccal (D), and lingual (E) views. (F) The dentition of *Goniacodon levisanus* (AMNH 7798) in occlusal view. Scale bar: 10 mm.

The dentition of *Triisodon crassicuspis* was compared to other “triisodontids” including *Tri*. *quivirensis* (NMMNH P-20918, Fig 3A–3E) and *Goniacodon levisanus* (AMNH 7798, Fig 3F).

**Fig 4:**
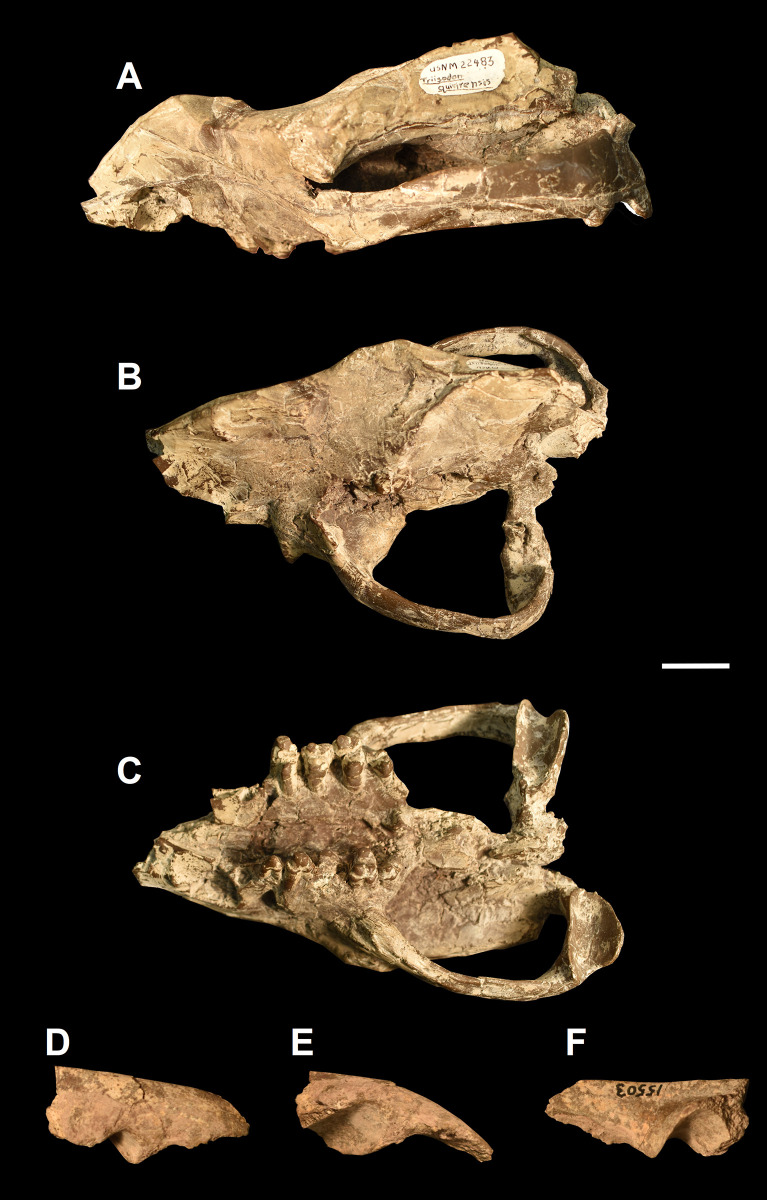
Cranial material of other “triisodontids”. The skull of *Triisodon quivirensis* (USNM 22483) (A-C): (A) lateral view; (B) dorsal view; (C) ventral view. The right posterior zygomatic arch of *Goniacodon levisanus* (D-F): (D) lateral view; (E) ventral view; (F) medial view. Scale bar: 30 mm.

We compared the skull of *Tri*. *crassicuspis* to that of a few other “triisodontids” and other closely related “condylarths” for which equivalent bones have been preserved. Taxa we made comparisons to include: *Eoconodon coryphaeus* (DMNH.EPV.130976, figured in [3]), *Tri*. *quivirensis* (USNM 22483, Fig 4A–4C), *Go*. *levisanus* (USNM 15503, Fig 4D–4F) and *Arctocyon primaevus* (MNHN.F.CR700, figured in [63]). Two near complete skulls are known for *E*. *coryphaeus*: one described by Matthew [31], and one recently recovered from the Corral Bluffs Site at the Denver Basin in Colorado [3]. In addition to paired maxillae, the skull of *Go*. *levisanus* is represented only by the right squamosal preserving the posterior zygomatic arch.

**Fig 5:**
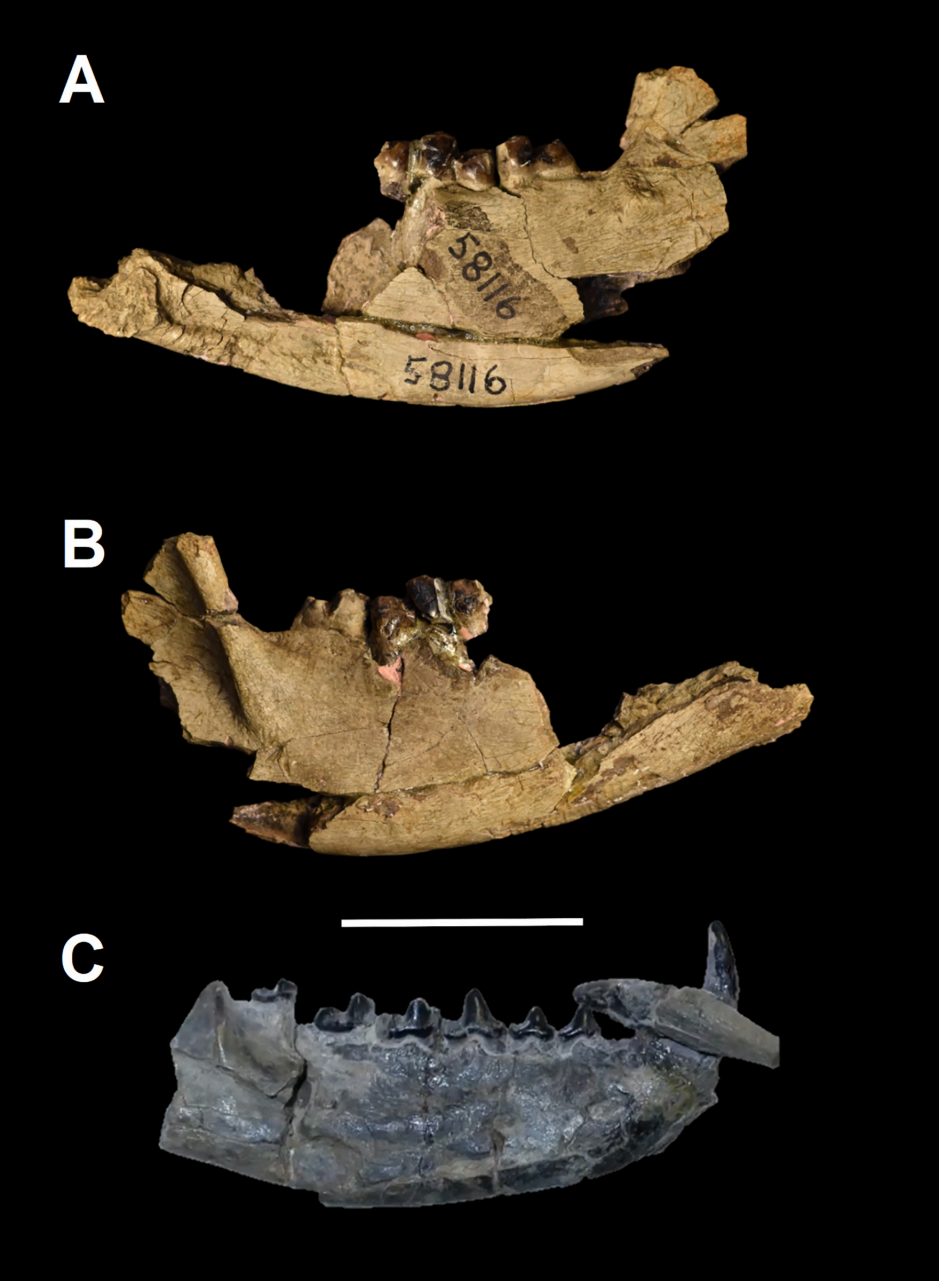
Hemi-mandibles of other “triisodontids”. Partial left mandible of *Eoconodon gaudrianus* (AMNH 58116) (A-B): (A) lateral view; (B) medial view. (C) Partial right dentary of *Goniacodon levisanus* (AMNH 7798) in lateral view. Scale bar: 30 mm.

The mandible of *Tri*. *crassicuspis* was compared to *Tri*. *quivirensis* (AMNH 3352, figured in [17]), *Ar*. *primaevus* (MNHN.F.CR2, figured in [63]), *E*. *coryphaeus* (AMNH 16329, figured in [3]), *E*. *gaudrianus* (AMNH 58116, Fig 5A and 5B), *Go*. *levisanus* (AMNH 7798, Fig 5C), *“Dissacus” praenuntius* (UM 69305, figured in [65]) and *Sinonyx jiashanensis* (IVPP V10760, figured in [65]).

**Fig 6:**
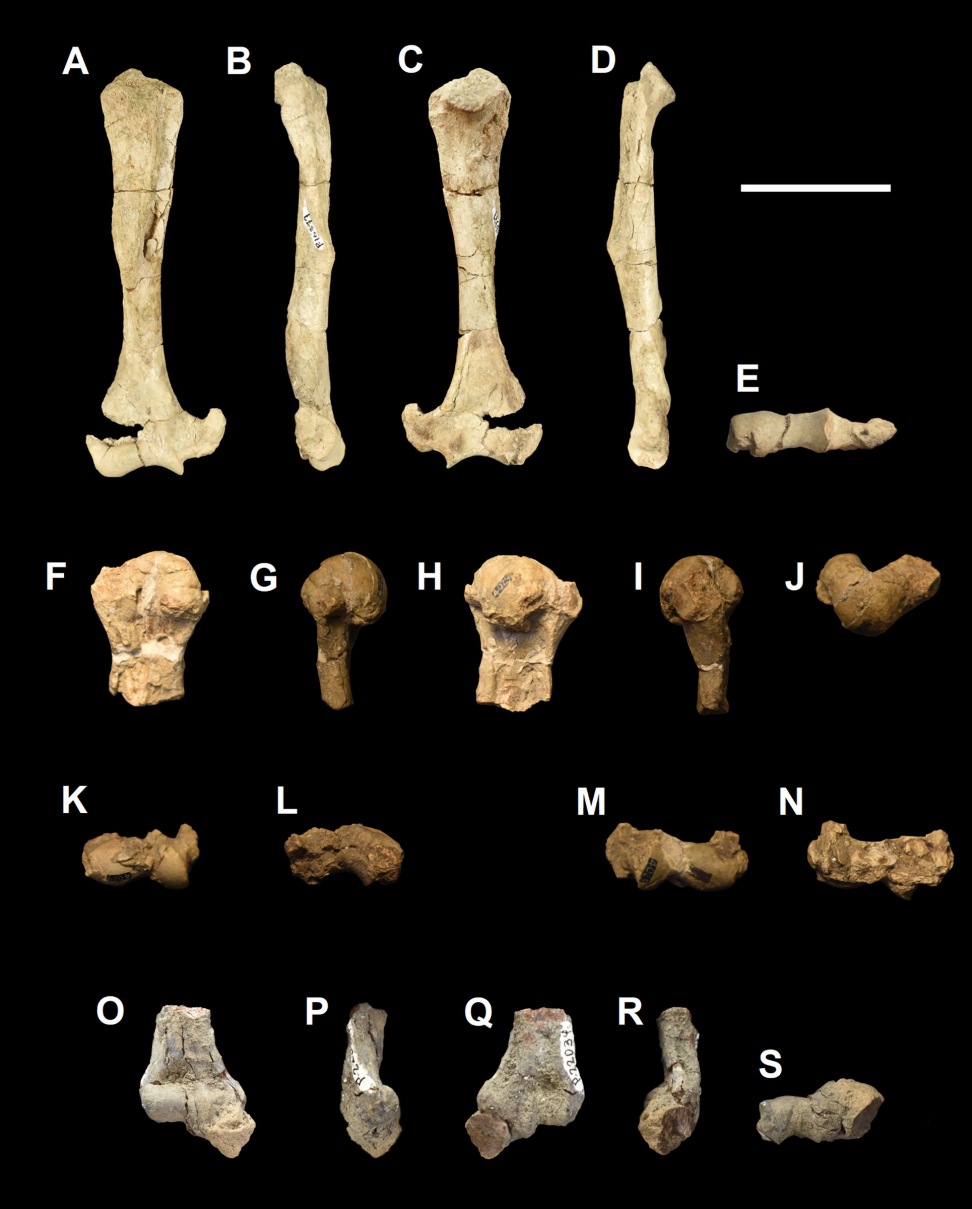
Humeri of medium-sized “triisodontids”. Partial right humerus of *Goniacodon levisanus* (NMMNH P-16377) (A-E): (A) anterior view; (B) lateral view; (C) posterior view; (D) medial view; (E) distal view. Right proximal humerus of *Eoconodon gaudrianus* (AMNH 4029) (F-J): (F) anterior view; (G) lateral view; (H) posterior view; (I) medial view; (J) proximal view. Left distal humerus of *Eoconodon gaudrianus* (AMNH 4029) (K-L): (K) anterior view; (L) posterior view. Right distal humerus of *Eoconodon gaudrianus* (AMNH 4029) (M-N): (M) anterior view; (N) posterior view. Right distal humerus of *Oxyclaenus cuspidatus* (NMMNH P-22034) (O-S): (O) anterior view; (P) lateral view; (Q) posterior view; (R) medial view; (S) distal view. Scale bar: 30 mm.

**Fig 7:**
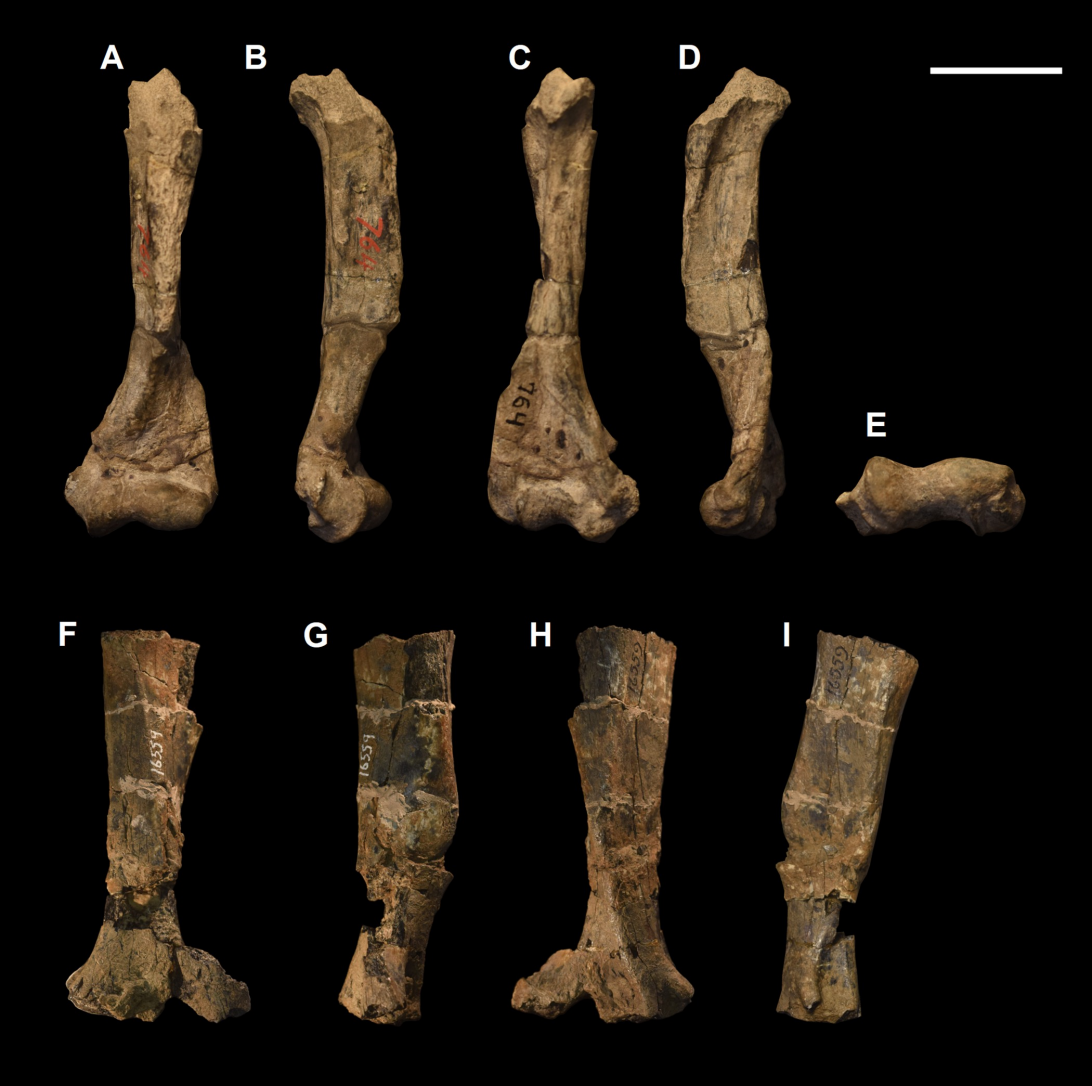
Humeri of other large-sized “triisodontids”. Partial right humerus of *Eoconodon coryphaeus* (AMNH 764) (A-E): (A) anterior view; (B) lateral view; (C) posterior view; (D) medial view; (E) distal view. Partial right humerus of *Triisodon quivirensis* (AMNH 16559) (F-I): (F) anterior view; (G) lateral view; (H) posterior view; (I) medial view. Scale bar: 30 mm.

The humerus of *Tri*. *crassicuspis* was compared to the following extinct taxa: *Go*. *levisanus* (NMMNH P-16377, Fig 6A–6E), *E*. *gaudrianus* (AMNH 4029, Fig 6F–6N), *Oxyclaenus cuspidatus* (NMMNH P-22034, Fig 6O–6S), *E*. *coryphaeus* (AMNH 764, Fig 7A–7E), *Tri*. *quivirensis* (AMNH 16559, Fig 7F–7I), *Ar*. *primaevus* (MNHN.F.CR17 and CR16, figured in [63]), *Ar*. *mumak* (YPM-PU 18703, figured in [64]), *Periptychus carinidens* (NMMNH P-47693, figured in [62]), *D*. *navajovius* (AMNH 3359, figured in [65]),*“D*.*” europaeus* (MNHN BR 12547, figured in [66]), *Hyaenodictis raslanloubatieri* (MHNT.PAL.2019.1.6, figured in [16]), *Si*. *jiashanensis* (IVPP 10760, figured in [65]), *Pachyaena ossifraga* (AMNH 4262, figured in [67]) and *Maelestes gobiensis* (PSS-MAE 607, figured in [68]). Extant taxa comparisons include *Meles meles* (NMS.Z.RL111.97, S1 Fig), *Gulo gulo* (NMS.Z.GH56.18, S1 Fig), *Orycteropus afer* (NMS.Z.2011.140.1, S1 Fig) and *Tremarctos ornatus* (NMS.Z.2015.19, S1 Fig).

**Fig 8:**
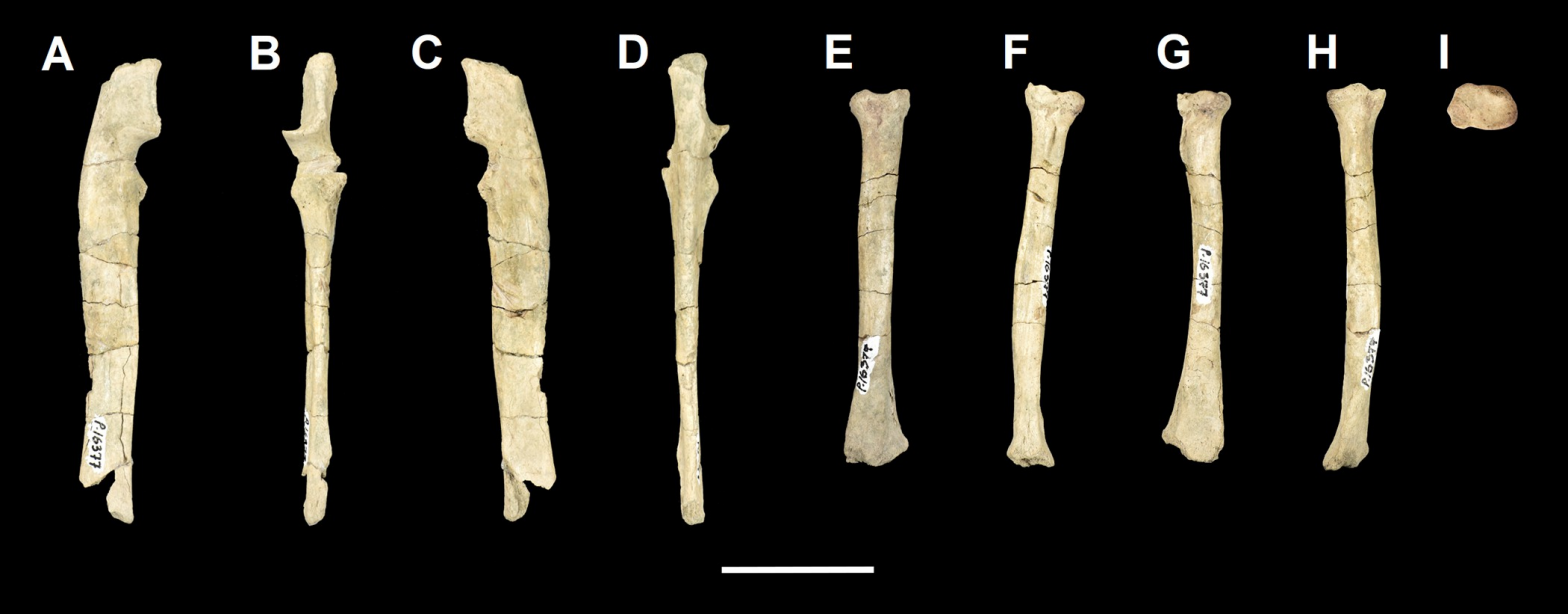
Ulna and radius of *Goniacodon levisanus* (NMMNH P-16377). Right ulna (A-D): (A) lateral view; (B) anterior view; (C) medial view; (D) posterior view. Left radius (E-I): (E) anterior view; (F) lateral view; (G) posterior view; (H) medial view; (I) proximal view. Scale bar: 30 mm.

The ulna of *Tri*. *crassicuspis* was compared to the following extinct taxa: *Go*. *levisanus* (NMMNH P-16377, Fig 8A–8D), *Ar*. *primaevus* (MNHN.F.CR19, CR18, figured in [63]), *Ar*. *mumak* (YPM-PU 18703, figured in [64]), “*D*.*” europaeus* (MNHN BR 12600, figured in [66]), *D*. *navajovius* (AMNH 3359, figured in [65]), *Pa*. *ossifraga* (AMNH 4262, figured in [67]) *H*. *raslanloubatieri* (MHNT.PAL.2019.1.7, figured in [16]) and *Pe*. *carinidens* (NMMNH P-53998, figured in [62]). Extant taxa that we made comparisons to are *Me*. *meles* (NMS.Z.RL111.97, S2 Fig), *Gu*. *gulo* (NMS.Z.GH56.18, S2 Fig), *Or*. *afer* (NMS.Z.2011.140.1, S2 Fig) and *Tre*. *ornatus* (NMS.Z.2015.19, S2 Fig).

The radius of *Tri*. *crassicuspis* was compared to the following extinct taxa: *Go*. *levisanus* (NMMNH P-16377, Fig 7E–7I), *Ar*. *mumak* (YPM-PU 18703, figured in [64]), *Ar*. *primaevus* (MNHN.F.CR20, figured in [63]), *Si*. *jiashanensis* (IVPP 10760, figured in [65]), *Pa*. *ossifraga* (USGS 25292, figured in [67]), *An*. *saurognathus* (AMNH 777, figured in [65]), *D*. *navajovius* (AMNH 3359, figured in [65]), *"D*.*" praenuntius* (UM 69305, figured in [65]), *"D*.*" europaeus* (MNHN BR 12547, figured in [66]) and *Pe*. *carinidens* (NMMNH P-47693, figured in [62]). Extant taxa that we made comparisons to are *Me*. *meles* (NMS.Z.RL111.97, S3 Fig), *Gu*. *gulo* (NMS.Z.GH56.18, S3 Fig), *Or*. *afer* (NMS.Z.2011.140.1, S3 Fig) and *Tre*. *ornatus* (NMS.Z.2015.19, S3 Fig).

Lastly, we compared the femur of *Tri*. *crassicuspis* to that of *Ar*. *mumak* (YPM-PU 18703, figured in [64]), *Ar*. *primaevus* (MNHN.F.CR17, CR16, figured in [63]) and *Pe*. *carinidens* (NMMNH P-19430, figured in [62]).

## Results

### Systematic paleontology

MAMMALIA Linnaeus 1758 [81]

EUTHERIA Gill 1872 [82]

“CONDYLARTHRA” Cope 1881 [83]

“TRIISODONTIDAE” Scott 1892 [24]

### Original diagnosis

“The superior molars are trabecular rather than trenchant, the inferior molars have an absent/rudimentary metaconid, the talon is trenchant and lacking an entoconid, and the astragalus exhibits deep grooves” Scott, 1892 [24]

*Triisodon* Cope 1881 [25]

### Differential diagnosis

*Triisodon* differs from *Eoconodon*, *Goniacodon* and *Oxyclaenus* in the following autapomorphic characters among “triisodontids”: relatively larger canines; a larger paracone relative to the metacone on M1-2; a thin transverse ridge present above the postcingulum on M1-3; M2 is the largest molar; a much higher trigonid relative to the talonid on m1-3; a massively expanded protoconid on m1-3; a relatively more reduced and mesially projecting paraconid on m1-3; a larger and relatively deeper mandible; and a more robust forelimb with relatively better developed muscle attachment sites. *Triisodon* can be further distinguished from *Eoconodon* and *Goniacodon* by the following apomorphic characters: hypocone always present on M1-2; a M2 hypocone that forms a small and distinct circular cusp; shallower stylar shelves on the upper postcanine dentition; a dorsoventrally deeper postglenoid process; and a less prominent sagittal crest. In *Triisodon*, the M3/m3 is highly reduced and the relative size of m3 is proportionately less reduced than in *Goniacodon*, but more so than in *Eoconodon*. Further apomorphic features that distinguish *Triisodon* from *Eoconodon* are: a greater difference between the height of the protoconid and hypoconid than the height of the hypoconid above the tooth base on m1-3; m1-3 entocristid extends mesiolingually on the distolingual base of the metaconid to the lingual cingulid; the base of m1-2 is oriented subhorizontally; m3 subequal in mesiodistal length to m2; m3 talonid is relatively narrower buccolingually; a more well-defined bicipital groove on the humerus. *Triisodon* is further distinguished from *Goniacodon* by having the following apomorphic characters: mesostyle absent on M1-3; M1 reduced in size relative to M2; a single parastylar cusp mesial to the paracone on M2; a M3 parastylar lobe projecting mesiobuccally to near the buccal edge of M2; a p5 protoconid exhibiting a prominent distally directed postprotocristid; p5 paraconid present; larger buccal hypoconid on m1-3; a less basin-shaped talonid on m1-3; a more elongated talonid on m1-3; a deeper masseteric fossa on the mandible; a less prominent deltopectoral crest on the humerus. *Triisodon* is further distinguished from *Oxyclaenus* by the following combination of plesiomorphic characters: ovoid canines lacking distal carina and possessing smooth posterior carina; P5 parastylar lobe is buccolingually constricted so that buccal and lingual borders are subparallel; a significantly more prominent supinator ridge on the humerus.

*Triisodon crassicuspis* Cope 1882 [21]

### Differential diagnosis

Differs from *Tri*. *quivirensis* in the following characters: being ~20% smaller; P4 is anteroposteriorly longer than in *Tri*. *quivirensis*; the molar protoconid is relatively more expanded buccolingually than in *Tri*. *quivirensis*; m3 paraconid and metaconid are not as reduced as in *Tri*. *quivirensis*; the m3 talonid is more elongate with a distinct but proportionally smaller hypoconid than *Tri*. *quivirensis*; in *Tri*. *crassicuspis*, the molars lack a lingual cingulid, whereas it is present in *Tri*. *quivirensis* and particularly well-developed in the position of the metaconid; in *Tri*. *crassicuspis*, the metaconid is subequal to the protoconid in size, whereas it is proportionately smaller in *Tri*. *quivirensis*; in *Tri*. *crassicuspis*, a m1-3 postprotocristid is absent, but is present in *Tri*. *quivirensis*; in *Tri*. *crassicuspis*, the m1-3 cristid obliqua is oriented mesiolingually, whereas in *Tri*. *quivirensis*, it is oriented mesiodistally.

### Etymology

While the etymology of the generic name *“Triisodon"* is not explicitly explained by Cope, the word appears to be derived from the ancient Greek words *tris* (three) and *isos* (equal), and the modern Greek word *donti* (tooth/teeth), presumably referring to lower cheek teeth that are essentially tritubercular (i.e. three-cusped), a term that Cope subsequently coined in 1883 [84].

Again, Cope does not explain the etymology of the species name *“crassicuspis”*, although it appears to stem from the Latin words *crassus* (solid/thick) and *cuspis* (the pointed end of a structure, concerning tooth cusps), with reference to lower molars bearing inflated cusps.

### Body mass

The body mass of *Tri*. *crassicuspis*, based on the specimen NMMNH P-72096 and using a range of dental-based equations that are derived from various measurements of the teeth [77–79], along with the humerus only estimate by Campione and Evans [80], yield a range of results (Table 4). The dental equations generally predict a body mass in the range of 32–44 kg, whereas the postcranial estimate is considerably higher at 66.22 kg. As we argue below, the Legendre [78] all-mammal equation may be the most suitable for *Triisodon* spp., and thus we consider its result of ca. 32 kg body mass to be most reasonable.

**Table 4:** Table showing the body mass estimations for *Triisodon crassicuspis* (NMMNH P-72096) based on various regression equations.

Regression+Reference	Equation	Body mass (kg)
Large mammals (m1 area) (Legendre [77])	LN BM = 1.538(LN m1 area)+3.115	34.52
Mammalia (m1 area) (Legendre [78])	LN BM = 1.7054(LN m1 area)+2.2470	32.19
Non selenodont (m1-3 length (Damuth [79])	logBM = 3.03(logm1-3 length)-0.39	43.45
Long bone minimum circumference based on humerus (Campione and Evans [80])	logBM = 2.6938(log10(HC))-0. 1655	66.22

Abbreviations: BM, Body Mass; HC, Humeral Circumference.

### Description of the dentition of *Triisodon crassicuspis*

NMMNH P-72096 includes associated dentition consisting of four isolated canines, most of the upper cheek teeth in the maxilla (left P2-M3, right P4, right M1-3) and portions of the lower cheek teeth (left partial p2 and partial m3, and right partial m1, partial m2, and m3). Although they are missing from the fossil record (or at least the specimen investigated here and the published record), it is possible that *Triisodon* had a total of 12 incisors (3 on each hemi-mandible and 6 upper incisors), since this is the condition for all eutherians unless secondarily reduced [85]. *Tri*. *crassicuspis* therefore likely possessed 44 permanent teeth, with a dental formula of 3?.1.4.3/3?.1.4.3. Although other “triisodontids” including *Goniacodon levisanus* (AMNH 7798, Fig 3F) and *Eoconodon coryphaeus* (AMNH 16329, figured in Kondrashov and Lucas [17]) possess a P1/p1, the fossils of *Triisodon* do not currently allow to confirm their presence.

### Canines

**Fig 9:**
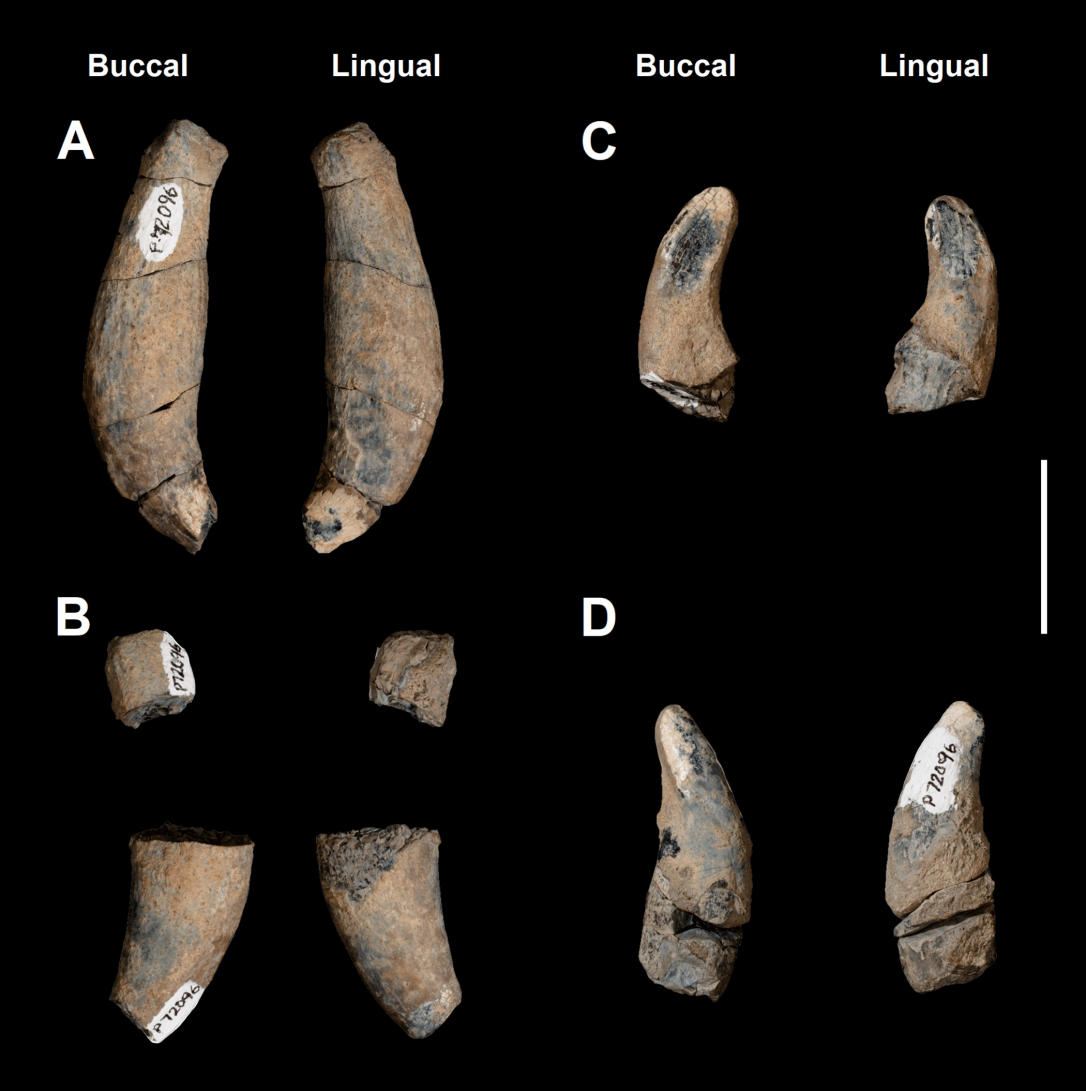
Canines of *Triisodon crassicuspis* (NMMNH P-72096). (A) left upper canine; (B) right upper canine; (C) left lower canine; (D) right lower canine. Scale bar: 30 mm.

The canines of *Tri*. *crassicuspis* are single-rooted teeth that lack mesial and distal carinae (Fig 9). The upper canines are also disproportionately large and considerably longer in length apicobasally relative to the rest of the postcanine dentition. The canine surface texture is smooth. The canines are similar in overall morphology to those of *Tri*. *quivirensis*.

The left upper canine is more complete than the right upper canine and both are missing the apices. The right upper canine is preserved in two pieces, one of the crown and one of the proximal-most portion of the root. The upper canines are robust and generally ovoid, with the lingual surface being slightly flattened for occlusion with the lower canine. This flattening is possibly due to the presence of a wear facet. The crown is significantly shorter than the root, and distal curvature of the upper canine becomes more pronounced towards the crown such that the crown emerges nearly vertically downwards from a more horizontally-lying root.

The lower canines exhibit less pronounced curvature relative to the upper canines, and are smaller in size. The roots are only partially preserved. The lower canines are curved to point distally, with broadly rounded apices that may be a result of wear. They have a flattened buccal surface for occlusion with the upper canines.

### Premolars

**Fig 10:**
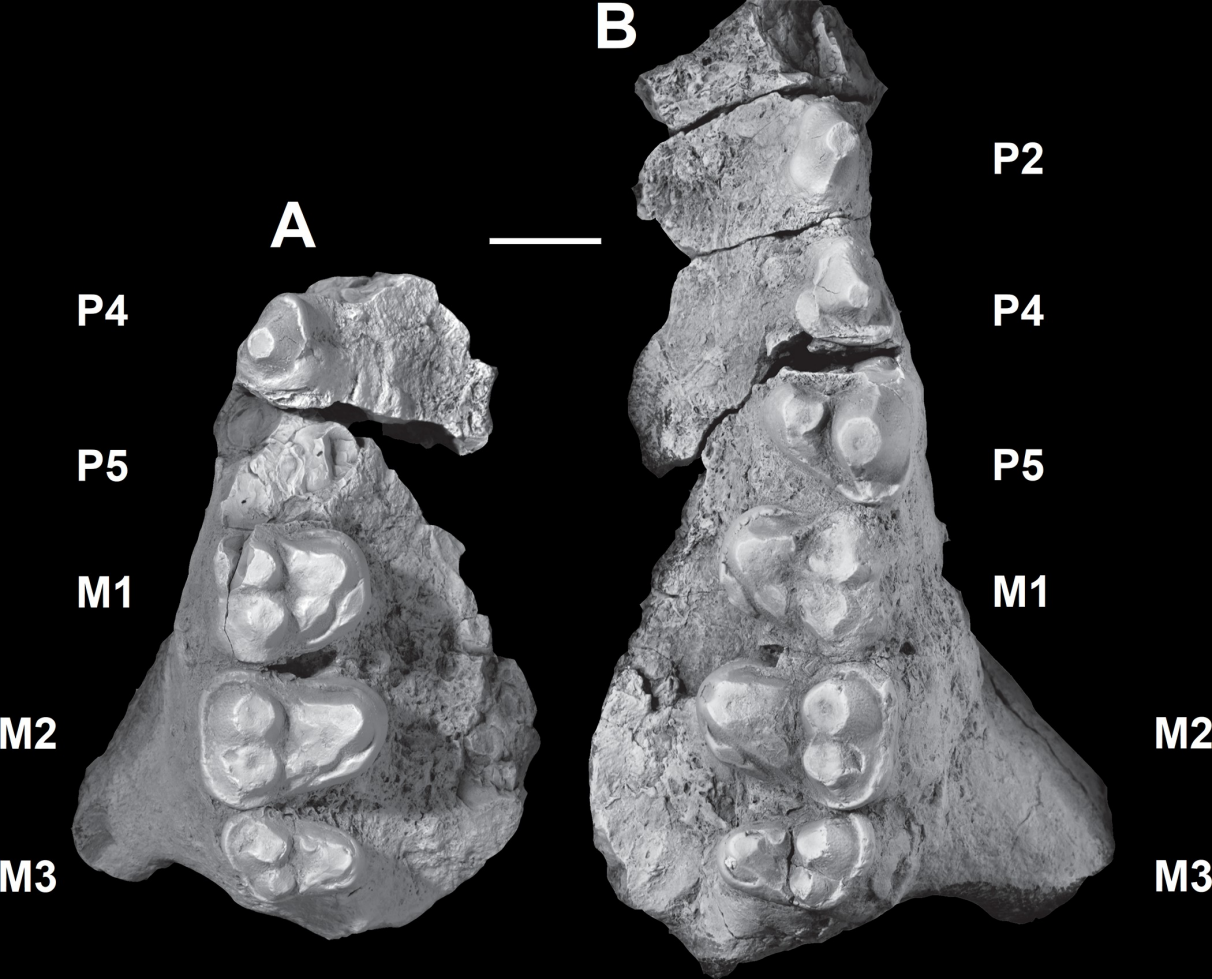
The upper dentition of *Triisodon crassicuspis* (NMMNH P-72096) in occlusal view. Scale bar: 10 mm.

**Fig 11:**
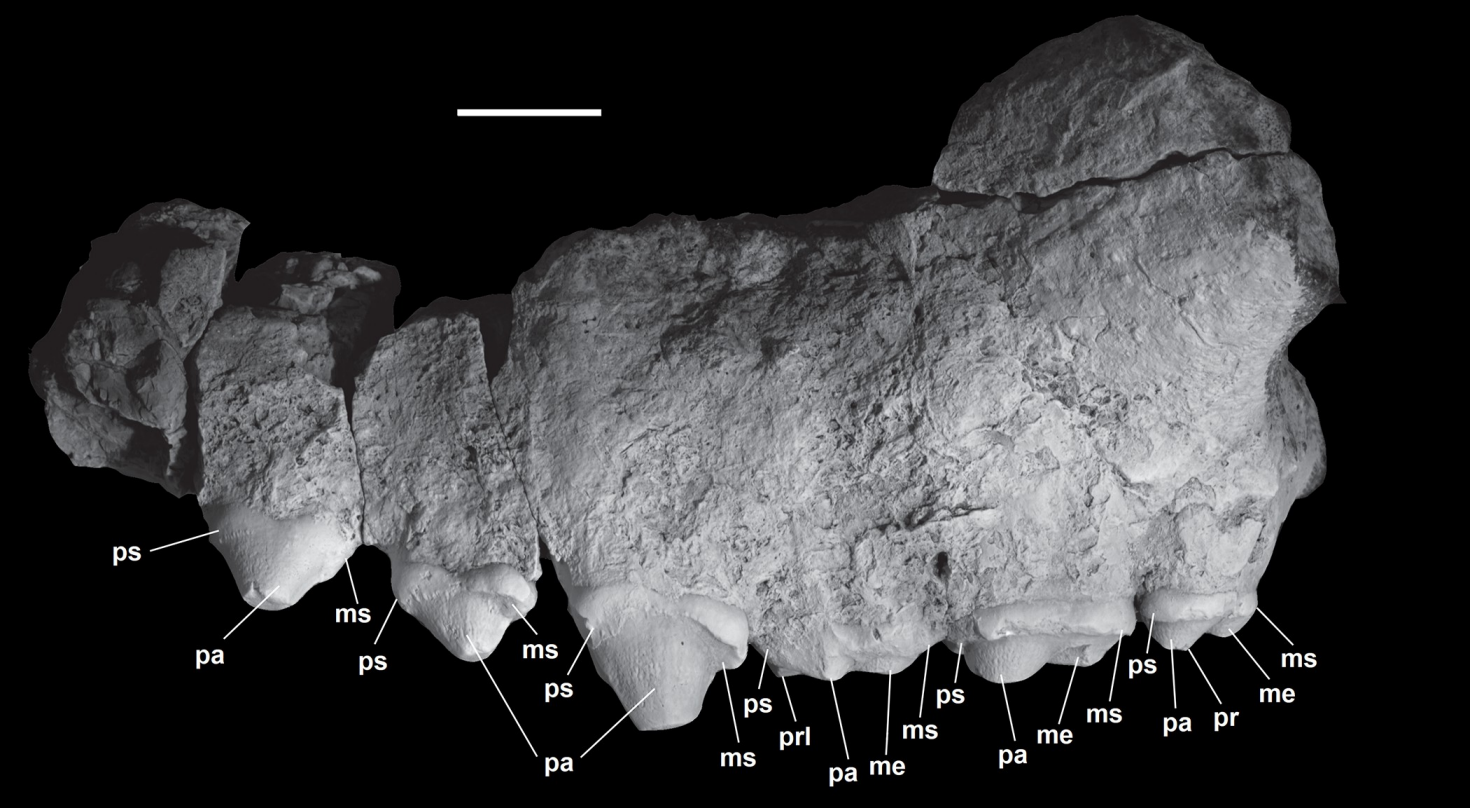
Upper dentition of *Triisodon crassicuspis* (NMMNH P-72096) with left P2-M3 in buccal view. Abbreviations: Me, metacone; ms, metastyle; pa, paracone; pr, protocone; prl, paraconule; ps, parastyle. Scale bar: 10 mm.

**Fig 12:**
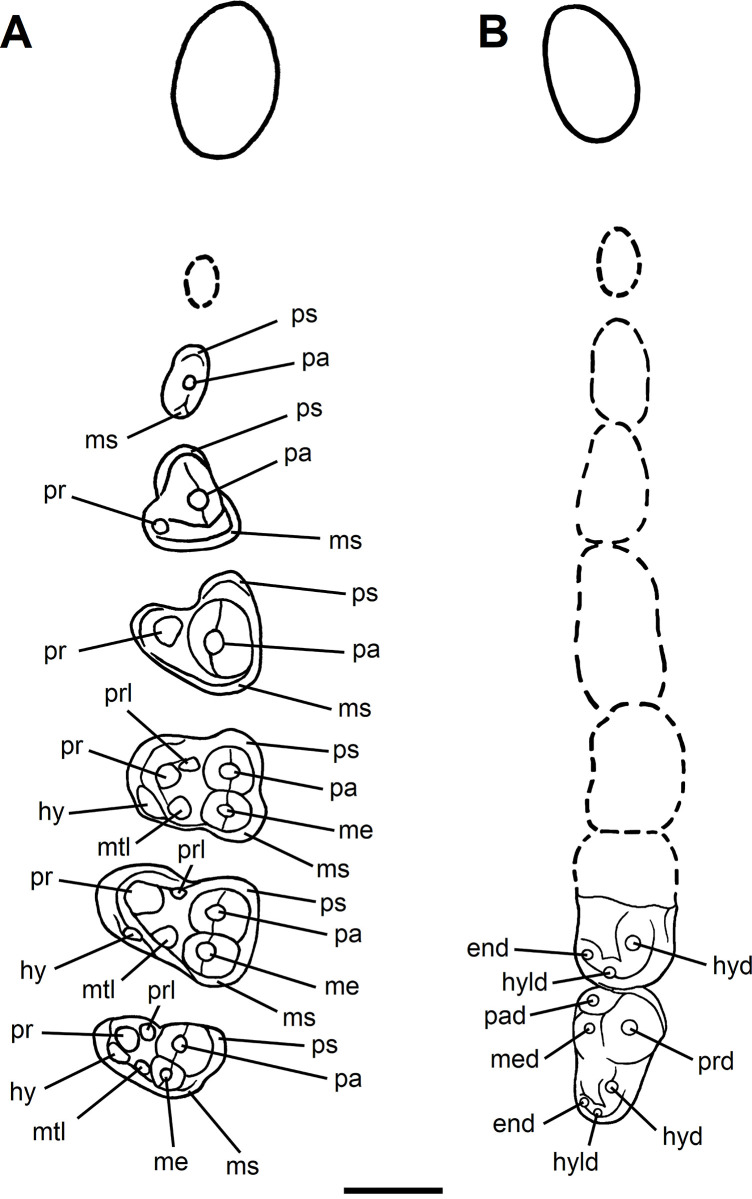
Annotated diagram of the dentition of *Tri*. *crassicuspis* in occlusal view. (A) Diagram of the left dentition of *Tri*. *crassicuspis* in sequence. (B) Diagram of the lower right dentition of *Tri*. *crassicuspis* in sequence. (The left P1, right p1, right p2-m1 and the trigonid of the right m2 are represented by a dotted outline as they are speculative). The teeth represented by dotted outlines are based on observation of the “triisodontids” *Triisodon quivirensis* (AMNH 3352, figured in [17]), *Eoconodon coryphaeus* (AMNH 16329, figured in [17]) and *Goniacodon levisanus* (AMNH 7798, Fig 3F). Abbreviations: End, entoconid; hy, hypocone; hyd, hypoconid; hyld, hypoconulid; me, metacone; med, metaconid; ms, metastyle; mtl, metaconule; pa, paracone; pad, paraconid; pr, protocone; prd, protoconid; prl, paraconule; ps, parastyle. Scale bar: 10 mm.

**Fig 13:**
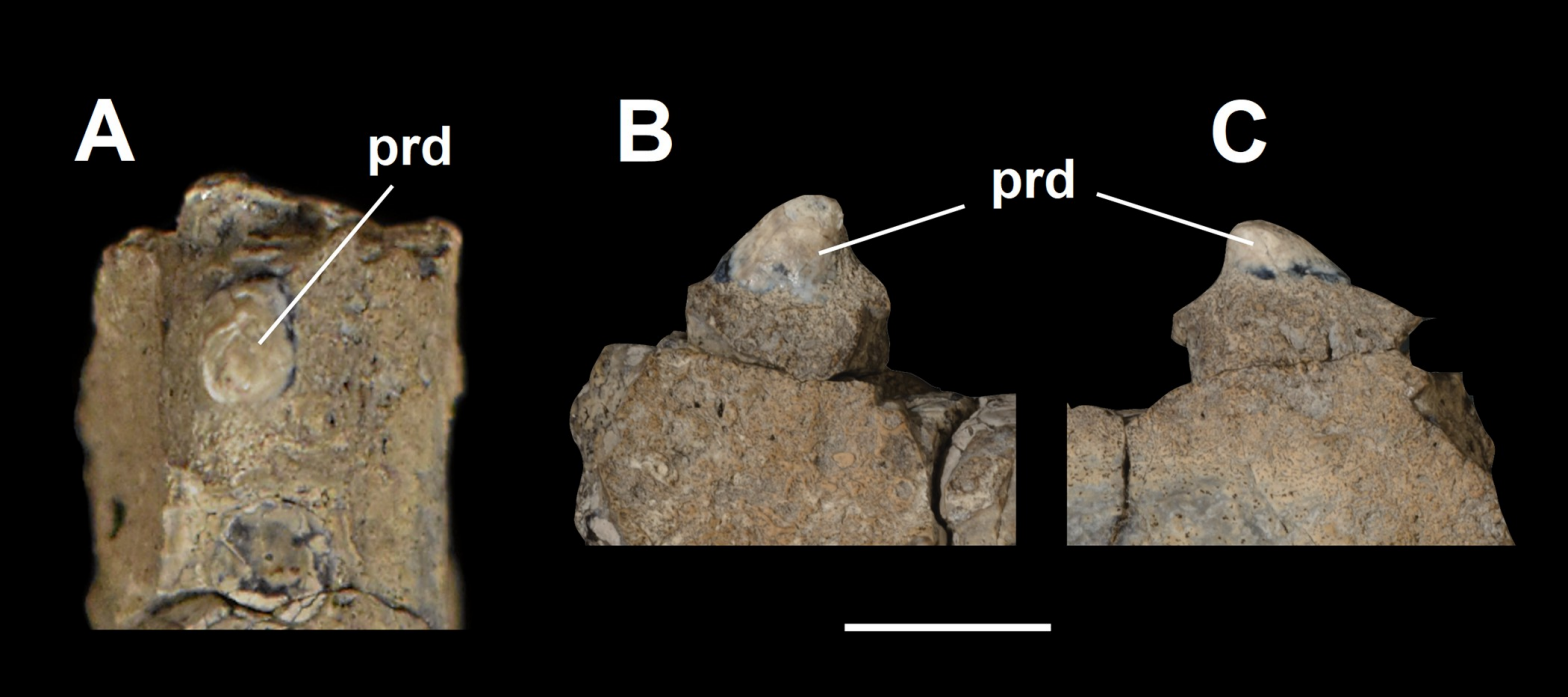
Partial left p2 of *Tri*. *crassicuspis* (NMMNH P-72096). (A) occlusal view; (B) buccal view; (C) lingual view. Abbreviations: prd, protoconid. Scale bar: 10 mm.

The second premolar is distinctly simpler in morphology relative to the more posterior premolars. Thus, the description starts with the second premolar (P2/p2), then a description of the overall morphology of the successive premolars (P4/p4, P5/p5). For NMMNH P-72096, the description of the upper premolars is based on the left P2-5 (Figs 10–12), the description of the lower premolars is based on the left p2 (Fig 13).

### Upper premolars

All the upper premolars are triangular shaped in buccal or lingual views and have well-developed mesiodistally aligned shearing surfaces on the paracones formed by cristae (pre- and postparacristae). The upper premolars are considerably taller apicobasally than the molars. The upper premolars of *Tri*. *crassicuspis* increase in size distally along the tooth row. A conical, centrally positioned paracone dominates the crown of P2 and P4.

P2 possesses two roots and is mesiodistally elongate. It bears only a large and buccolingually compressed paracone, with the apex being distally directed. The crown of the tooth, at the level of the paracone, appears to lack a lingual expansion and a protocone. The cingulae are poorly developed. The parastyle at the mesial end of the crown and the metastyle at the distal end are low and indistinct.

P4 possesses three roots (with two buccal roots and one supporting the protocone). P4 is more molariform than P2. As with P2, the paracone is prominent, distally directed and bears distinct pre- and postparacristae. The preparacrista extends mesially to a low, mesially convex parastylar lobe at the mesial edge of P4, in line with the paracone. The crown of P4 exhibits a lingual expansion at the base of the paracone, which bears a highly reduced protocone. From the distal end of the metastyle, the buccal cingulum extends mesiobuccally around the paracone, whereas the postcingulum extends lingually to the lingual edge of the protocone.

P5 also possesses three roots. P5 is the most molariform and largest of the upper premolars. It has a well-developed, mesiobuccally positioned paracone, that is highly distally directed. The mesial end of the tooth bears a small parastyle which exhibits some damage, whereas the distal end of P5 exhibits a more prominent metastyle. The parastylar lobe is broadly rounded mesially and there is no evidence for a paraconule. Both a metacone and metaconule are absent. A distinct protocone with a sharp preprotocrista is positioned lingual to the paracone. P5 has a precingulum, and a postcingulum situated distal to the metacone, ending buccal to the level of the protocone. There is a low ectocingulum running along the buccal margin of the tooth, from the metastylar lobe to the base of the paracone.

In NMMNH P-72096, there is a 3.8 mm diastema between left P2 and P4, while a 4.7 mm diastema is present between left P4 and P5.

#### Lower premolars

The proportional increase in successive premolar size for *Tri*. *crassicuspis* (and also *Tri*. *quivirensis*) is less pronounced relative to that observed in *Go*. *levisanus* (AMNH 7798). In NMMNH P-72096, the p2 is a relatively small, double-rooted tooth, with no talonid preserved due to damage (Fig 13A–13C). The p2 is mesiodistally elongate. The protoconid is prominent and distally directed. The mesial end of the tooth appears to lack a paraconid. In NMMNH P-72096, there appears to be no diastema between p2 and p4, or p4 and p5.

#### Molars

*Upper molars*. The description of the upper molars of NMMNH P-72096 is based on the left M1-3 (Figs 10 and 11). The upper molars are tribosphenic, all three which are implanted by three roots: two subequal and small buccal roots and a larger root above the protocone. The upper molars are bunodont and the trigon exceeds the talon in width. The enamel surrounding the upper molars is finely crenulated. In NMMNH P-72096, M1 is smaller in size relative to M2, whereas in *Goniacodon* spp. [13], M1 is typically larger or equal in size to M2. The upper molar stylar shelves are less well-developed than those of *Goniacodon* spp. As in *Tri*. *quivirensis* (NMMNH P-20918, Fig 3A–3E), the M1-2 paracone of *Tri*. *crassicuspis* is somewhat larger than the metacone. The M1-2 parastylar lobe of *Tri*. *crassicuspis* is buccally expanded, forming a convex buccal edge, and is more expanded than the metastylar lobe. The paracone and metacone of M1-3 are close so that the bases unite. The paracone, metacone and protocone of M1-3 are connate in cross-section. All the upper molars of *Tri*. *crassicuspis* lack a mesostyle and have well-developed hypocones. In *Tri*. *crassicuspis*, the protocone is the largest molar cusp and is positioned on the mesiolingual border of the tooth, lingual to and between the paracone and metacone. In *Tri*. *crassicuspis*, the ectoflexus on the buccal margin of M1-2 is weakly pronounced.

M1 is tribosphenic and approximately quadrate in occlusal profile. The paracone and metacone are conical. A prominent ectocingulum runs along the buccal margin of the tooth. At the mesiobuccal corner, a slight expansion of the stylar shelf exhibits a parastylar lobe. At the distobuccal corner of the metacone is a metastylar lobe, more salient and with a more rounded margin than that of M2. The paraconule and metaconule are well-developed and situated lingual to the paracone and metacone, respectively. The metaconule is larger than the paraconule. The protocone is roughly equivalent in height to the paracone. The pre- and postprotocrista extend buccally from the lingual surface of the protocone to connect to the paraconule and metaconule, respectively. At the distolingual margin of the tooth, slightly distal to the protocone, is a relatively large hypocone located on an expansion of the postcingulum. A basal endocingulum is present on M1.

M2 is more triangular in occlusal profile than M1. The crown of M2 has a highly distinctive outline, with a straight distal margin that is approximately perpendicular to the straight buccal margin. M2 exhibits a parastylar lobe (slightly larger than that of M1) mesiobuccal to the paracone. A preparaconule crista extends towards the parastylar lobe, whereas the postmetaconule crista extends towards the distal buccal corner of the ectocingulum. The metastylar lobe is transversely broader than that of M1, and the hypocone at the distolingual corner of M2 is reduced relative to that of M1. The protocone is slightly taller than the paracone. As in M1, a prominent ectocingulum is present along the buccal margin of M2.

The M3 is almost oval shaped in occlusal aspect and is the smallest of the upper molars. The parastylar lobe of M3 projects mesiobuccally to near the buccal edge of M2. The paracone is larger than the metacone, and there is a shallow ectoflexus between them on the buccal margin of the tooth. The parastylar lobe and metastylar lobe are not greatly expanded, and are buccally surrounded by a broad ectocingulum. M3 bears a paraconule and a highly reduced metaconule, both conules bear distinct cristae. M3 has a large protocone that is surrounded by a broad postcingulum extending around its distolingual side. As in M1, the protocone is approximately equal in height to the paracone. The hypocone is compressed.

**Fig 14:**
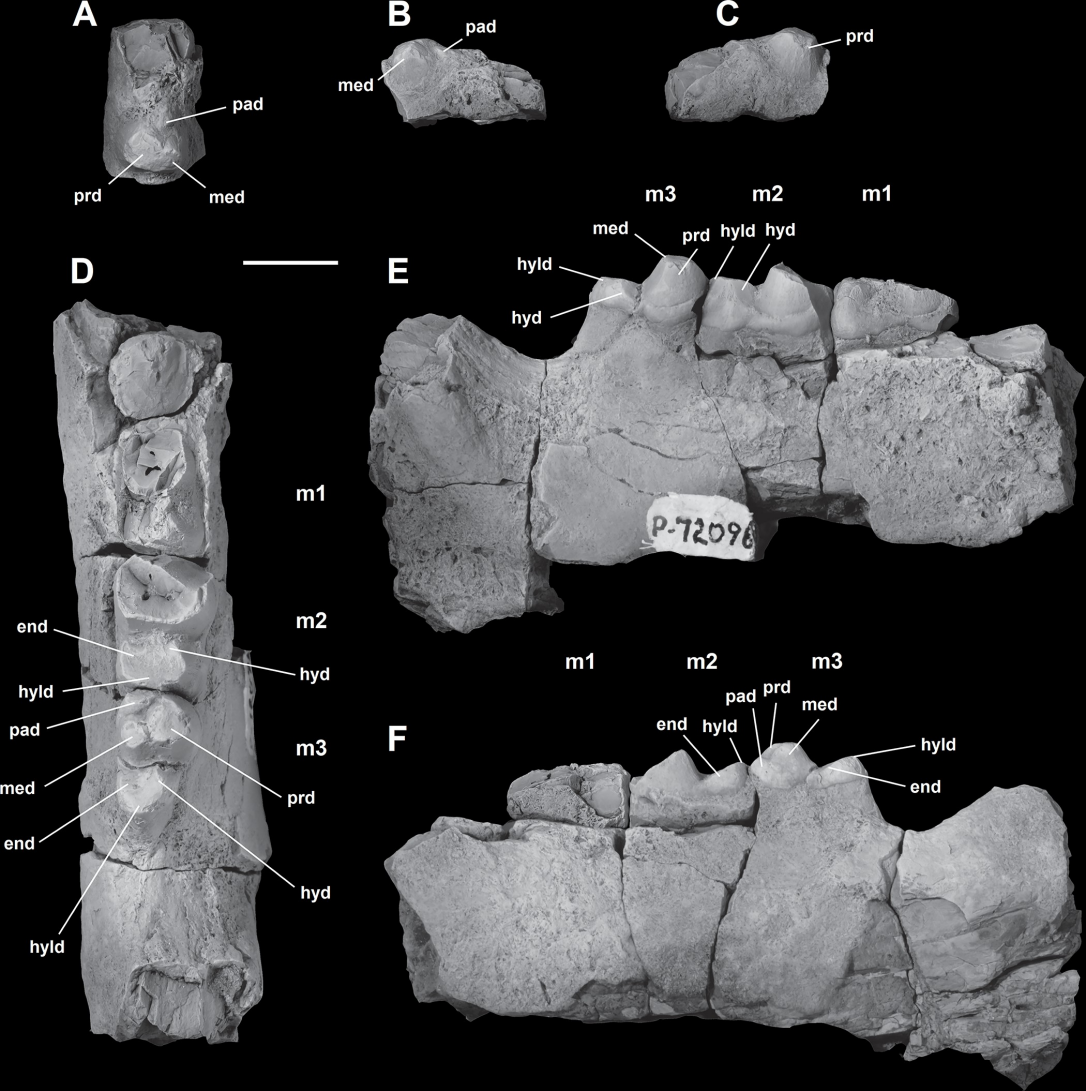
Lower molars of *Triisodon crassicuspis* (NMMNH P-72096). Partial left m3 (A-C): (A) occlusal view; (B) buccal view; (C) lingual view. (D) Partial right m1 and m2, and m3, in occlusal view. (E) Partial right m1 and m2, and m3, in buccal view. (F) Partial right m1 and m2, and m3, in lingual view. Abbreviations: End, entoconid; hyd, hypoconid; hyld, hypoconulid; pad, paraconid; prd, protoconid; med, metaconid. Scale bar: 10 mm.

**Fig 15:**
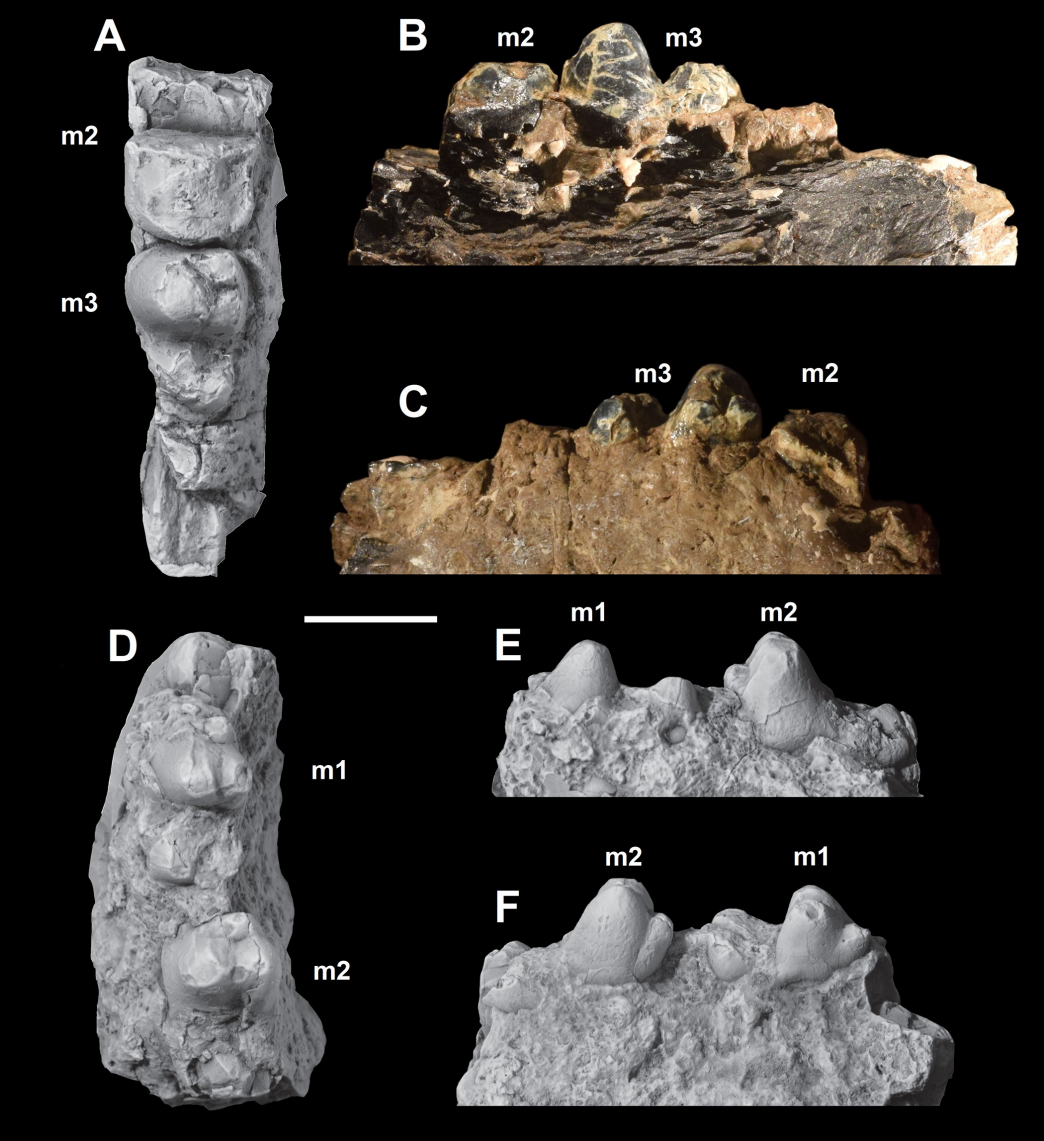
Lectotypes of *Tri*. *crassicuspis* and *Tri*. *rusticus*. The lectotype of *Triisodon crassicuspis*, AMNH 3178, a left partial dentary with a partial m2 and a complete m3, in occlusal (A), buccal (B), and lingual (C) views. The lectotype of *Tri*. *rusticus*, AMNH 3225, a left partial dentary with partial m1-2, in occlusal (D), buccal (E), and lingual (F) views. Scale bar: 10 mm.

*Lower molars*. The description of the lower molars of NMMNH P-72096 is based on the right m1-3 (Fig 14D–14F). The preserved lower molars of NMMNH P-72096 are essentially identical to those of two *Triisodon* specimens named by Cope in 1882 [21] and in 1883 [22] respectively. The lectotype AMNH 3178 *Tri*. *crassicuspis* [21] preserves a m3 and a partial m2 (Fig 15A–15C) and AMNH 3225 *Tri*. *crassicuspis*, the lectotype of *Tri*. *rusticus* [22], preserves a partial m1-2 (Fig 15D–15F). In NMMNH P-72096, the left m3 is isolated and heavily worn, preserving only the trigonid (Fig 14A–14C), and the trigonid and talonid of the right m1, along with the trigonid of the right m2, is broken. A mesial trigonid and a distal talonid form the lower molars. *Tri*. *crassicuspis* possesses a trigonid that is considerably taller than the talonid, an important “triisodontid” feature (e.g. [11]). In NMMNH P-72096, the lower molars are individually supported by two roots. A sharp cingulid runs along the buccal edge of the lower molars, whereas a lingual cingulid appears to be absent. The lower molars appear to be closely associated, with no diastema between the teeth, each which is implanted by two roots.

The crown of m1 is approximately quadrate in occlusal profile (Fig 14D). In occlusal aspect, m2 is more quadrate relative to m1, and consists of a basin-shaped talonid bordered by a crista that exhibits a large buccal hypoconid, a small distal hypoconulid, and a small entoconid mesiolingual to the hypoconulid.

The smallest of the lower molars is m3, the trigonid/talonid height for that tooth which is = 1.9. m3 is distally tapering, with the talonid being buccolingually narrower than the trigonid. A reduced paraconid is located mesiolingually at the mesial end of m3. The m3 has a tall metaconid and protoconid, both cusps which are united along most of their height. The m3 is comparable in morphology to m2 in possessing a basin-shaped talonid with a crista bearing a hypoconid, hypoconulid and entoconid. On m3, the entoconid is larger than the hypoconulid. In m2, both cusps are united along most of their height. m3 is comparable in morphology to m2 in possessing a basin-shaped talonid with a crista bearing a hypoconid, hypoconulid and entoconid. On m3, the entoconid is larger than the hypoconulid.

The m3 morphology of NMMNH P-72096 is nearly identical to that of AMNH 3178. The many shared characters include an erect paraconid. The paraconid is apicobasally shorter than the metaconid and protoconid, and situated on the mesiolingual margin of the trigonid. The trigonid is dominated by the protoconid, which is the largest cusp on m3. The metaconid and protoconid of m3 are inflated and confluent at the base above the trigonid basin, or “twinned”. The metaconid and protoconid are much higher than the talonid cusps, with a greater difference between the height of the protoconid and hypoconid than the height of the hypoconid above the tooth base. As in m2, a large buccal hypoconid dominates the talonid of m3, extending into the center of the talonid. At the distolingual margin of the talonid is a prominent entoconid, and at the distal end is a relatively smaller hypoconulid.

**Fig 16:**
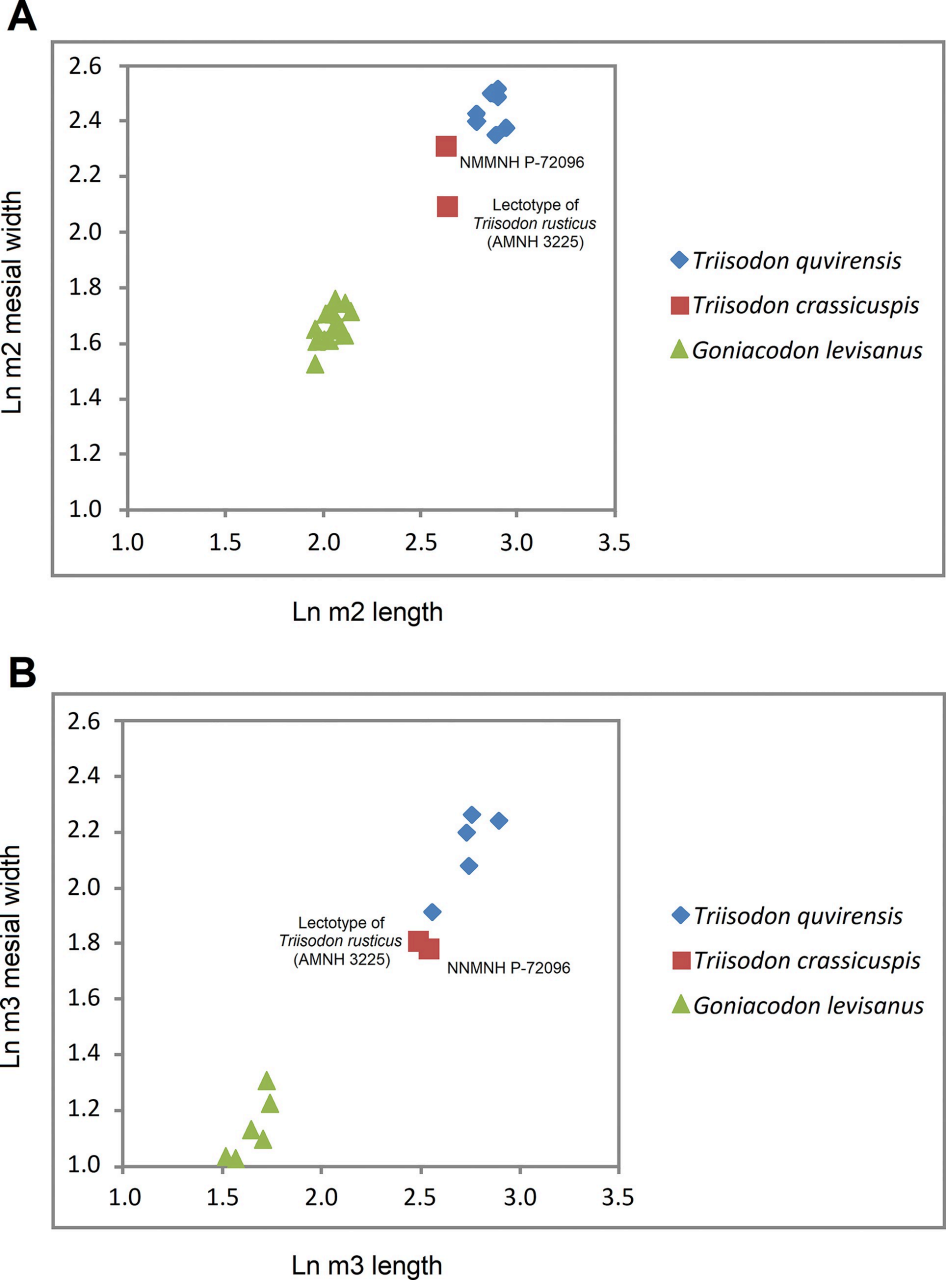
Graphical comparison for the tooth measurements of “triisodontids” (S4 TriisodonLOWER). Bivariate plots of Ln m2 length relative to Ln m2 mesial width (A) and Ln m3 length relative to Ln m3 mesial width (B) of *Triisodon quivirensis*, types of *Tri*. *crassicuspis* and *Tri*. *rusticus*, NMMNH P-72096, and *Goniacodon levisanus*.

The lower molars of *Tri*. *crassicuspis* differ from those of *Tri*. *quivirensis* in being approximately 20% smaller (Fig 16), and in having a less reduced paraconid and metaconid on m3. In *Tri*. *crassicuspis*, the talonids of the lower molars are considerably more elongate than those of *Go*. *levisanus* and *Tri*. *quivirensis*. In addition, the talonids exhibit mesiodistally aligned hypoconulid crests close to the entoconids, as in other “triisodontids" such as *Go*. *levisanus*. The relative size of m3 is proportionately more reduced in *Go*. *levisanus* than in *Tri*. *crassicuspis*.

#### AMNH 3178 and AMNH 3225

*AMNH 3178*. The diagnosis of *Triisodon crassicuspis* was based on AMNH 3178 (Fig 15A–15C) [21]. Our updated diagnosis is featured in the systematic paleontology section. In m2, the trigonid is not preserved. A large buccal hypoconid dominates the talonid, extending into the center of the talonid. Therefore, the talonid is not as basin-shaped to the same extent as the talonid of *Go*. *levisanus*. The m2 seems to exhibit considerable wear and the specific cusp anatomy is hard to determine with certainty. At the distolingual corner of the talonid is a prominent entoconid, and at the distal edge is a relatively smaller hypoconulid.

As observed in NMMNH P-72096, m3 is somewhat more reduced in size relative to m2. In AMNH 3178, the talonid of m3 is slightly less than half the apicobasal length of the trigonid, with a larger difference in apicobasal length compared to that observed in NMMNH P-72096. The distal edge of the talonid seems to bear a reduced hypoconulid, and the occurrence of additional cusps including an entoconid is unable to be determined with certainty due to the lingual surface exhibiting some damage.

The remarkable visual similarities between AMNH 3178 and specimens of *E*. *coryphaeus* is particularly noteworthy, which led Matthew [45] to synonymise both taxa. It is true that AMNH 3178 resembles *E*. *coryphaeus* (AMNH 3177) in various features: the talonid is dominated by the hypoconid, the metaconid and paraconid are of roughly the same size, and the biggest cusp is the protoconid. Furthermore, the preserved m2-3 of AMNH 3178 are of roughly the same size as those of AMNH 3177, although one major diagnostic feature of *Eoconodon* is a trigonid that is comparatively short apicobasally [46,86] that, as evident from m3, contrasts to the condition in AMNH 3178, which possesses a trigonid that is considerably elongated apicobasally.

*AMNH 3225*. This specimen formed the basis of another species, *Tri*. *rusticus*, a supposed synonym of *Tri*. *crassicuspis*. Only descriptions of lower dentition have ever been published for *Tri*. *rusticus*. In AMNH 3225, the trigonids of m1 and m2 are preserved. Very little morphological data can be obtained from the talonid of m1, which is partially obscured by concretion, and that of m2 is broken (Fig 15D–15F). It is from this specimen that the diagnosis of *Tri*. *rusticus* was produced by Cope in 1883 [22]:

“The interior anterior cusp is nearly as elevated as the exterior, and is united with it nearly to the apex; the anterior cusp is a tubercle which projects forwards from its anterior base. The heel of the tooth is wide, and is rounded posteriorly, and supports three tubercles, an external, a posterior and an internal, all in contact with each other. On the second true molar the internal anterior tubercle presents a slightly projecting edge anteriorly and posteriorly, which bounds a shallow vertical groove of the mass which represents their united bodies. This is not apparent in the first. The enamel is smooth, but the animal is rather old.”

Cope likely came to the conclusion that the individual is of old age based on the heavily worn enamel. AMNH 3225 exhibits some diagnostic features of *Tri*. *crassicuspis* that are also present in NMMNH P-72096, including a massively expanded protoconid, a distinctly elongate talonid, a distinct buccal hypoconid and the quadrate outline of m1. Additionally, AMNH 3178, AMNH 3225 and NMMNH P-72096 are similar in size and have very similar preservation, which matches with specimens from the earliest Torrejonian—the same strata and general area from which NMMNH P-72096 was collected. We therefore agree with Matthew [31] that AMNH 3225 belongs to *Tri*. *crassicuspis*.

In both m1 and m2, the metaconid and protoconid are conical. m1 is smaller in size relative to that of AMNH 3178 and NMMNH P-72096. In m1, the paraconid is smaller in size than the metaconid in occlusal aspect. The mesiobuccally projecting paraconid forms an overall profile of mesial tapering relative to the rest of m1. The metaconid and protoconid form the tallest portion of the tooth and are approximately equal in height. The base of the metaconid is smaller than that of the protoconid in occlusal aspect. m1 has a basin-shaped talonid, but approximately half of the buccolingual width is occupied by a large buccal hypoconid. Opposite the hypoconid, on the distolingual corner is an entoconid that is subequal in size to the hypoconid, but is difficult to determine exactly how wide due to concretion. A smaller hypoconulid appears to be preserved on the distal end of m1. As observed in NMMNH P-72096, m2 is longer mesiodistally and buccolingually than m1. As in m1, the paraconid is smaller than the metaconid in occlusal aspect. As in m1, the base of the metaconid in m2 is slightly smaller than the base of the protoconid.

#### Dentition summary

The dentition of *Tri*. *crassicuspis* shows numerous morphological resemblances to those of various other “triisodontids” including, the diagnostic features of that group: a basined talonid on the lower molars and tribosphenic upper molars with a thick cingulum surrounding the crown [11]. In the lower molars, the height of the trigonid is formed from the metaconid and protoconid, the former cusp which is subequal in height, but both cusps are merged along much of their height. The upper canines of *Tri*. *crassicuspis* are massive.

The premolar shearing edges form a secodont-like morphology observed in modern carnivorans. The large P5 would have likely occluded with p5, with the paracone being the largest cusp of the upper premolars/molars. The gap between the protocone and paracone of P5 would have provided a groove into which the p5 protoconid occluded, forming a precise surface on which the p5 shearing edge could move across to slice flesh. In addition, the high height differential between the trigonids and talonids, high cusped, backward projecting protocones on the premolars, and mesiodistally-oriented hypoconulid crests on the lower molars of *Tri*. *crassicuspis* are morphologies that are indicative of effective shearing capabilities.

### Description of the osteology of *Triisodon crassicuspis*

#### Cranium

**Fig 17:**
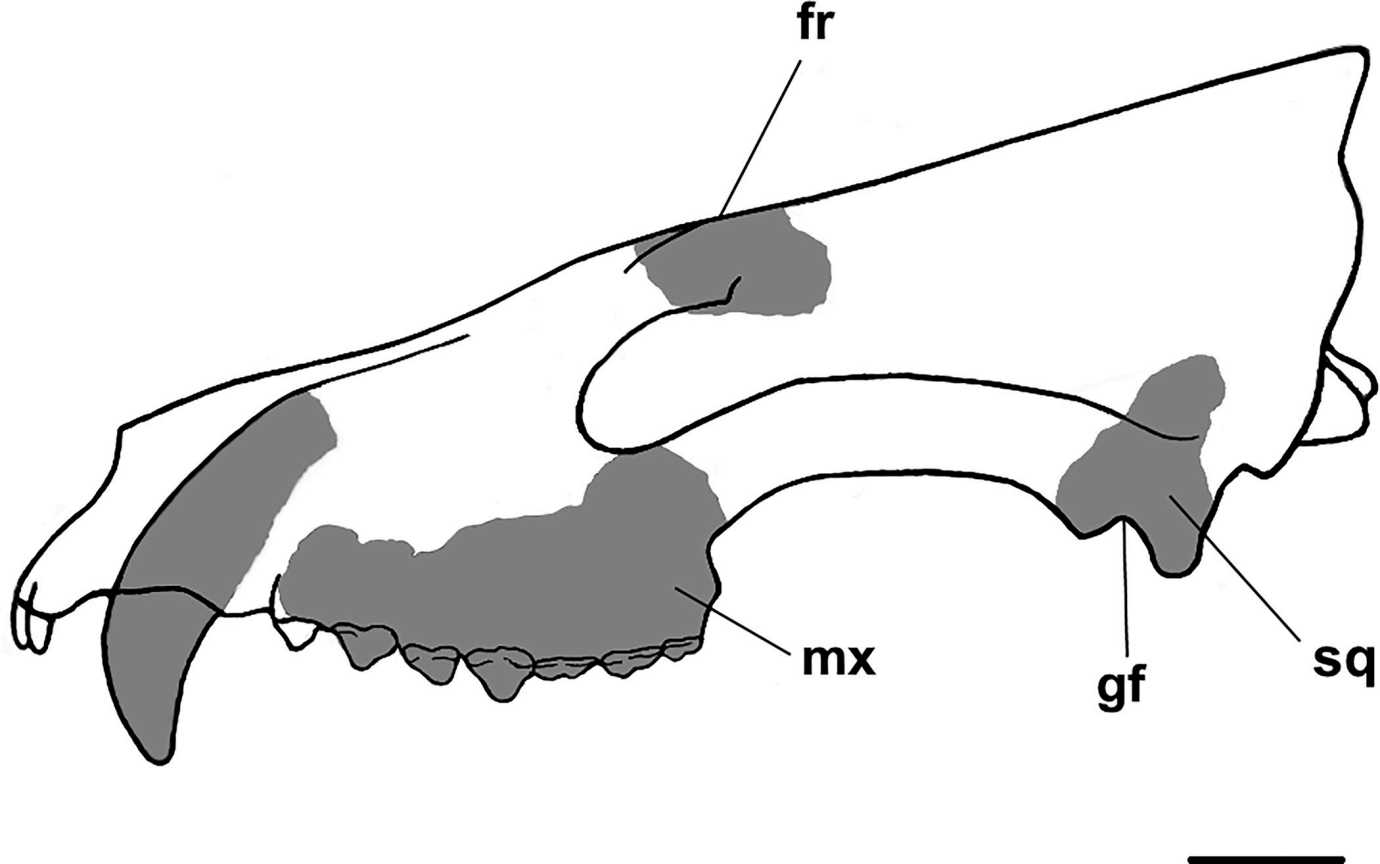
Line drawing restoration of the skull of *Triisodon crassicuspis* in lateral view. Based on NMNH P-72096 and comparisons to *Tri*. *quivirensis* (USNM 22483, Fig 4A–4C), *Eoconodon coryphaeus* (AMNH 16329 [31] and DMNH.EPV.130976 [3]) and mesonychids [65]. Gray areas represent areas for which material is known, white areas represent areas reconstructed hypothetically. Abbreviations: fr, frontal; gf, glenoid fossa; mx, maxilla; sq, squamosal. Scale bar: 10 mm.

**Fig 18:**
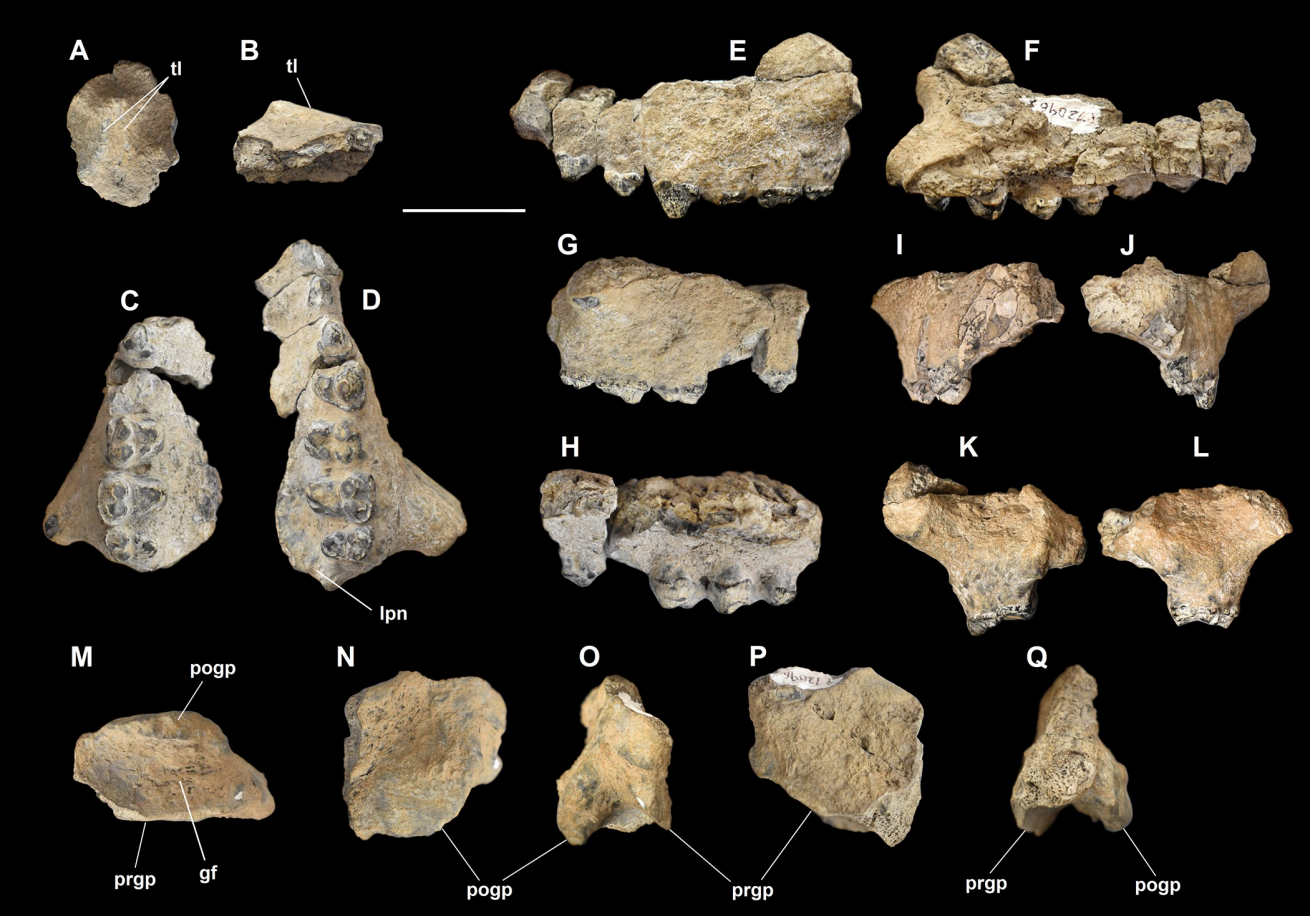
Cranial fragments of *Triisodon crassicuspis* (NMMNH P-72096). Frontal (A-B): (A) dorsal view; (B) lateral view. Maxillae (C-L): (C) right maxilla in ventral view; (D) left maxilla in ventral view; (E) left maxilla in lateral view; (F) left maxilla in medial view; (G) right maxilla in lateral view; (H) right maxilla in medial view; (I) right maxilla in anterior view; (J) left maxilla in anterior view; (K) left maxilla in posterior view; (L) right maxilla in posterior view. Right glenoid fossa (M-Q): (M) ventral view; (N) posterior view; (O) lateral view; (P) anterior view; (Q) medial view. Abbreviations: gf, glenoid fossa; lpn, lesser palatine notch; pogp, postglenoid process; prgp, preglenoid process; tl, temporal lines. Scale bar: 30 mm.

Portions of the skull of *Triisodon quivirensis* have been recovered. The skull of *Tri*. *crassicuspis* is much more poorly represented, with only two unambiguously identified cranial fragments found in association with the maxillary specimens of NMMNH P-72096. These cranial fragments are all part of the braincase, including a fragment preserving the posterior portion of the paired frontal bones that contribute to the sagittal crest, along with a partial squamosal (Figs 17 and 18). Most of the zygomatic arches are missing, with only the anterior roots preserved. All these fragments closely match the skull material of *Tri*. *quivirensis*, USNM 22483.

#### Frontal

The frontal forms much of the skull roof dorsal to the orbit (Fig 18A and 18B). The preserved portion of the frontal consists of the region where the temporal lines converge posteriorly, with a break located anterior to where the sagittal crest would have been in life. In *Tri*. *crassicuspis*, the paired frontal bones appear to form a flat roof (Fig 18B). The temporal lines appear to be more curved than those observed in *Eoconodon coryphaeus* (Fig 18A). The temporal lines of *Tri*. *crassicuspis* form a broadly-rounded ridge as observed in *E*. *coryphaeus* (AMNH 16329, DMNH.EPV.130976) and *Tri*. *quivirensis* (USNM 22483). On the frontal, the temporal lines of *Tri*. *crassicuspis* are broadly spaced, but slightly less so than in *E*. *coryphaeus*, so that they bear more similarity to those of *Tri*. *quivirensis* and *Arctocyon primaevus* (MNHN.F.CR700) (Fig 18A).

#### Maxilla

The lateral walls of the rostrum are formed by the paired maxillae (Fig 18C–18L). The left and right maxillae include the anterior roots of the zygomatic arches, but both are incomplete, missing the anterior margin of the maxillary bone. The maxillae are broken ventral to where the infraorbital foramen (= infraorbital canal) would have been in life. The left maxilla is the more complete of the two specimens (Fig 18D–18F, 18J and 18K). The maxillae are anteroposteriorly elongate.

In *Tri*. *crassicuspis*, the maxillae have slightly convex lateral walls, forming a relatively rounded rostrum in anterior view, and are relatively deep dorsoventrally. The fragmentary nature of the specimen makes observations of the life position of the maxilla relative to the other cranial bones difficult, including the premaxilla anteriorly, the nasal dorsally and the lacrimal. There is marked constriction along the rostrum, constricting above P2 as in *Ar*. *primaevus* (MNHN.F.CR700).

The maxillae contribute to much of the anterior roots of the zygomatic arches. The maxilla extends only slightly onto the zygomatic arch in lateral view. The anterior root of the zygomatic arch has a well-developed ventral surface, where the jugal is limited to comprising a small short process that does not extend far into the maxillary area. The lateral side of the zygomatic arch is formed by the long process of the jugal. The anterior root of the zygomatic arch consists of an anteroposteriorly wide shelf on its ventral surface, extending to the mesial edge of M2. The posterior edge of the anterior zygomatic root is aligned with M3.

Ventrally, the maxillae form the lateral and anterior constituents of the palate (in association with the palatine posteromedially and the premaxilla anteriorly), and accommodate the upper postcanine teeth. Little of the palate remains and this is heavily concreted. There is no clear maxilla-palatine suture preserved anywhere on the specimen. The alveolar processes, which accommodate the teeth, are well-developed dorsoventrally and therefore gives the palate a slightly arched appearance in anterior or posterior aspect.

In *Tri*. *crassicuspis*, the lesser palatine notch, which is located on the posterior edge of the hard palate, posteromedial to the hypocone of the left M3, appears to be shallow and moderately distinct. The lesser palatine notch would have channeled the lesser palatine nerve and accompanying vessels in life [74,76]. Neither a major palatine foramen or an incisive foramen is preserved on the specimen. In ventral aspect, the overall shape of the palate is very similar to that of *Tri*. *quivirensis* (USNM 22483).

#### Squamosal

The lateral portion of the squamosal constitutes the articular area and posterior arm of the zygomatic arch. In NMMNH P-72096, only a fragment of the posterior portion of the right squamosal including the complete glenoid fossa is preserved (Fig 18M–18Q).

In *Tri*. *crassicuspis*, the glenoid fossa was likely situated completely on the squamosal, as in *Tri*. *quivirensis* (USNM 22483), *E*. *coryphaeus* (AMNG 16329, DMNH.EPV.130976), *Goniacodon levisanus* (USNM 15503) and *Ar*. *primaevus* (MNHN.F.CR700). The glenoid fossa of *Tri*. *crassicuspis* is massive and forms a deeply excavated fossa that is longer mediolaterally than anteroposteriorly. The deep glenoid fossa is indicative of a tight hinge-like articulation with the mandibular condyle. In *Tri*. *crassicuspis*, this fossa forms a saddle-shaped surface for articulation with the mandibular condyle, and has an ovoid ventral profile. The fossa also forms a smooth, continuous surface as observed in *Ar*. *primaevus*. In *Tri*. *crassicuspis*, the posterior portion of the zygomatic arch is robust.

A distinct and anteroposteriorly robust postglenoid process delimits the posterior wall of the glenoid fossa. In *Tri*. *crassicuspis*, the postglenoid process is considerably more developed distally than the preglenoid process, the latter which forms the anterior margin of the glenoid fossa. The distal margin of the preglenoid process is somewhat flattened, whereas that of the postglenoid process is broadly rounded, as in *Tri*. *quivirensis*. The postglenoid process is similar in overall morphology to that of *Ar*. *primaevus* in being massive and consisting of a mediolaterally expanded wall, situated along the whole posterior edge of the glenoid fossa. In *Tri*. *crassicuspis*, the anterior side of the postglenoid process forms a continuous surface with the glenoid fossa. The postglenoid process is approximately ‘U’-shaped and slightly asymmetrical in posterior view.

#### Mandible

**Fig 19:**
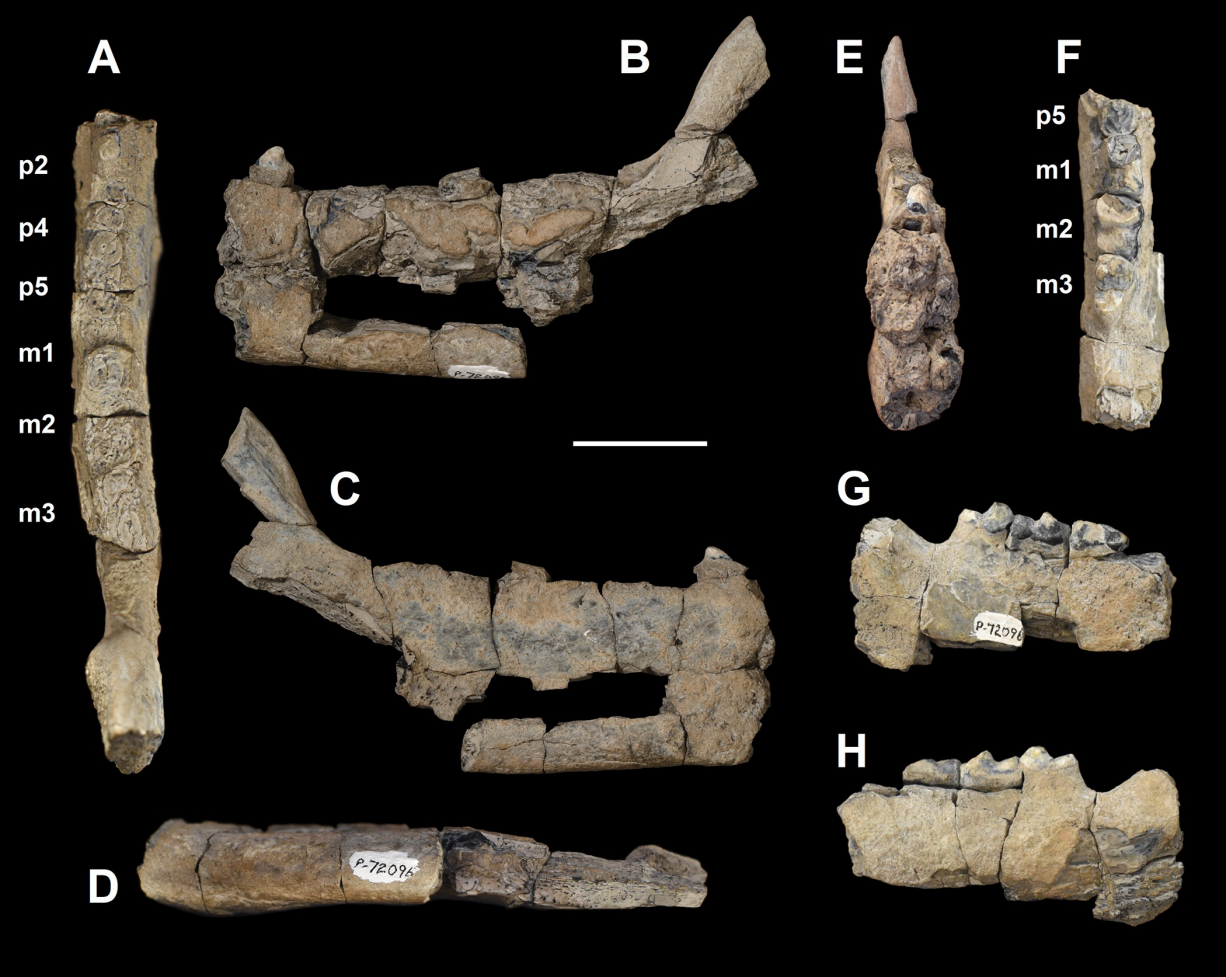
The hemi-mandibles of *Triisodon crassicuspis* (NMMNH P-72096). Partial left hemi-mandible (A-E): (A) occlusal view; (B) lateral view; (C) medial view; (D) ventral view; (E) anterior view. Partial right hemi-mandible (F-H): (F) occlusal view; (G) lateral view; (H) medial view. Scale bar: 30 mm.

**Fig 20:**
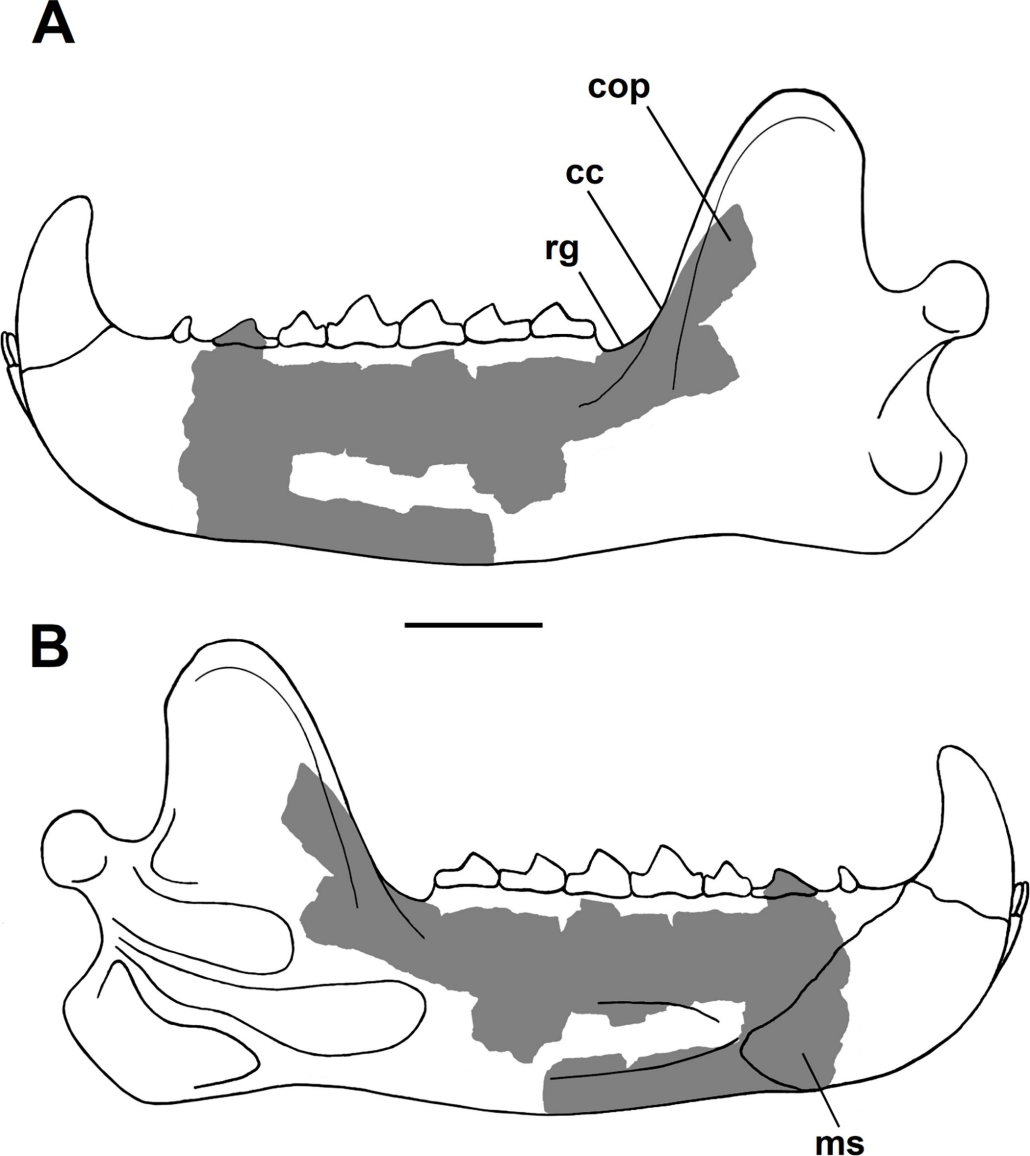
Annotated line drawing of the left hemi-mandible of *Triisodon crassicuspis* (NMMNH P-72096). (A) lateral view; (B) medial view. Gray areas represent areas for which material is known, white areas represent areas reconstructed hypothetically. The shapes and proportions of those elements unknown from *Tri*. *crassicuspis* are based on those of other “triisodontids”, specifically *Goniacodon levisanus* (AMNH 7798), *Tri*. *quivirensis* (AMNH 3352, figured in [17]) and *Eoconodon coryphaeus* (AMNH 16329, figured in [3]). Abbreviations: cc, coronoid crest; cop, coronoid process; ms, mandibular symphysis; rg, retromolar gap. Scale bar: 30 mm.

Both hemi-mandibles are known for NMMNH P-72096, which have been found separated, are highly fragmentary and are partially obscured by concretion (Figs 19 and 20). On the left hemi-mandible (Figs 19A–19E and 20), the lateral surface is considerably more concreted than the medial surface. NMMNH P-72096 preserves a significant portion of the left mandibular body, terminating just anterior to p2 with a jagged break, and the anterior portion of the coronoid process. Most of the mandibular ramus, including the condyloid process and angular process, is missing (Figs 19 and 20). The left hemi-mandible only preserves p2, with all the other posterior cheek teeth being broken above the roots (Fig 19A). No clear mental foramina are preserved on the left hemi-mandible. The right hemi-mandible is less complete and consists only of the posterodorsal portion of the mandibular body along with the base of the coronoid process (Fig 19F–19H). The right hemi-mandible only preserves m1-3.

The posterior portion of the mandibular symphysis is preserved on the medial surface of the left hemi-mandible. The mandibular symphysis terminates at the ventral margin of the mandible at the level between p2 and p4, the same position observed in *Goniacodon levisanus* (AMNH 7798), *Sinonyx jiashanensis* (IVPP V10760) and *“Dissacus” praenuntius* (UM 69305). This is more anterior than in *Triisodon quivirensis* (AMNH 3352) (in which the symphysis terminates at the level of the anterior margin of p5) or in *Eoconodon coryphaeus* (AMNH 16329) (in which the symphysis terminates at the level of the midpoint of p4). The symphysis terminates more posteriorly in all four of the aforementioned taxa than in *Arctocyon primaevus* (MNHN.F.CR2), in which it terminates at the level of the anterior margin of p2. In *Tri*. *crassicuspis*, the sutural surface for contact with the opposing hemi-mandible has a rugose texture, which is also the case in *Go*. *levisanus*, *E*. *gaudrianus* (AMNH 58116), *E*. *coryphaeus* and *Tri*. *quivirensis*. In life, both hemi-mandibles of *Tri*. *crassicuspis* were unfused as evident from the fact that they have been discovered separated, and the mandibular symphyses having a rugose texture [87]. This is the condition in *Tri*. *quivirensis*, *E*. *gaudrianus*, *E*. *coryphaeus* and *Go*. *levisanus* as well.

The alveolar plane of the mandible of *Tri*. *crassicuspis* is straight horizontally, as in *Go*. *levisanus*. The ventral edge of the mandibular body of *Tri*. *crassicuspis* is gently convex anteroposteriorly in medial or lateral aspect, with maximum height at m2 as in *Go*. *levisanus*. The mandibular body is also anteroposteriorly elongate, very deep dorsoventrally and conspicuously thickened mediolaterally along its ventral margin, from below the level of p2 back to m2. In medial or lateral aspect, the mandibular bodies of *E*. *coryphaeus*, *“D*.*” praenuntius* and *Si*. *jiashanensis* are considerably more gracile and proportionately shallower dorsoventrally than the condition in *Tri*. *crassicuspis*.

The mandibular body of *Tri*. *crassicuspis* is mediolaterally thickened in dorsal view. The alveolar process is wide, providing a mediolaterally broad shelf bordering the lateral surface of the molars and is where the *m*. *buccinator* originates [75]. In dorsal view, there is a slight medial shift of the posterior tooth row (i.e. the molars) relative to the anterior teeth. The lateral extension of the alveolar process and the medial shift of the posterior tooth row is comparable to that of *Ar*. *primaevus*. The ventral margin is smooth and mediolaterally convex in anterior aspect. In medial or lateral aspect, the mandibular body of *Ar*. *primaevus*, *Go*. *levisanus*, *E*. *coryphaeus* and *"D*.*" praenuntius* has a more anteroposteriorly convex ventral margin than the morphology exhibited by *Tri*. *crassicuspis*.

In medial or lateral aspect, the anterior border of the coronoid process is gently convex, resembling the condition in *Ar*. *primaevus* and *E*. *coryphaeus*. The coronoid process, as evident from its base, was moderately tall and oriented at an angle of approximately 120° to the horizontal plane of the mandible, as in *Ar*. *primaevus*. This angle is slightly smaller in *E*. *coryphaeus* (~110°), *E*. *gaudrianus* (~110°) and *Tri*. *quivirensis* (~115°), whereas that of *Si*. *jiashanensis* and *“D*.*” praenuntius* is slightly wider (~125° and ~130°, respectively). The anterior edge of the coronoid process consists of a large and mediolaterally wide coronoid crest. In anterior view, the coronoid crest is considerably thickened slightly dorsal to m3. The coronoid crest of *Tri*. *crassicuspis* is largely comparable to the morphology present in *Ar*. *primaevus*, *E*. *coryphaeus*, *E*. *gaudrianus*, *Tri*. *quivirensis*, which also bear distinct crests that terminate below m3. In *Tri*. *crassicuspis*, a retromolar gap is present between m3 and the coronoid process.

### Postcranial skeleton

#### Humerus

**Fig 21:**
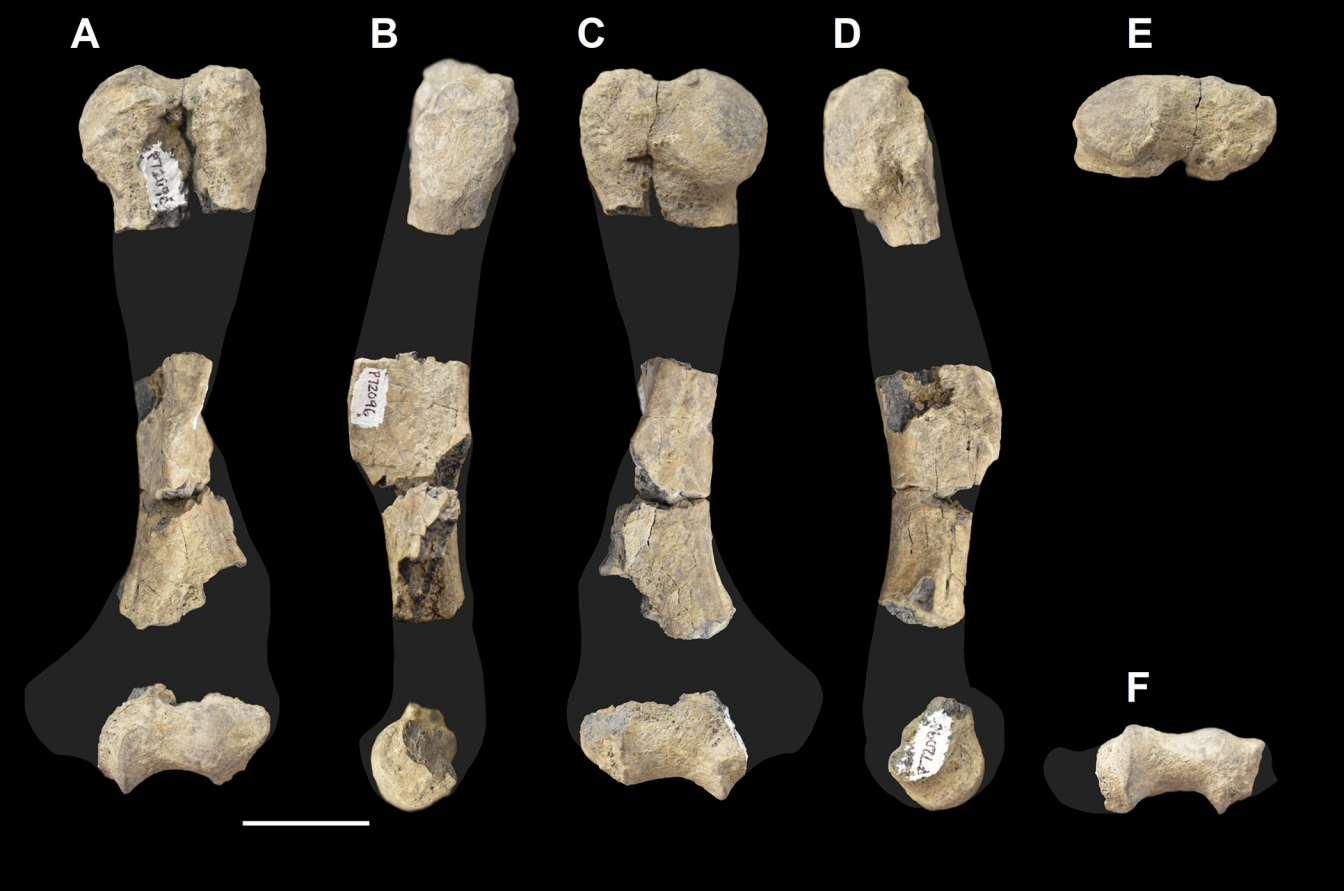
Partial left humerus of *Triisodon crassicuspis* (NMMNH P-72096). (A) anterior view; (B) lateral view; (C) posterior view; (D) medial view; (E) proximal view; (F) distal view. Scale bar: 30 mm.

**Fig 22:**
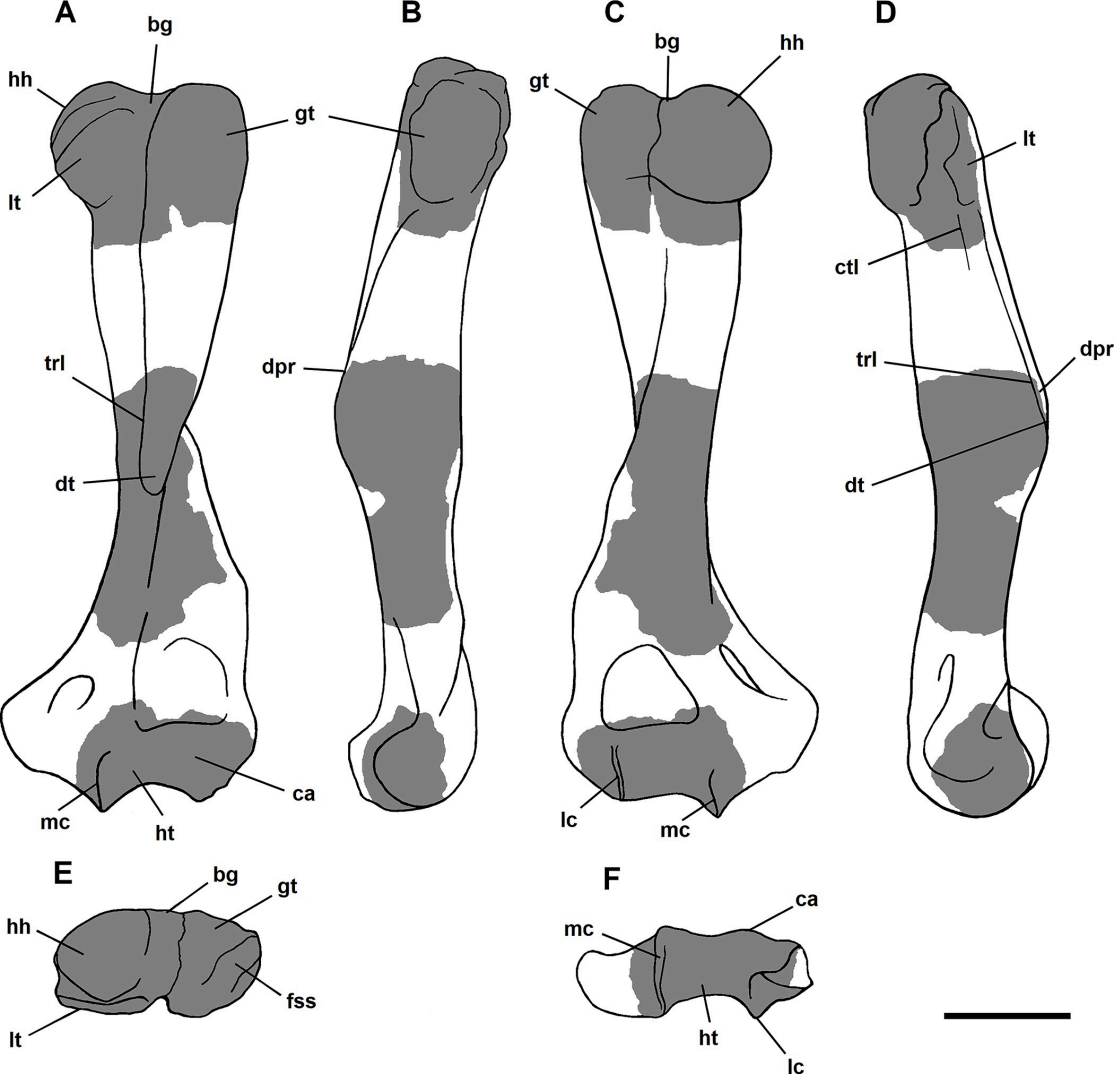
Annotated line drawing of the left humerus of *Triisodon crassicuspis* (NMMNH P-72096). (A) anterior view; (B) lateral view; (C) posterior view; (D) medial view; (E) proximal view; (F) distal view. Gray areas represent areas for which material is known, white areas represent areas reconstructed hypothetically. The areas reconstructed are based on comparisons to *Triisodon quivirensis* (AMNH 16559), *Eoconodon coryphaeus* (AMNH 764), *Goniacodon levisanus* (NMMNH P-16377) and *Arctocyon primaevus* (MNHN.F.CR17 and CR16, figured in [63]). Abbreviations: bg, bicipital groove; ca, capitulum; ctl, crest for insertion of the *m*. *teres major* and *m*. *latissimus dorsi*; dt, deltoid tuberosity; dpr, deltopectoral region; fss, fossa for the *m*. *supraspinatus*; gt, greater tubercle; hh, humeral head; ht, humeral trochlea; lc, lateral trochlear crest; lt, lesser tubercle; mc, medial trochlear crest; trl, tricipital line. Scale bar: 30 mm.

**Fig 23:**
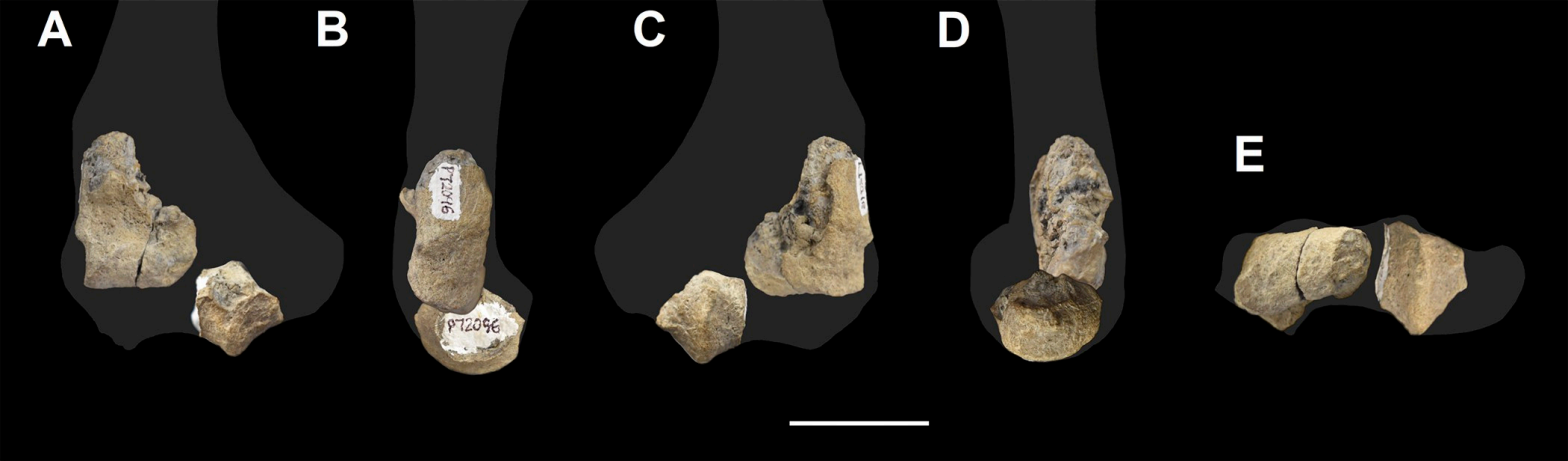
Partial right distal humerus of *Triisodon crassicuspis* (NMMNH P-72096). (A) anterior view; (B) lateral view; (C) posterior view; (D) medial view; (E) distal view. Scale bar: 30 mm.

Portions of both humeri of NMMNH P-72096 are preserved, but given their fragmentary nature, do not allow for a complete description of the anatomy. The left humerus is preserved in three parts: the proximal articular surface, the distal articular surface missing the medial epicondyle, and approximately the middle section of the diaphysis bearing the distal most portion of the deltopectoral region (Figs 21 and 22). The limitations of the humeral diaphysis fragment are just proximal to the deltoid tuberosity proximally and at the proximomedial border of the supracondylar ridge distally (Figs 21 and 22). The distal articular surface is broken proximal to the distal articular surface. The right humerus, on the other hand, is significantly less complete, consisting only of the distal articular surface preserved in two pieces, one including a partial radial fossa and a weathered capitulum, and the other including the medial crest of the trochlea and a partial medial epicondyle (Fig 23). In addition to NMMNH P-72096, portions of the humerus are also known for several other "triisodontid" taxa. The following description is largely based on the left humerus, and is supplemented by observation of the fragments from the right humerus available.

In overview, the morphology of the humerus of *Triisodon crassicuspis* appears to be relatively robust due to the presence of crests and their accompanying flanges on the diaphysis. The proximal articular surface is mediolaterally broader than anteroposteriorly deep. In *Tri*. *crassicuspis*, while the tubercles of the proximal articular surface are less distinct relative to those of *Arctocyon primaevus* (MNHN.F.CR17 and CR16) and *Hyaenodictis raslanloubatieri* (MHNT.PAL.2019.1.6), they are similarly well-defined as those observed in other Paleocene mammals of comparable body size, such as *"Dissacus" europaeus* (MNHN BR 12547) and *Periptychus carinidens* (NMMNH P-47693). The humerus of *Tri*. *crassicuspis* exhibits a well-developed greater tubercle. In anterior and posterior aspect, the greater tubercle extends proximally to roughly the same level as the humeral head, which is a similar condition to *Meles meles* (NMS.Z.RL111.97), *Gulo gulo* (NMS.Z.GH56.18) and *Tremarctos ornatus* (NMS.Z.2015.19). This contrasts to the condition found in *Orycteropus afer* (NMS.Z.2011.140.1), which possesses a greater tubercle projecting more proximally than the humeral head. Among fossil taxa, *Pe*. *carinidens* and *“D*.*” europaeus* display a similar condition to *Tri*. *crassicuspis*. In *Maelestes gobiensis* (PSS-MAE 607) and *Eoconodon gaudrianus* (AMNH 4029), the greater tubercle is proportionately more reduced and positioned slightly ventral to the humeral head.

In anterior and posterior aspect, the lateral surface of the greater tubercle shows a slightly convex outline. In proximal aspect, the greater tubercle of *Tri*. *crassicuspis* appears to be slightly deeper anteroposteriorly than mediolaterally broad. In proximal aspect, the greater tubercle is situated on the anterolateral side of the humeral head, situated closer towards the lateral surface. The greater tubercle of *Tri*. *crassicuspis* occurs as a structure set off from the humeral head, but to a lesser degree than the morphology exhibited by *E*. *gaudrianus*. On the proximal surface of the greater tubercle is a large, shallow groove that served as an attachment site for the *m*. *supraspinatus* [76,88]. In *Tri*. *crassicuspis*, the *m*. *infraspinatus* was most probably situated on the posterolateral surface of the greater tubercle, as observed in *Pachyaena ossifraga* (AMNH 4262) and *H*. *raslanloubatieri*, although we are unable to explicitly delimit its extent since some bone is missing on the posterior surface of the greater tubercle.

In *Tri*. *crassicuspis*, a well-defined, concave and smooth sulcus, which is an extension of the bicipital groove, separates the proximal surface of the greater tubercle from the humeral head. The anterior surface of the proximal articular surface where the bicipital groove extended is considerably weathered. The bicipital groove is shallow and wide, although as a result of postburial deformation, it appears slightly broader mediolaterally than it would have been in life. The bicipital groove of *Tri*. *crassicuspis* is not as well-defined as it is in *E*. *coryphaeus* (AMNH 764) or *D*. *navajovius* (AMNH 3359), appearing to be more similar to that of *Go*. *levisanus* and *Tri*. *quivirensis* (AMNH 16559). In *Tri*. *crassicuspis*, the greater tubercle does not overhang the bicipital groove, unlike the condition in *Ar*. *primaevus*. The bicipital groove of *Tri*. *crassicuspis* would have conveyed the long tendon of the *m*. *biceps brachii* [62,76,89,90]. In proximal aspect, the lateral border of the bicipital groove is demarcated by the medial border of the greater tubercle, which is distally extended to form a marked tricipital line, a rough line that delimits the deltopectoral crest medially.

The lesser tubercle of *Tri*. *crassicuspis* is flattened due to postburial deformation. Its anterior surface is obscured by matrix, but would have provided an attachment site for the *m*. *subscapularis*. The lesser tubercle is medially prominent. It also appears to be positioned more distally than the greater tubercle, as observed in *Ma*. *gobiensis*, *Ar*. *primaevus*, *E*. *gaudrianus*, *“D*.*” europaeus* and *Pe*. *carinidens*. Among extant taxa, this condition can be observed in *Me*. *meles*. The lesser tubercle is massive and is anteromedially appressed against the head of the humerus. The lesser tubercle of *Tri*. *crassicuspis* is relatively less salient than that of *Pa*. *ossifraga*. In comparison to *Tri*. *crassicuspis*, the lesser tubercle of *E*. *gaudrianus* and *Sinonyx jiashanensis* (IVPP 10760) appears to be proportionally smaller relative to the head of the humerus. On the medial surface of the humerus, just distal to the lesser tubercle, is a reduced, vertical and indistinct ridge that represents the attachment site for the *m*. *teres major* and *m*. *latissimus dorsi* [62,76,89,90]. As in *Pe*. *carinidens* and *H*. *raslanloubatieri*, this feature is weakly present in *Tri*. *crassicuspis*, but distinctly more salient in *Ar*. *primaevus*. This feature also does not appear to be particularly well-developed in *E*. *gaudrianus*.

The humeral head of *Tri*. *crassicuspis* is massive, hemispherical and proximoposteriorly directed, accounting for approximately 67% of the mediolateral width of the proximal articular surface. Among other “triisodontids”, a humeral head is known for *E*. *gaudrianus*, which appears to be more proximally directed and proportionately broader mediolaterally. In *Tri*. *crassicuspis*, the humeral head is rounded in posterior aspect and subrounded in proximal aspect. The humeral head is somewhat flattened anteroposteriorly, a condition associated with postburial deformation, making the degree of the projection of the head or how convex it was in life difficult to evaluate on NMMNH P-72096. The humeral head of *Tri*. *crassicuspis* is less rounded than that of *Ar*. *primaevus* and *E*. *gaudrianus*, but more so than that of *H*. *raslanloubatieri*. In *Tri*. *crassicuspis*, the humeral head is largely similar to that of “*D*.*” europaeus* regarding shape, with the mediolateral diameter being approximately equal to the proximodistal diameter. Among extant taxa, the humeral head of *Tri*. *crassicuspis* is most similar in development to that of *Me*. *meles*.

The humeral diaphysis of *Tri*. *crassicuspis* is more gracile than that of *Ar*. *primaevus* and *Pe*. *carinidens*, but less so than in *Goniacodon levisanus* (NMMNH P-16377) and *E*. *coryphaeus*, being most similar in robustness to that of *Tri*. *quivirensis*. In *Tri*. *crassicuspis*, the shaft itself is somewhat subtriangular in cross-section, whereas that of *Go*. *levisanus* and *Oxyclaenus cuspidatus* (NMMNH P-22034) is more subrounded. Above the supracondylar ridge, the humeral diaphysis of *Tri*. *crassicuspis* is somewhat flattened along its medial surface.

The deltopectoral region of *Tri*. *crassicuspis*, demarcated by the tricipital line and deltopectoral crest, is present on the proximal half of the diaphysis, is V-shaped and extends anteriorly, with a wide, anteriorly-facing surface for attachment of the *m*. *deltoideus* [76]. The tricipital line forms an attachment site for the *m*. *latissimus dorsi* and *m*. *teres major*, descending distally along roughly the proximal half of the deltopectoral region [76,89,91]. The deltopectoral crest resembles that of *Tri*. *quivirensis* in relative prominence, but is less prominent than that of *E*. *coryphaeus* and *Go*. *levisanus*. In *Ma*. *gobiensis*, the tricipital line and deltopectoral crest are placed high above the humeral diaphysis. Among extant taxa, the deltopectoral crest of *Tri*. *crassicuspis* is most similar in relative prominence to that of *Or*. *afer*.

In *Tri*. *crassicuspis*, a smooth shelf-like portion of bone comprises the deltopectoral region, as observed in *Go*. *levisanus*, *E*. *coryphaeus*, *Tri*. *quivirensis* and *Pe*. *carinidens*. Among extant taxa, this feature is observed in *Me*. *meles*, *Gu*. *gulo* and *Or*. *afer*. In contrast, the deltopectoral region of *Ar*. *primaevus*, *H*. *raslanloubatieri* and *Pa*. *ossifraga* consists of an additional crest (the median crest) extending across the proximodistal axis of the shelf, partitioning it into two separate muscle attachment sites.

In *Tri*. *crassicuspis*, the deltopectoral crest has well-expressed borders, though unlike the condition in *Ar*. *primaevus* and *Go*. *levisanus*, they do not comprise a salient overhanging lip, a feature that is also absent in all the extant comparison taxa. In *Tri*. *crassicuspis*, the medial and lateral boundaries of the deltopectoral crest converge distally to form a weakly pronounced deltoid tuberosity that protrudes anteriorly and represents the anteroposteriorly deepest portion of the diaphysis. This tuberosity corresponds to the distal part of the attachment site of the *m*. *deltoideus* [76,89]. The deltoid tuberosity of *Tri*. *crassicuspis* is similar in relative prominence to that of *Tri*. *quivirensis* and *Pe*. *carinidens*, although slightly less developed than that of *Ar*. *primaevus*, *Go*. *levisanus* and *E*. *coryphaeus*. Among extant taxa, it is most similar in development to that of *Or*. *afer*, being higher and more shelf-like than that of *Me*. *meles*, *Gu*. *gulo* and *Tre*. *ornatus*.

Most of the supinator ridge is missing, but as inferred from the cross-section and curvature of this structure, it was possibly prominent and extended posterolaterally as is the case in *Go*. *levisanus* and *E*. *coryphaeus*, and appears to be the case in *Tri*. *quivirensis*. The supinator ridge appears highly reduced in *Ox*. *cuspidatus*. The supinator ridge is a structure situated on the distal portion of the bone shaft, providing a large attachment site for the *m*. *extensor carpi radialis* anteriorly and the *m*. *anconeus* posteriorly [76,89,92]. In *Tri*. *crassicuspis*, it ascends up the diaphysis slightly below the horizontal level of the deltoid tuberosity.

On the anterior surface of the right distal humerus, proximal to the capitulum, the distolateral portion of the radial fossa is preserved, but is obscured by matrix and therefore difficult to discern how deep it was. As a result of damage and concretion, the olecranon fossa on the posterior surface is not preserved in NMMNH P-72096. There is no evidence of a supratrochlear foramen (as is present in *Pe*. *ossifraga*, *Si*. *jiashanensis* and *Pe*. *carinidens*, and likely present in *D*. *navajovius*) in *Tri*. *crassicuspis*. The preservation in the fossils available is insufficient to determine where the apex for the lateral epicondyle is. Very little of the medial epicondyle is preserved to be able to make any meaningful comments on the morphology or state definitively how developed it is, but what is available suggests that it extended from the level of the distal-most projection of the medial trochlear crest and formed a robust tuberosity. There is no fossa preserved for the *m*. *flexor carpi ulnaris*.

In *Tri*. *crassicuspis*, the distal articular surface is anteroposteriorly flattened. In anterior view, a smooth and almost continuous articular surface is formed by the humeral trochlea and rounded capitulum, separated by a poorly-defined zona conoidea, as in *Pe*. *carinidens*. The humeral trochlea of *Tri*. *crassicuspis* is mediolaterally narrower than the capitulum when taking the articulation between the humerus, radius and ulna into consideration, a morphology that is also present in all the comparison taxa.

*Tri*. *crassicuspis* has an asymmetrical humeral trochlea, as is the case in *Go*. *levisanus*, *E*. *coryphaeus*, *E*. *gaudrianus* and *Ar*. *primaevus*, with the articular area being mediolaterally narrower in anterior aspect than in posterior aspect. The humeral trochlea of *Ox*. *cuspidatus* is also asymmetrical, but there it has a large articular area that is more proximodistally elongate than in any of the comparison taxa observed. In posterior view, the humeral trochlea of *Tri*. *crassicuspis* consists of a moderately deep articular area formed by sharp, asymmetrical crests. The trochlea is also slightly deeper in posterior aspect than in anterior aspect, as in mesonychids like *H*. *raslanloubatieri*. The posterior surface of the humeral trochlea is mediolaterally broader than the anterior surface. The medial crest of *Tri*. *crassicuspis* is more oblique to the long axis of the humerus in anterior aspect than in posterior aspect. In *Tri*. *crassicuspis*, the medial crest of the trochlea is prominent and projects distally, while the lateral crest is relatively smaller and protrudes posteriorly. *Arctocyon* spp. has a relatively more salient lateral crest that is aligned with the vertical plane of the humeral diaphysis. In *Go*. *levisanus* and *E*. *coryphaeus*, the lateral crest is oblique to the vertical plane of the humeral diaphysis, so that it is roughly aligned with the medial crest, and it is possible this was also the case in *Tri*. *crassicuspis*.

In *Tri*. *crassicuspis*, the medial trochlear crest is slightly flared and protrudes mediodistally past the horizontal distal boundary formed by the medial epicondyle and the capitulum. This morphology is observed in all the comparison taxa, although it varies in the extent of development. In *Ma*. *gobiensis*, the medial crest extends somewhat distally but only just protrudes past the distal boundary of the medial epicondyle. In *Tri*. *crassicuspis*, the medial trochlea wall is both distally and mediolaterally expanded, making it appear more robust and flare less medially than in *Pe*. *carinidens*, *Go*. *levisanus*, *E*. *coryphaeus*, *E*. *gaudrianus* and *Ar*. *mumak* (YPM-PU 18703) among fossil taxa, or in *Or*. *afer*, *Gu*. *gulo* and *Tre*. *ornatus* among extant taxa. This condition in *Tri*. *crassicuspis* is also observed in mesonychids (*D*. *navajovius*, *Si*. *jiashanensis*, *Pa*. *ossifraga*, “*D*.*” europaeus* and *H*. *raslanloubatieri*). The medial crest of *Tri*. *crassicuspis* is subequal to the medial crest of these mesonychids regarding mediodistal protrusion, and is less robust mediolaterally. In *Ar*. *primaevus*, the medial crest is more proximodistally elongate than in any of these other taxa, descending further distally relative to the medial epicondyle and capitulum. Among extant taxa, the trochlea of *Tri*. *crassicuspis* is most similar in robustness and mediodistal protrusion to that of *Me*. *meles*, although it is slightly less distally expanded. In *Tri*. *crassicuspis*, the medial crest is mediolaterally broader in anterior aspect than in posterior aspect. In *Tri*. *crassicuspis*, it barely protrudes beyond the capitulum in anterior aspect.

In anterior aspect, a smooth, rounded articular surface forms the capitulum of *Tri*. *crassicuspis*, slightly elongated along its mediolateral axis as supported by the radial morphology. This contrasts to the more elongate and ovoid capitulum of *Pe*. *carinidens*, and the spindle-shaped capitulum of *Ar*. *mumak*. In distal aspect, the capitulum is convex and protrudes anteriorly more strongly than that of *E*. *coryphaeus*, *Ar*. *primaevus* and *Pe*. *carinidens*. Among extant taxa, the capitulum of *Tri*. *crassicuspis* is most similar in anterior protrusion to that of *Me*. *meles*. In anterior aspect, the capitulum is more rounded in *Tri*. *crassicuspis* than in *Ar*. *primaevus*, *Go*. *levisanus*, *E*. *gaudrianus*, *E*. *coryphaeus* and *Ox*. *cuspidatus*, being more similar to the spherical capitulum of mesonychids, namely *“D”*. *europaeus*, *H*. *raslanloubatieri*, *Si*. *jiashanensis* and *Pa*. *ossifraga*. Among extant taxa, the capitulum of *Tri*. *crassicuspis* is most comparable to the spherical capitulum of *Or*. *afer*, and very different from that of *Gu*. *gulo* and *Tre*. *ornatus*, in which it is more ovoid and nearly flat anteriorly. In distal aspect, the capitulum is slightly concave on the lateral side. In anterior aspect, the capitular tail is moderately well-developed in *Tri*. *crassicuspis*, but what is preserved of this structure only consists of a proximodistally wide lateral expansion of the capitulum.

#### Ulna

**Fig 24:**
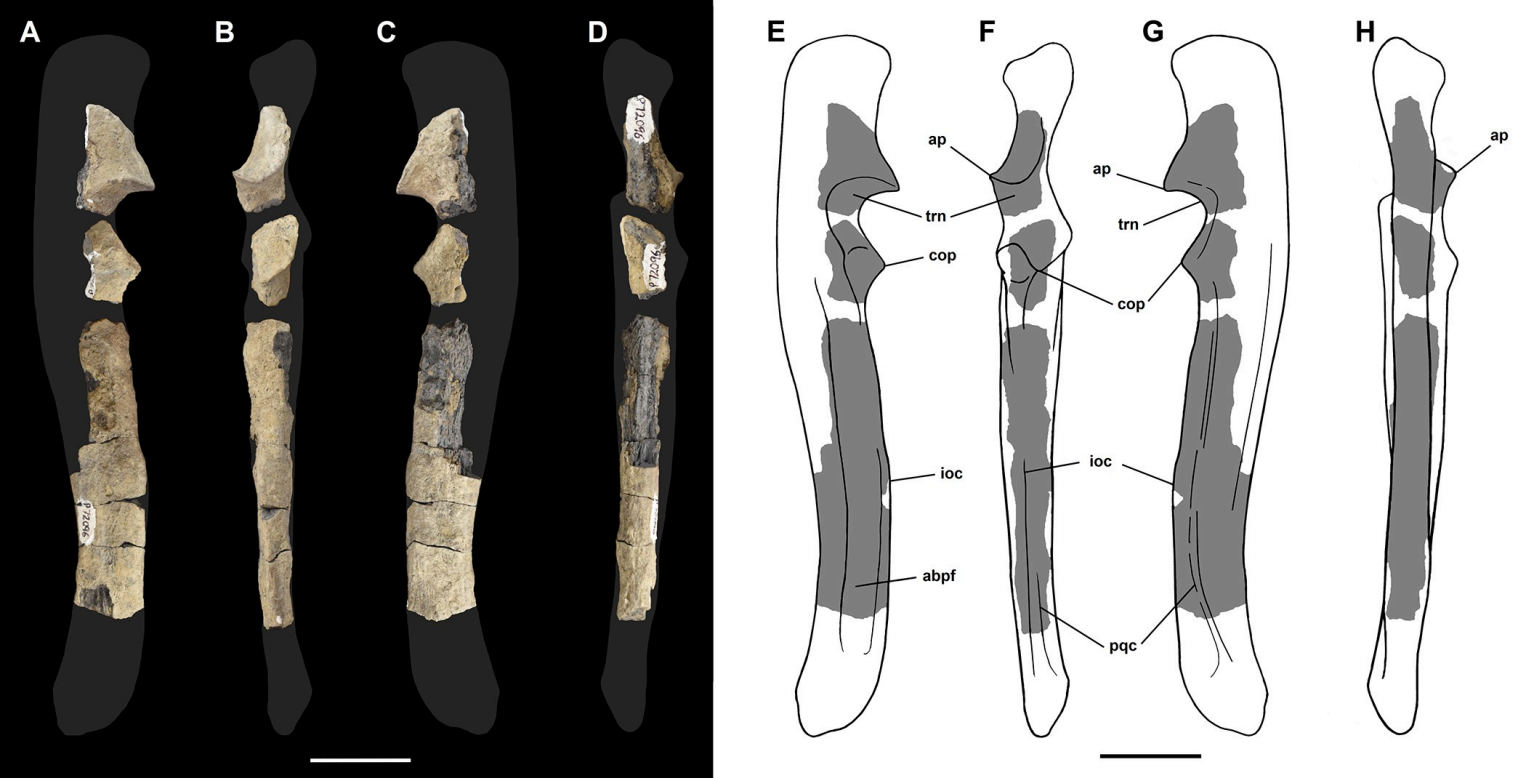
Ulna of *Triisodon crassicuspis*. Partial right ulna of *Tri*. *crassicuspis* (NMMNH P-72096) (A-D): (A) lateral view; (B) anterior view; (C) medial view; (D) posterior view. Annotated line drawing of the right ulna of *Tri crassicuspis* (E-H): (E) lateral view; (F) anterior view; (G) medial view; (H) posterior view. Gray areas represent areas for which material is known, white areas represent areas reconstructed hypothetically. The areas reconstructed are based on comparisons to *Goniacodon levisanus* (NMMNH P-16377), *Arctocyon primaevus* (MNHN.F.CR19, CR18, figured in Argot [63]) and *Pachyaena ossifraga* (AMNH 4262, figured in O’Leary and Rose [67]). Abbreviations: ap, anconeal process; cop, coronoid process; fapl, fossa for the *m*. *abductor pollicis longus*; ioc, interosseous crest; pc, pronator quadratus crest; trn, trochlear notch. Scale bars: 30 mm.

The following description is based on a partial right ulna that because of its fragmentary nature does not allow for a complete description of the anatomy. NMMNH P-72096 preserves a proximal articular surface that is broken across the trochlear notch, along with a large portion of the diaphysis, both from the left ulna (Fig 24). Most of the olecranon process is missing and the surface of the ulnar diaphysis is weathered in places, specifically on the distolateral and proximomedial portions. The diaphysis fragment is broken just distal to the coronoid process proximally, and midway at the pronator quadratus crest distally. The proximoposterior portion of the ulna, as well as the radial notch, is also missing.

The ulna of *Triisodon crassicuspis*, which consists of an anteroposteriorly deep and mediolaterally flattened diaphysis, is distinctly more gracile than the humerus. *Arctocyon primaevus* (MNHN.F.CR19, CR18) and *Periptychus carinidens* (NMMNH P-53998) are taxa which have a similar condition, but slightly more robust ulnae relative to their associated humeri. The ulnar diaphysis of *Tri*. *crassicuspis* is more robust and anteroposteriorly deeper than that of *Goniacodon levisanus* (NMMNH P-16377) and *Dissacus navajovius* (AMNH 3359), but less so than that of *Hyaenodictis raslanloubatieri* (MHNT.PAL.2019.1.7). Among fossil taxa, the ulna of *Tri*. *crassicuspis* is most akin to *Pachyaena ossifraga* (AMNH 4262) in terms of robustness. Among extant taxa, the ulna of *Tri*. *crassicuspis* is more gracile than that of *Orycteropus afer* (NMS.Z.2011.140.1) and *Tremarctos ornatus* (NMS.Z.2015.19), most similar in robustness to that of *Meles meles* (NMS.Z.RL111.97).

In lateral aspect, the ulna of *Tri*. *crassicuspis* has a barely convex ventral edge and a posteriorly concave posterior edge along the distal portion of the diaphysis. In anterior aspect, the ulna of *Tri*. *crassicuspis* appears to be straight in the proximodistal axis along much of its length, with the distal portion of the diaphysis being gently concave medially due to the development of the pronator quadratus crest on the medial surface. The ulna bears a salient proximal articular region. In medial view, a prominent anconeal process, with a sharp mediolateral convexity, confines the proximal border of the trochlear notch.

In *Tri*. *crassicuspis*, the anterior surface of the trochlear notch is saddle-shaped, similar to that of *Ar*. *primaevus* and *Pe*. *carinidens*. In *Tri*. *crassicuspis*, the proximal and distal boundaries of the trochlear notch are situated at an oblique angle relative to the proximodistal axis of the ulna, as in *Ar*. *mumak* (YPM-PU 18703), *Ar*. *primaevus* and *Pe*. *carinidens* among fossil taxa, and *Me*. *meles*, *Gu*. *gulo* (NMS.Z.GH56.18) and *Tre*. *ornatus* among extant taxa. This is unlike the condition in *Go*. *levisanus* among fossil taxa and *Or*. *afer* among extant taxa, where they are oriented approximately perpendicular to the proximodistal axis of the ulnar diaphysis.

*Tri*. *crassicuspis* has an asymmetrical anconeal process. The anconeal process of *Tri*. *crassicuspis* projects anteriorly relative to the ulnar diaphysis. The anconeal process is more prominent laterally in *Go*. *levisanus* and *Ar*. *primaevus* than in *Tri*. *crassicuspis*. Among extant taxa, *Or*. *afer* and *Me*. *meles* have an anteriorly prominent anconeal process, unlike the condition in *Gu*. *gulo* and *Tre*. *ornatus*. The anterior end of the anconeal process articulates with the olecranon fossa of the distal humerus during elbow extension [76]. In anterior view, the medial portion (which is directed medially for articulation with the medial crest of the humeral trochlea) is barely distinct from the body of the olecranon as in *Go*. *levisanus*. The medial portion is relatively more reduced than the lateral portion. The anconeal process is more anteriorly prominent than in *Go*. *levisanus*, *Ar*. *mumak* and *“D*.*” europaeus* (MNHN BR 12600), but less developed than in *H*. *raslanloubatieri*.

In anterior aspect, the anconeal process of *Tri*. *crassicuspis* is relatively narrow mediolaterally, similar to those of *Pe*. *carinidens* and *Go*. *levisanus*, and contrary to the condition in *Ar*. *primaevus*. A medial projection extending past the proximodistal midline of the ulna is absent from the anconeal process of *Tri*. *crassicuspis*, as in all the comparison taxa except for *Ar*. *primaevus*, which exhibits a small medial protrusion at the proximal boundary of the trochlear notch. In *Tri*. *crassicuspis*, the lateral portion of the anconeal process extends laterally to form a lip at the proximal-most boundary of the trochlear notch. It extends laterally only slightly, similar to the condition in *Pa*. *ossifraga*. Among extant taxa, this is also the case in *Or*. *afer*, and extends much less laterally in *Tri*. *crassicuspis* than in *Me*. *meles*. In *Ar*. *primaevus* and *H*. *raslanloubatieri*, the lateral portion of the anconeal process extends both proximally and laterally for articulation with the lateral trochlea rim of the distal humerus. This morphology is also present in *Me*. *meles*, *Gu*. *gulo* and *Tre*. *ornatus*.

The coronoid process of *Tri*. *crassicuspis* is large, narrow, not anteriorly prominent and likely overhung the medial edge of the ulnar diaphysis. The coronoid process articulates with the humeral trochlea, increasing the surface area of the elbow joint [76]. Among extant taxa, the coronoid process is most similar in relative prominence to that of *Me*. *meles* and *Gu*. *gulo*. The coronoid process has a pointed apex. In comparison to the height of the anconeal process, the coronoid process of *Tri*. *crassicuspis* is relatively taller than in *Go*. *levisanus*, *H*. *raslanloubatieri* and *Pe*. *carinidens*, being similar in height to that of *Ar*. *primaevus*. In *Tri*. *crassicuspis*, the anterior edge of the anconeal process is likely not surpassed in height by the coronoid process, unlike the condition in all the extant comparison taxa.

The ulnar diaphysis exhibits two distinct depressions along its preserved length for the insertion of several muscles. The lateral surface of the ulnar diaphysis bears a longitudinal, elongate and deep fossa that extends distally from the trochlear notch. This fossa extends over approximately 60% of the preserved anteroposterior depth of the ulnar diaphysis. In *Tri*. *crassicuspis*, the fossa is anteroposteriorly broad and restricted to the anterior portion of the lateral surface of the ulna as in *Ar*. *primaevus*, *Pa*. *ossifraga* and *Pe*. *carinidens* among fossil taxa, and *Or*. *afer* among extant taxa. This fossa served as an insertion for the *m*. *abductor pollicis longus* [76] and is posterolaterally demarcated by a salient, rounded, laterally-projecting crest where the muscle originated [88], a morphology also exhibited by *Ar*. *primaevus* and *Pa*. *ossifraga*. The *m*. *abductor pollicis longus* was likely well-developed as evident from the deep fossa for this muscle and the prominent laterally-projecting crest that delimits the fossa posteriorly. The proximal portion of the fossa is somewhat concreted, just distal to the trochlear notch.

In *Tri*. *crassicuspis*, the groove for the *m*. *abductor pollicis longus* is well-marked, but shallower than in *Ar*. *primaevus*, *Pa*. *ossifraga*, and *H*. *raslanloubatieri*, and deeper than in *Go*. *levisanus*. It is most similar in depth to that of *Pe*. *carinidens*. Among extant taxa, the fossa is shallower and the borders are less strongly defined in *Me*. *meles* and *Gu*. *gulo* than in *Tri*. *crassicuspis*. In *Go*. *levisanus*, the borders of the fossa are less strongly defined than in *Tri*. *crassicuspis*, but the surface area of the fossa is larger and mediolaterally broader. In *H*. *raslanloubatieri*, the borders of the fossa are more strongly defined than in *Tri*. *crassicuspis* and the fossa narrows distally. In *Tri*. *crassicuspis*, the depth and anteroposterior length of the fossa for the *m*. *abductor pollicis longus* is most closely approximated by that of *Or*. *afer* among the extant comparison taxa. In *Tre*. *ornatus*, it is relatively deeper and the borders are more strongly defined. The fossa for the *m*. *abductor pollicis longus* is anteriorly delimited by the interosseous crest, a structure forming a sharp ridge that is medially-inflected. This crest is especially pronounced on the distal half of the bone shaft. This crest also marks the origin of the interosseus membrane more proximally [63,64]. The interosseus crest is particularly well-developed in *Tri*. *crassicuspis*, in which it projects more anteriorly than that of *Go*. *levisanus*, *D*. *navajovius*, *Pa*. *ossifraga* and all the extant comparison taxa, and is similar in development to that of *Ar*. *primaevus*.

There appears to be a salient pronator quadratus crest on the medial surface of the distal portion of the diaphysis, strongly protruding medially. As in *Go*. *levisanus*, the pronator quadratus crest of *Tri*. *crassicuspis* extends into a broad rugose surface for the origin of the *m*. *pronator quadratus* [91]. The well-developed pronator quadratus crest increases the attachment surface for the *m*. *pronator quadratus*. The *m*. *pronator quadratus* occupies the gap between the ulna and the radius medially [76]. This muscle covers part of the medial surface of the diaphysis and the interosseus membrane [76]. The pronator quadratus crest is also salient in *Ar*. *primaevus*, but in contrast to that of *Go*. *levisanus* and *Or*. *afer*, in which it is relatively more reduced. Among extant taxa, the pronator quadratus crest of *Tri*. *crassicuspis* is most similar in relative prominence to that of *Me*. *meles*.

#### Radius

**Fig 25:**
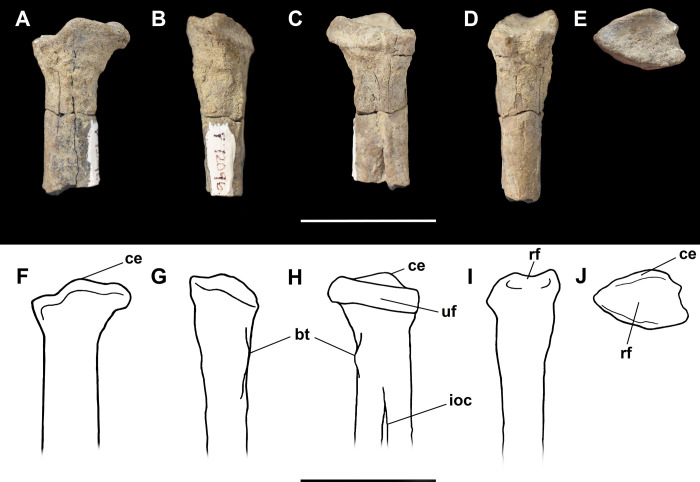
Radius of *Triisodon crassicuspis*. Partial left radius of *Tri*. *crassicuspis* (NMMNH P-72096) (A-E): (A) anterior view; (B) lateral view; (C) posterior view; (D) medial view; (E) proximal view. Annotated line drawing of the left radius of *Tri*. *crassicuspis* (F-J): (F) anterior view; (G) lateral view; (H) posterior view; (I) medial view; (J) proximal view. Abbreviations: bt, bicipital tuberosity; ce, capitular eminence; ioc, interosseous crest; rf, radial fovea; uf, ulnar facet. Scale bars: 30 mm.

NMMNH P-72096 preserves only the proximal portion of a left radius (Fig 25). The proximal fragment includes the radial head, the complete articular region and the diaphysis broken distal to the bicipital tuberosity. The bone is missing the crest for the *m*. *pronator teres* and the fossa for the *m*. *abductor pollicis longus*.

Compared to the proportionately slender ulna, the radius of *Triisodon crassicuspis* is a moderately robust bone, similar to the condition in *“Dissacus” europaeus* (MNHN BR 12547), *“D*.*” praenuntius* (UM 69305) and *Pachyaena ossifraga* (USGS 25292). This differs from the condition in *Goniacodon levisanus* (NMMNH P-16377) and *Sinonyx jiashanensis* (IVPP 10760), in which the radial shaft is distinctly slender relative to the robust ulna and the proximal articular surface is less proportionally wide mediolaterally. The radius is considerably less robust than that of *Ankalagon saurognathus* (AMNH 777), but slightly more so than in *Periptychus carinidens* (NMMNH P-47693). Among extant taxa, the radius is most similar in robustness to that of *Gulo gulo* (NMS.Z.GH56.18).

In *Tri*. *crassicuspis*, the proximal end of the radius is asymmetrical in anterior aspect, but less so than the condition in *Arctocyon primaevus* (MNHN.F.CR20) and in mesonychids (*Pa*. *ossifraga*, *Si*. *jiashanensis*, *D*. *navajovius* (AMNH 3359), *"D*.*" praenuntius* and *An*. *saurognathus*), with the lateral border oriented proximolaterally and the medial border oriented distomedially. In proximal aspect, the radial head of *Tri*. *crassicuspis* is wider mediolaterally than anteroposteriorly. The radial fovea, a central proximal concavity for articulation with the humeral capitulum [76], is shallowly concave as in *“D*.*” praenuntius*. The radial fovea of *Tri*. *crassicuspis* is shallower and more elongated on the mediolateral axis than in *Go*. *levisanus*. Among extant taxa, the central depression of the radial fovea in *Tri*. *crassicuspis* is similar in depth to that of *Meles meles* (NMS.Z.RL111.97) and *Gu*. *gulo*, and shallower than in *Orycteropus afer* (NMS.Z.2011.140.1) and *Tremarctos ornatus* (NMS.Z.2015.19).

In proximal aspect, the radial fovea appears subtriangular in shape, but this is likely a result of postburial deformation and in life would have been more ovoid in outline, a morphology also exhibited by *Pe*. *carinidens*, *Ar*. *primaevus*, *Ar*. *mumak* (YPM-PU 18703), *“D*.*” praenuntius* and *Go*. *levisanus*. In *Tri*. *crassicuspis*, the medial border of the radial fovea consists of a steeply-inclined, distally directed margin for articulation with the humeral capitulum and lateral trochlea crest [76]. The medial surface of the proximal articular surface exhibits some damage.

The radial fovea is surrounded by a large, proximally expanded, crescent-shaped lateral flange, similar to the condition in *Me*. *meles* and *Tre*. *ornatus*. This flange would have articulated with the lateral extension of the capitulum [63,76]. The capitular eminence of *Tri*. *crassicuspis* is widely rounded and also prominent, as is the case in *Go*. *levisanus*, *"D*.*" praenuntius* and *Si*. *jiashanensis*. In *Tri*. *crassicuspis*, it comprises a marked proximal process on the anterior margin of the proximal articular surface and it protrudes slightly above the lateral flange as in *Go*. *levisanus*, but unlike any of the extant comparison taxa. This process corresponds to the trochlear notch of the ulna [76]. The radial head of *Tri*. *crassicuspis* lacks an indentation lateral to the capitular eminence, unlike the condition in *Go*. *levisanus* and *"D*.*" praenuntius*. The ulnar facet, which articulated with the radial notch of the ulna [76], is smooth and clearly demarcated on the posterior side of the radial head. The ulnar facet is proximodistally narrow, mediolaterally broad and runs along the whole posterior margin of the radial head, similar to *“D*.*” europaeus*. The ulnar facet of *Tri*. *crassicuspis* is also slightly convex as in *Go*. *levisanus*, *“D*.*” praenuntius* and *Pe*. *carinidens*.

On the posterolateral surface of the radial diaphysis of *Tri*. *crassicuspis*, just distal to the neck, is a mediolaterally narrow bicipital tuberosity (= radial tuberosity) which is differentiated from the neck exclusively by its posterior projection. The bicipital tuberosity forms a slightly elevated attachment site for the tendon of the *m*. *biceps brachii* [76]. Although the bicipital tuberosity of *Tri*. *crassicuspis* is more proximodistally elongate, it is less prominent than the more rectangular (the long axis which is proximodistal) bicipital tuberosity of *Go*. *levisanus*, *Ar*. *primaevus* and *An*. *saurognathus*, the tuberosity of the latter two taxa which are regarded as “weak” [63,65].

In *Tri*. *crassicuspis*, the proximal portion of the radial diaphysis is relatively straight in lateral or medial views, which is comparable to *Pe*. *carinidens*, *"D*.*" praenuntius*, *“D*.*” europaeus* and *D*. *navajovius*. This is unlike the condition in *Ar*. *primaevus*, *Go*. *levisanus*, *Si*. *jiashanensis*, *Pa*. *ossifraga* and all the extant comparison taxa, in which the radius is more anteriorly convex in lateral and medial views. In *Tri*. *crassicuspis*, the radial shaft is somewhat anteroposteriorly flattened in cross-section but expands proximally in the anteroposterior and mediolateral directions. This contrasts to the approximately circular radial diaphysis of *Ar*. *mumak*, *Go*. *levisanus* and *Pe*. *carinidens*. In *Tri*. *crassicuspis*, on the posterior surface of the radial diaphysis and distal to the bicipital tuberosity, is the beginning of a prominent scar which possibly marks the insertion of the proximal portion of a well-developed interosseous membrane or crest [63,64,91].

#### Femur

**Fig 26:**
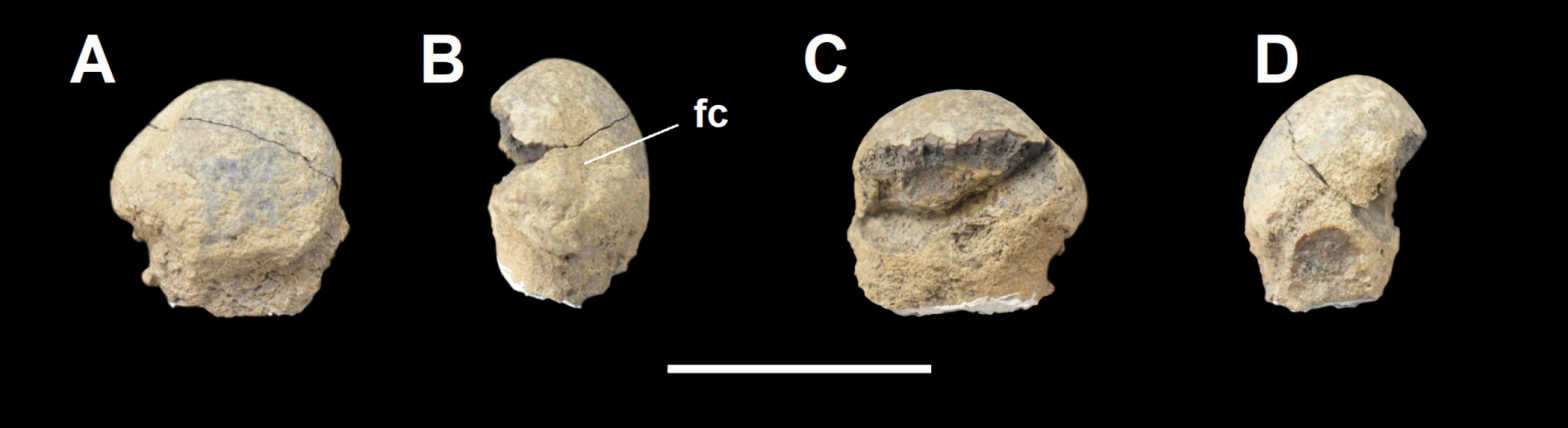
Right femoral head of *Triisodon crassicuspis* (NMMNH P-72096). (A) posterior view; (B) medial view; (C) anterior view; (D) lateral view. Abbreviations: fc, fovea capitis. Scale bar: 30 mm.

The only element in NMMNH P-72096 unambiguously identified as pertaining to the femur is the right femoral head (Fig 26).

The femoral head is large and would have originally been hemispherical in shape, as in *Periptychus carinidens* (NMMNH P-19430) and *Arctocyon primaevus* (MNHN.F.CR17, CR16), but has undergone postburial deformation, becoming more mediolaterally compressed (Fig 26B and 26D). The hemispherical shape contrasts to the relatively ovoid shape of *Ar*. *mumak* (YPM-PU 18703), the latter which has a flattened distal edge [64]. The articular surface is smooth and restricted to the head, not extending onto the femoral neck medially. In *Triisodon crassicuspis*, the articular surface on the posteromedial side of the femoral head is excavated by a well-defined and relatively deep fovea capitis (Fig 26B), which provided an insertion for the ligamentum teres [91]. The fovea capitis is ovoid-shaped and distally expanded, although it does not interrupt the posteromedial border of the femoral head, as in *Ar*. *mumak* and *Ar*. *primaevus*, but unlike *Pe*. *carinidens*. The anteroposteriorly narrow fovea capitis could have also resulted from postburial deformation.

## Discussion

The new specimen we describe here, NMMNH P-72096, gives key insights into the dental, cranial, and postcranial anatomy of *Triisodon crassicuspis*, which in turn allows us to explore its body size, functional morphology, diet, locomotor behavior, and ecology.

### Body mass estimation

Cope described *Triisodon* spp. as being “about the size of a wolf” in his original description [44], on the basis of a partial dentary. The postcranial material now available indicates that this is likely a relatively accurate comparison for the body size of *Tri*. *crassicuspis*, and further allows us to make more accurate quantitative estimates of its body mass.

We used a series of equations that take proxy data from tooth or postcranial measurements to predict the body mass of *Tri*. *crassicuspis* (Table 4). These give a range of estimates: the various dental equations are generally consistent in predicting a body mass in the range of ca. 32–44 kg, whereas the one postcranial proxy indicates a considerably higher body mass of ca. 66 kg, although of the same order of magnitude as the dental equations. All evidence considered, we argue that the dental estimates are more likely to be accurate, for two reasons. First, a series of dental equations generated by different authors [77–79] give a relatively narrow range of estimates, showing that the dentition gives a consistent signal. Second, although it has been argued that long bone measurements provide the most accurate body mass estimates, because these bones physically hold the animal up against gravity [80,93], there are circumstances where these postcranial proxies can be misleading. Specifically, Campione and Evans [80] explored how their equations do not produce such accurate results for fossorial mammals, which might have more robust bones to anchor digging musculature, not to support body mass. Indeed, in *Tri*. *crassicuspis*, the deltopectoral region of the humerus is particularly developed, which we argue below is at least in part related to fossorial abilities. The humeral circumference proxy may, therefore, be overestimating body mass.

In summary, we consider it most likely that *Tri*. *crassicuspis* had a body mass in the vicinity of 32–44 kg. Among extant mammals, this was approximately the mass of a striped hyena (*Hyaena hyaena*) as well as a gray wolf (*Canis lupus*) [94]. In comparison to other mammals of the contemporary fauna, *Tri*. *crassicuspis* was of approximately a similar mass to *Periptychus carinidens* (ca. 42.5 kg [62]) and *Pantolambda bathmodon* (ca. 42.3 kg [95]), both mammals which could have been potential prey. While 32.19 kg may be an underestimate, the Legendre [78] all-mammal equation is the most suitable equation to use due to the lack of certainty regarding the phylogenetic placement of *Triisodon* spp. and extant analogues, and only requires measurements from a single tooth.

### Craniodental functional morphology

From NMMNH P-72096, it is possible to gain insights into the characteristics of the osteological anatomy of *Triisodon crassicuspis* and start to qualitatively understand its functional morphology, with reference to possible ecologies and locomotor behaviours. Regarding diet, the molars of “triisodontids” like *Triisodon* spp. have been proposed to be suggestive of an omnivorous diet with some degree of carnivory [14,96], and our observations of the craniodental anatomy support the hypothesis that they did indeed have multiple carnivorous specializations.

With a flat, shearing edge, the lower and upper canines occlude closely. The buccal margin of the lower molars is overhung by the metacone and paracone of the upper molars. The talonid basin in the lower molars occludes with the protocone of the upper molars, resulting in a ‘mortar-and-pestle’ arrangement. The gaps between the lingual portions of the upper molars accommodate the trigonid of the lower molars. As revealed by examination of the somewhat sectorial dentition of *Tri*. *crassicuspis* and other “triisodontids”, the family exhibits evolutionary trends towards carnivory, particularly the massive canines with distinct shearing surfaces, lower molars with a much higher trigonid relative to the talonid, the prominent protoconids on the lower molars, mesiodistally aligned hypoconulid crest on the lower molars, and well-developed posterior premolars with distally projecting cusps and distinct shearing edges formed by cristae.

In *Tri*. *crassicuspis* and other “triisodontids”, however, the dentition was yet to be well developed for carnivory given the lack of specialized carnassial blades present in extant carnivorans. As the number of premolars and molars is not reduced in order to slice meat efficiently, because of the retention of several cusps characteristic of the tribosphenic molar such as the metaconid [97], and because the cheek teeth are less laterally compressed, the dentition of *Tri*. *crassicuspis* is poorly developed for carnivory in comparison to that of hypercarnivorous mammals such as felids. The dentition of extant hypercarnivorous mammals has an increased shearing component in relation to the crushing component [97–99], and possess highly reduced grinding structures including the protocones, paraconules and metaconules [97], unlike the condition in *Tri*. *crassicuspis*.

The broad, low-crowned, bunodont molars of *Tri*. *crassicuspis* differ substantially from those of extant specialized carnivorans, being most analogous to those of ursids [100]. This suggests that, similar to these extant omnivores, the diet of *Tri*. *crassicuspis* possibly consisted of fruit, nuts, seeds and invertebrates, in addition to small- to large-sized vertebrates and carrion [101,102]. Lastly, the ventrally prominent postglenoid process deepens the glenoid fossa and would have therefore helped prevent dislocation of the jaw whenever high bite forces were applied at closing angles, as in extant carnivorous durophages [103]. This, along with inflated, conical molar cusps, suggest that *Tri*. *crassicuspis* was capable of a powerful bite, potentially for engaging in durophagy or to compensate for the dentition of *Tri*. *crassicuspis* and other “triisodontids” being, as yet, less specialized than that of extant carnivorans.

### Postcranial functional morphology

In *Triisodon* spp., the anterior prominence of the deltopectoral region resulted in powerful shoulders. The prominence would have also enhanced the moment arm of the deltopectoral musculature and therefore aided in increasing the rotational and adductive ability of those muscle bodies [88]. The weakly pronounced deltoid tuberosity present in *Triisodon* spp. does not rule out the inference of well-developed deltopectoral musculature. However, it is possible that it may be less specialized or well-developed in *Triisodon* spp. than that seen in *Arctocyon primaevus*, *Goniacodon levisanus* and *Eoconodon coryphaeus*. Moreover, the robust humerus suggests a relatively well-developed digging ability; the shape of the proximal ulna and its relative thickness in the distal region resemble that of an animal well-adapted for digging [104].

The elbow joint of *Tri*. *crassicuspis* provides a wealth of data about the functional morphology of the forelimb. The near continuity between the capitulum and humeral trochlea, with a poorly-developed zona conoidea, suggests an elbow that was well-stabilized by muscles and ligaments instead of by its osteological anatomy [63,105]. The asymmetrical trochlea, along with the distally extended medial trochlear crest, is suggestive of a habitually slightly abducted forelimb, as in *Ar*. *primaevus* [63] and *Periptychus carinidens* [62]. The rounded capitulum of *Tri*. *crassicuspis* appearing to be continuous with the trochlea suggests that mobility at the elbow was limited [106].

The large coronoid process of the ulna would have supported the humerus and was involved in weight bearing, a common feature in terrestrial mammals [89]. The ulna exhibits well-defined muscle attachment sites for a well-developed *m*. *abductor pollicis longus* and *m*. *pronator quadratus*, associated with powerful extension of the forelimb [63], a motion which may be associated with short bursts of rapid terrestrial movement like running and leaping [82]. In this way, powerful forelimb extension puts the elbow joint at risk of dislocation, although the humeral trochlea of *Tri*. *crassicuspis* has a prominent medial crest, which contributes to joint stability during this extension to prevent dislocation [107]. The ovoid radial fovea of *Tri*. *crassicuspis* would have stabilized the radial joint and considerably limited the extent of mobility at the humeroradial joint, specifically restricting the degree of pronation-supination [108].

The humerus of *Tri*. *crassicuspis* bears a close resemblance to that of *Pe*. *carinidens* in anatomical features such as the lesser tubercle being positioned more distally than the greater tubercle, and the weakly pronounced deltoid tuberosity. The distal forelimb, specifically the ulna, closely resembles that of the Paleocene mesonychid *Pachyaena ossifraga* in robustness and in anatomical features including the development of the anconeal process and the posterior border of the fossa for the *m*. *abductor pollicis longus*. Although *Tri*. *crassicuspis* is less robust than *Ar*. *primaevus* and *Pe*. *carinidens*, it is by no means gracile. Whether *Tri*. *crassicuspis* relied more heavily on its forelimbs for dexterity and agility remains uncertain until more complete material from the hindlimb is found.

In describing the anatomy of *Tri*. *crassicuspis*, it is clear that it bears a close resemblance to many related medium- to large-sized Paleocene mammal taxa, ranging from other “triisodontids” to mesonychids. Among extant taxa, the osteology of *Tri*. *crassicuspis* bears superficial similarities to that of extant taxa adapted to digging including the badger (*Meles meles*) and the aardvark (*Orycteropus afer*), specifically in the combination of mobility and strength of the forelimb. The specific anatomical similarities shared between *Tri*. *crassicuspis* and those extant taxa include the shape of the humerus and the ulna, a smooth, shelf-like deltopectoral region, a rounded humeral capitulum, an anteriorly prominent anconeal process and a relatively thick distal ulna. We therefore conclude that *Tri*. *crassicuspis* was a terrestrial animal with at least moderate digging ability.

## Conclusions

“Triisodontids” are typically rare elements of Paleocene faunas and the specimen NMMNH P-72096 represents one of the most complete “triisodontid” specimens ever recovered. These descriptions and comparisons based on this specimen provide the first insights into the cranial and postcranial anatomy of the relatively large “triisodontid” *Triisodon crassicuspis*. Though the teeth were less well-developed for carnivory relative to extant hypercarnivorous mammals, the dental adaptations suggest that the diet of *Triisodon* spp. incorporated vertebrates nonetheless. Moreover, the dentition of *Triisodon* spp. seems to have been more adapted to carnivory compared to other “triisodontids” including *Goniacodon* spp. and *Eoconodon* spp. [96]. It is the postcrania of *Tri*. *crassicuspis* described here which is most illuminating. The forelimb morphology of *Tri*. *crassicuspis* is that of a large and powerful terrestrial animal. The forelimb bones of *Tri*. *crassicuspis* are robust relative to its size, more so than other members of the same family including *Go*. *levisanus* (ca. 4.4 kg [95]), *E*. *coryphaeus* (ca. 12.6 kg [95]) and *Oxyclaenus cuspidatus* (ca. 6.74 kg [95]).

**Fig 27:**
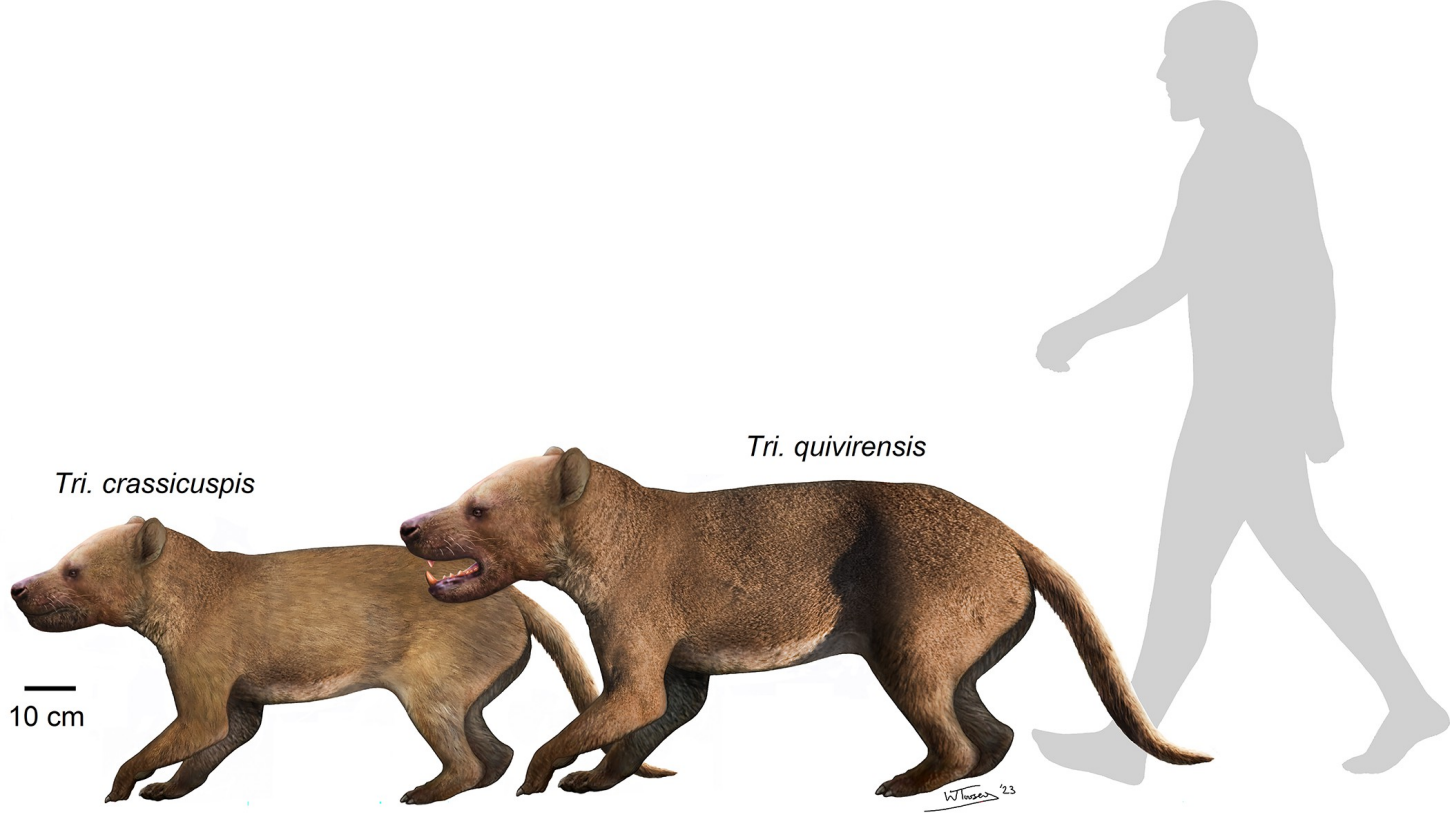
Speculative reconstructed life appearance of *Triisodon crassicuspis* and *Triisodon quivirensis*, with an average human male for size comparison. Original artworks by William Toosey, 2023.

Some of the most notable aspects of the functional morphology of *Tri*. *crassicuspis* postcrania are in the humerus and elbow joint. There was limited mobility at the elbow joint itself, being less mobile than in extant scansorial animals, but was stable during forceful extension of the forelimb. *Tri*. *crassicuspis* was seemingly terrestrial with limited scansorial ability but some digging ability, which is equally supported by the heavy weight estimated for this species (ca. 32–44 kg based on dental proxies). Since *Tri*. *crassicuspis* stratigraphically overlaps with *Tri*. *quivirensis*, they must have been partitioning some type of large predatory mammal niche, albeit briefly, as *Tri*. *crassicuspis* appears to be even more restricted in time (Fig 27). Like many other Paleocene mammals, *Tri*. *crassicuspis* was far from being uniformly generalized [109], amalgamating a basic placental body plan with various carnivorous specializations, a testament to the evolutionary adaptability of mammals to thrive in ever-changing environments.

## Supporting information

S1 Fig Humeri of extant comparative taxa. Left humerus of *Meles meles* (NMS.Z.RL111.97) (A-F): (A) anterior view; (B) lateral view; (C) posterior view; (D) medial view; (E) proximal view; (F) distal view. Left humerus of *Gulo gulo* (NMS.Z.GH56.18) (G-L): (G) anterior view; (H) lateral view; (I) posterior view; (J) medial view; (K) proximal view; (L) distal view. Left humerus of *Orycteropus afer* (NMS.Z.2011.140.1) (M-R): (M) anterior view; (N) lateral view; (O) posterior view; (P) medial view; (Q) proximal view; (R) distal view. Left humerus of *Tremarctos ornatus* (NMS.Z.2015.19) (S-X): (S) anterior view; (T) lateral view; (U) posterior view; (V) medial view; (W) proximal view; (X) distal view. Scale bar: 30 mm.
(TIF)

S2 Fig Ulnae of extant comparative taxa. Right ulna of *Meles meles* (NMS.Z.RL111.97) (A-D): (A) lateral view; (B) anterior view; (C) medial view; (D) posterior view. Right ulna of *Gulo gulo* (NMS.Z.GH56.18) (E-H): (E) lateral view; (F) anterior view; (G) medial view; (H) posterior view. Right ulna of *Orycteropus afer* (NMS.Z.2011.140.1) (I-L): (I) lateral view; (J) anterior view; (K) medial view; (L) posterior view. Right ulna of *Tremarctos ornatus* (NMS.Z.2015.19) (M-P): (M) lateral view; (N) anterior view; (O) medial view; (P) posterior view. Scale bar: 30 mm.
(TIF)

S3 Fig Radii of extant comparative taxa. Left radius of *Meles meles* (NMS.Z.RL111.97) (A-E): (A) anterior view; (B) lateral view; (C) posterior view; (D) medial view; (E) proximal view. Left radius of *Gulo gulo* (NMS.Z.GH56.18) (F-J): (F) anterior view; (G) lateral view; (H) posterior view; (I) medial view; (J) proximal view. Right radius of *Orycteropus afer* (NMS.Z.2011.140.1) (K-O): (K) anterior view; (L) lateral view; (M) posterior view; (N) medial view; (O) proximal view. Left radius of *Tremarctos ornatus* (NMS.Z.2015.19) (P-T): (P) anterior view; (Q) lateral view; (R) posterior view; (S) medial view; (T) proximal view. Scale bar: 30 mm.
(TIF)

S1 File Raw dental measurements for the lower dentition of *Triisodon quivirensis*, *Triisodon crassicuspis* and *Goniacodon levisanus* in Fig 16. (XLS)
