# Supplementary figures and images for: The osteology of Triisodon crassicuspis (Cope, 1882): New insights into the enigmatic “archaic” placental mammal group “Triisodontidae”
Source: PLoS One. 2024 Nov 11;19(11):e0311187. doi: 10.1371/journal.pone.0311187 (PMC11554371; doi:10.1371/journal.pone.0311187)

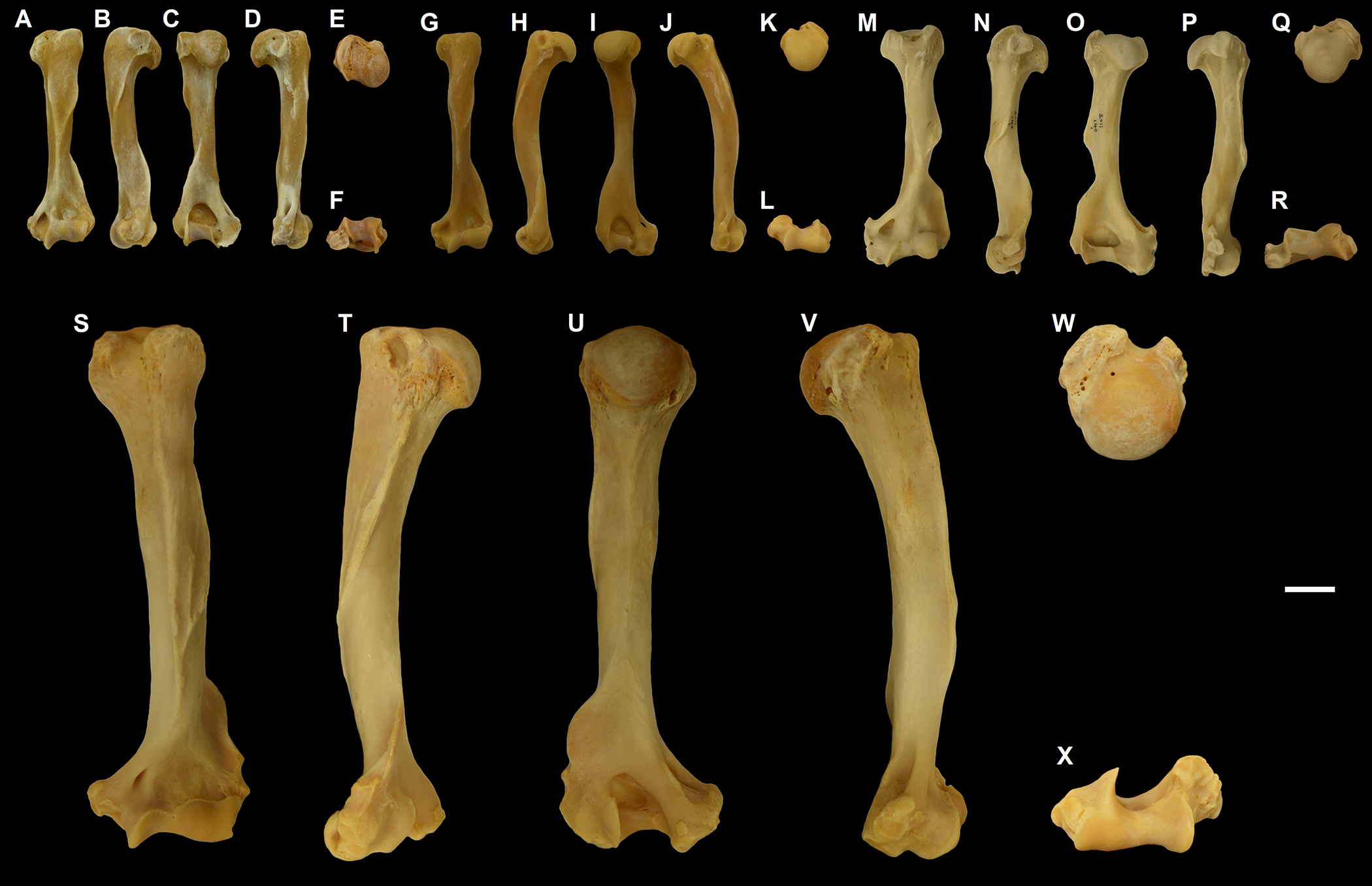

Supplement: S1 Fig — Left humerus of Meles meles (NMS.Z.RL111.97) (A-F): (A) anterior view; (B) lateral view; (C) posterior view; (D) medial view; (E) proximal view; (F) distal view. Left humerus of Gulo gulo (NMS.Z.GH56.18) (G-L): (G) anterior view; (H) lateral view; (I) posterior view; (J) medial view; (K) proximal view; (L) distal view. Left humerus of Orycteropus afer (NMS.Z.2011.140.1) (M-R): (M) anterior view; (N) lateral view; (O) posterior view; (P) medial view; (Q) proximal view; (R) distal view. Left humerus of Tremarctos ornatus (NMS.Z.2015.19) (S-X): (S) anterior view; (T) lateral view; (U) posterior view; (V) medial view; (W) proximal view; (X) distal view. Scale bar: 30 mm. (TIF) [file pone.0311187.s001.tif]

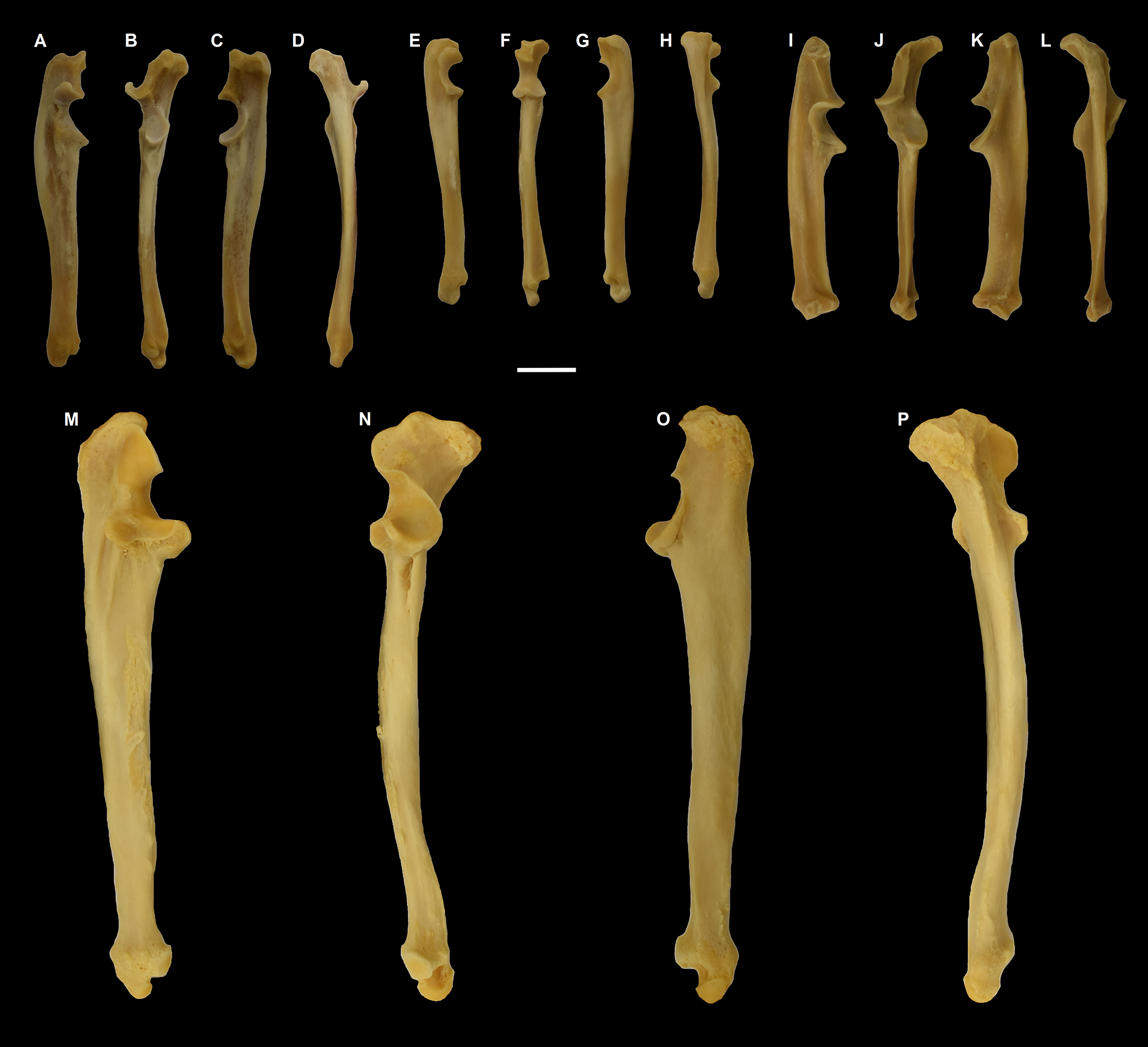

Supplement: S2 Fig — Right ulna of Meles meles (NMS.Z.RL111.97) (A-D): (A) lateral view; (B) anterior view; (C) medial view; (D) posterior view. Right ulna of Gulo gulo (NMS.Z.GH56.18) (E-H): (E) lateral view; (F) anterior view; (G) medial view; (H) posterior view. Right ulna of Orycteropus afer (NMS.Z.2011.140.1) (I-L): (I) lateral view; (J) anterior view; (K) medial view; (L) posterior view. Right ulna of Tremarctos ornatus (NMS.Z.2015.19) (M-P): (M) lateral view; (N) anterior view; (O) medial view; (P) posterior view. Scale bar: 30 mm. (TIF) [file pone.0311187.s002.tif]

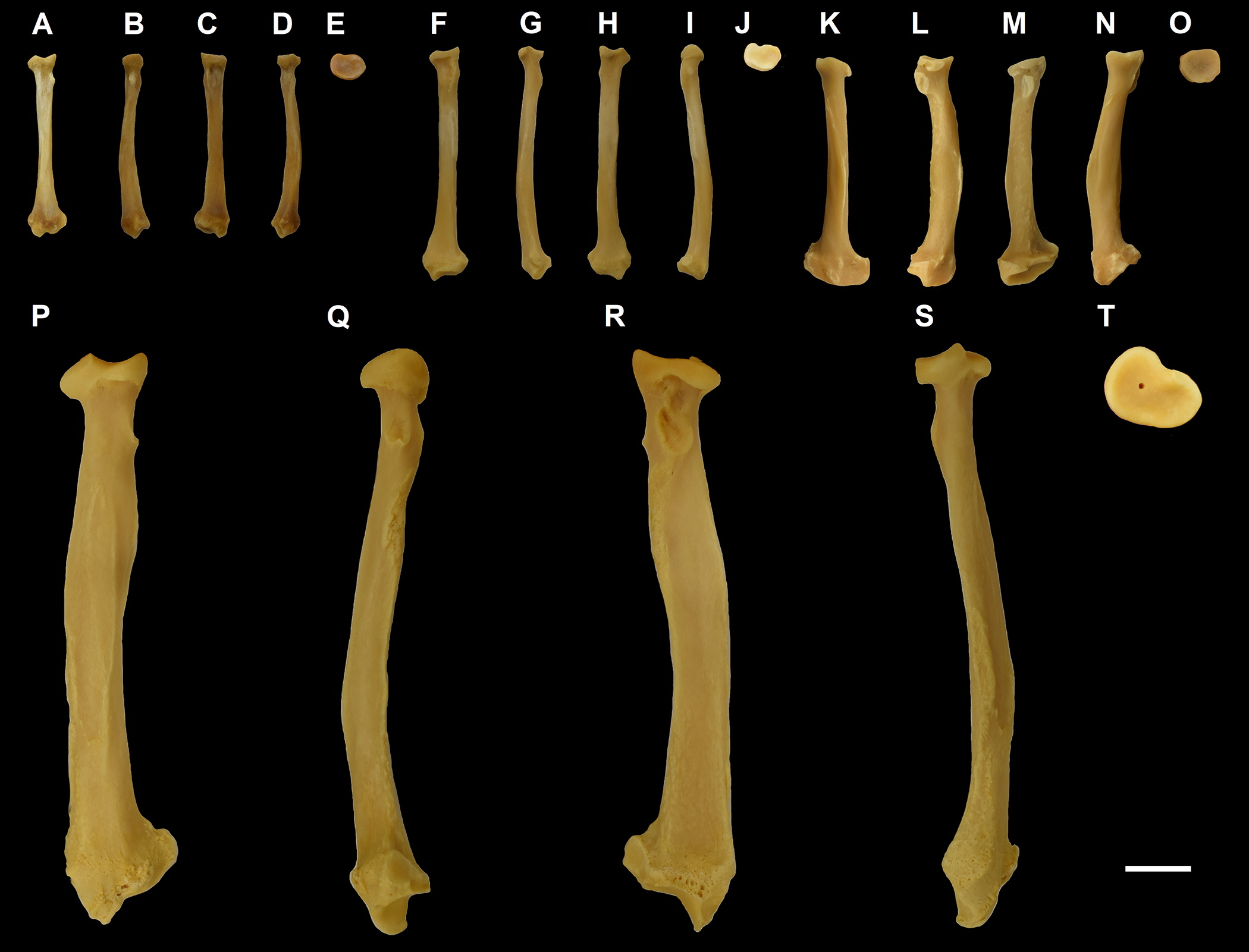

Supplement: S3 Fig — Left radius of Meles meles (NMS.Z.RL111.97) (A-E): (A) anterior view; (B) lateral view; (C) posterior view; (D) medial view; (E) proximal view. Left radius of Gulo gulo (NMS.Z.GH56.18) (F-J): (F) anterior view; (G) lateral view; (H) posterior view; (I) medial view; (J) proximal view. Right radius of Orycteropus afer (NMS.Z.2011.140.1) (K-O): (K) anterior view; (L) lateral view; (M) posterior view; (N) medial view; (O) proximal view. Left radius of Tremarctos ornatus (NMS.Z.2015.19) (P-T): (P) anterior view; (Q) lateral view; (R) posterior view; (S) medial view; (T) proximal view. Scale bar: 30 mm. (TIF) [file pone.0311187.s003.tif]
